# Mapping recommended strategies to promote active and healthy lifestyles through physical education classes: a scoping review

**DOI:** 10.1186/s12966-022-01278-0

**Published:** 2022-03-28

**Authors:** Alexsandra da Silva Bandeira, Fabricio Cesar de Paula Ravagnani, Valter Cordeiro Barbosa Filho, Victor José Machado de Oliveira, Edina Maria de Camargo, Maria Cecília Marinho Tenório, Paula Fabricio Sandreschi, Priscila Cristina dos Santos, Virgílio Viana Ramires, Pedro Curi Hallal, Kelly Samara Silva

**Affiliations:** 1 Department of Physical Education, Federal University of Santa Catarina, Florianopolis, Brazil; 2 Institute of Education, Science and Technology of Mato, Grosso do Sul – Campus Campo, Grande, Brazil; 3 Federal Institute of Education, Science and Technology of Ceara, Aracati Campus, Aracati, Brazil; 4 Federal University of Amazonas, Faculty of Physical Education and Physiotherapy, Manaus, Brazil; 5 Federal Technological University of Paraná, Department of Physical Education, Curitiba, Brazil; 6 Federal Rural University of Pernambuco, Department of Physical Education, Recife, Brazil; 7 Ministry of Health of Brazil, Primary Health Care Secretariat, Health Promotion Department, General Coordination To Promote Physical Activity and Intersectoral Initiatives, Brasilia, Brazil; 8 Sul-Rio-Grandense Federal Institute of Education, Science and Technology, Camaquã Campus, Brazil; 9 Federal University of Pelotas, Rua Marechal Deodoro 1160, Pelotas, RS 96020-220 Brazil

**Keywords:** School, Physical education, Strategies, Physical activity, Health

## Abstract

**Background:**

Understanding which strategies have been recommended for the promotion of active and healthy lifestyles through physical education (PE) classes can guide PE policies and practice. Therefore, we summarized worldwide recommendations regarding strategies for PE classes that have aimed to promote active and healthy lifestyles among school-aged children and adolescents.

**Methods:**

The Preferred Reporting Items for Systematic Reviews and Meta-Analyses extension for Scoping Reviews (PRISMA-ScR) guidelines were utilized. A literature search was carried out in June 2020 in eight peer-reviewed literature databases, in addition to searches in institutional and personal libraries. The eligibility criteria included any online document that included recommendations targeting any dimension of PE classes (e.g., policy and environment, curriculum, appropriate instruction, student assessment, and strategies that interact with PE) published since 2000.

**Results:**

In total, 2,408 potentially eligible documents were screened. Of these, 63 were included in the final analysis. The recommended strategies were as follows: six referred to policy and environment (valuing PE, higher frequency and duration of classes, inclusive PE classes, mandatory daily classes, evaluation of PE classes, and qualified teachers), five to curriculum (structure, type of content, cross-cutting themes, and components that improve PE classes), four to appropriate instruction (promotion of physical activities, inclusion of social issues, employment of the use of innovative technologies, and organization of the teaching–learning process), and three to student assessment (understanding human movement concepts, evaluation of contents, and assessment methods to develop an active and healthy lifestyle).

**Conclusion:**

Twenty-one strategies recommended for PE classes linked to five dimensions aimed at different target populations were identified. Over half were linked to the dimensions of policy and environment and appropriate instruction. PE is recommended to be mandatory and valued at all educational levels, with weekly frequency that contributes to an active and healthy lifestyle. This review shows that guaranteeing different experiences beyond sports, improving social inclusion, using innovative technologies, and providing adequate materials and spaces to be important challenges and ways to guide policies, programs, and new research in this field of knowledge.

Open Science Framework Registration: https://osf.io/harwq/

**Supplementary Information:**

The online version contains supplementary material available at 10.1186/s12966-022-01278-0.

## Introduction

Physical education (PE) is a curricular component in schools that offers students the opportunity to acquire PE-related knowledge and skills contributing to integral human development [1]. Broad and properly oriented PE classes contribute to students developing their physical literacy, which is defined as holistic learning involving motor/physical [2], psychological/cognitive (e.g., self-esteem and lower levels of anxiety) [3], and social skills (e.g., cooperation, proactivity, and establishment of friendships) [4] obtained through life, applied in movement and physical activity contexts [5]. PE also contributes to expanding the cultural experience and political values (ethical, aesthetic) for a critical training of students [6]. Moreover, quality PE may help people engage in physical activity during the school phase of one’s life and beyond, allowing them to apply the skills obtained through PE classes [7].

At the same time, there are important challenges worldwide in ensuring the right to PE classes. Data from the Global School-Based Student Health Survey, a population-based survey developed with the assistance of the World Health Organization and Centers for Disease Control and Prevention, that involved 206,417 adolescents from 65 countries showed that two out of ten students were not enrolled in PE classes, and only 25% of students were enrolled in three or more PE classes weekly [8]. The last United Nations Educational, Scientific and Cultural Organization global survey on PE conducted in 2013 included data from 232 countries/autonomous regions worldwide and reinforced that 97% of countries have legal requirements for PE classes at some age/stage or phase of compulsory schooling, but only 71% of countries adhered to implementation in accordance with legal/mandatory obligations or expectations [9].

In addition to the quantity of PE classes, there is a need to consider the quality of PE, which might be addressed by investigating the successful implementation strategies to reveal the main elements (for policy–practice purposes) and strategies directed to stakeholders for this transformation setting (i.e., policymakers, stakeholders, teachers, students, and families). In other words, there is a need to understand how, when, where, and how much PE can help students’ and society’s health, education, and development [10, 11]. Government and non-government institutions have recommended several strategies for guiding public policy decisions and practice for PE classes, aiming to improve the active and healthy lifestyles of students [10, 12]. These strategies can be related to (i) the recognition and structure involved in the discipline (i.e., policy and environment), (ii) the didactic–pedagogical actions of teachers (i.e., appropriate instruction), (iii) the elaboration of the educational contents and guidelines (i.e., curriculum), and (iv) the evaluation of the development and progress of students (i.e., assessment) [12]. In addition, strategies that interact with PE classes (i.e., health education actions at school) have been recommended for PE to be a promoter element of a health-promoting school [13].

Although the strategies recommended in plans and policies to promote physical activity are known and have been summarized [14, 15], to our knowledge, no study has synthesized which strategies are recommended specifically for PE classes. For policies, summarizing such recommendations can support the elements that stakeholders and politicians may consider for governmental and institutional decisions connected with this discipline. The actors involved in pedagogy (especially teachers and students) can understand the different strategies that improve pedagogical practice and build positive experiences with human movement concepts [16], which are essential for health promotion. By summarizing the information through a scoping review, it is possible to understand the breadth of recommended strategies and the gaps in policy documents, guiding new research on the subject.

Therefore, this study intends to fill research and policy gaps contributing to the improvement of PE classes. The purpose of this systematic scoping review was to summarize worldwide recommendations regarding PE class strategies (policies and environment, appropriate instruction, curriculum, and assessment) aimed at promoting active lifestyles among school-aged children and adolescents. We sought to answer the following research question: What strategies for PE classes have been recommended worldwide to promote active lifestyles among school-aged children and adolescents?

## Methods

### Protocol and registration

**Fig. 1:**
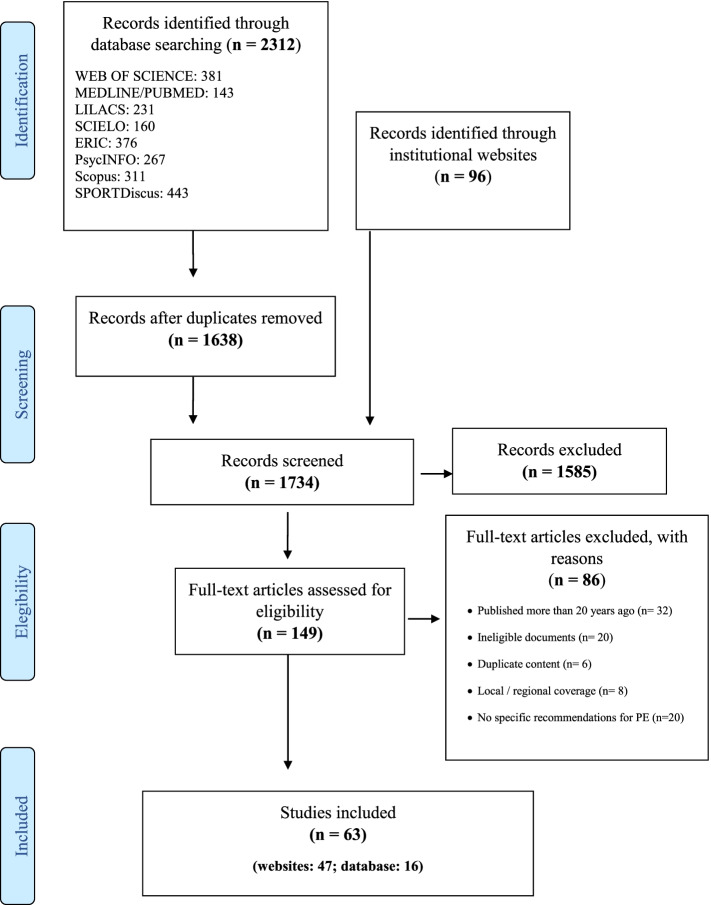
Flow diagram for the scoping review process according to the PRISMA-ScR

To answer our research question, a scoping review was conducted, providing an overview of the literature on our topic of interest (promoting active lifestyles through PE), indicating the extent of the studies available, key characteristics, emerging evidence, and research gaps, with the aim of contributing to research and policy agendas [17]. For this review, we used the PRISMA-ScR (PRISMA extension for Scoping Reviews, Supplementary Material 1) checklist [17] (Fig. 1). The protocol was registered in the Open Science Framework (https://osf.io/harwq/).

### Eligibility criteria

This scoping review involved the following steps: 1) identifying the research question, 2) identifying the relevant studies, 3) selecting the studies, 4) extracting the data, and 5) summarizing and reporting the findings. The guiding question was “What strategies related to PE classes have been recommended to promote active lifestyles in students?”.

The eligibility criteria for documents were organized into four groups, considering the recommended mnemonic (Population, Concept, and Context) for research questions of scoping reviews [18] and with the inclusion of the type of publication (Table 1).

**Table 1:** Eligibility criteria for the scoping review

Elements	Eligibility criteria
Type of publications	Guidelines, positions, plans, handbooks, or other documents; documents published by organized civil societies, professional organizations, governmental or non-governmental and non-profit organizations with national or international scope; documents published since 2000 until the date of research; and documents in Portuguese, English or Spanish were considered eligible. For documents that were updates of a previous document, only the last version was considered eligible
Population	Documents directed at school-age kids from elementary to high school were considered eligible. The Brazilian grades for kindergarten, elementary, middle and high school were based on the National Curriculum Guidelines [19]. Documents that addressed specific teaching modalities for target populations were not included (e.g., Indians, rural education, and special education for children with physical or intellectual disabilities)
Concept	Strategies were analyzed based on the four essential components of PE used by SHAPE [12], in addition to a fifth dimension elaborated by our group, as follows: 1) Policy and environment – related to changes in the number of weekly classes, class duration, size, time, spaces, equipment, and pedagogical structure as well as school rules regarding PE exemptions, substitutions, or punishments 2) Curriculum – focused on curricular plans and standards, such as the content, units, lessons, and activities to be taught during PE 3) Appropriate instruction – related to the use of instructional practices provided by the physical educator that encourage teacher and student involvement, participation and inclusion, and the use of materials, equipment, and technologies to promote maximum physical activity during classes 4) Student assessment – related to the measurement and monitoring of what students learn during PE; 5) Other practices related to PE – related to the actions that occur beyond the PE classes, but are intrinsically linked to the promotion and development of students’ physically active and healthy lifestyle
Context	This review included documents that presented any recommendation for the promotion of a physically active and healthy lifestyle though PE classes. Documents with recommendations that reported multi-component strategies related to PE, that is, in addition to PE classes, were also included. Documents that included high-performance sports were not considered

### Information sources

For our document search, we used three approaches that included peer-reviewed literature databases, searches in institutional websites, and personal libraries. For peer-reviewed literature, the search was carried out on June 4, 2020, in eight scholarly databases/repositories: Web of Science, MEDLINE/PubMed, LILACS, SCIELO, ERIC ProQuest, PsycINFO, Scopus, and SPORTDiscus. Second, electronic database searches were complemented by a search on websites of 27 national and international institutions that have contributed or contribute to PE in national or international contexts, referring to different regions of the world. The websites were accessed in June 2020 (see Supplementary Material 2). In addition, the reference lists of the included studies and recent publications were examined to identify potential studies that could also be included in the review.

### Search

The search terms were obtained and refined by consulting relevant publications connected with the topic of interest of this study. The search was structured using free text and Medical Subject Headings (MESH) terms. The strategy was based on the most relevant elements of the study problem (i.e., PE, school, physical activity and its related outcomes, health, and education) and the type of publication (i.e., reviews and recommendations; see Supplementary Material 3). The terms were combined using Boolean operators (OR, AND, and AND NOT) and truncation symbols. The search strategies followed the recommendations of a past peer review of electronic search strategies [20].

### Selection of sources of evidence

The titles of the selected references from the searched databases were imported into an EndNote library. Duplicate works were excluded using a reference manager. The library, which contained all potentially eligible titles, was worked on separately by two reviewers (AB and FR) with experience in systematic reviews. The selection process comprised two phases: 1) title and abstract reading and 2) full text reading. In both phases, a consensus meeting was held to discuss potentially eligible studies that met the study inclusion criteria (Table 1). In case of discrepancies between the reviewers, a third reviewer (VBF) was consulted for a decision.

One reviewer (VBF) searched for potential websites to conduct a document search. The potentially eligible documents were organized in a datasheet and analyzed by two reviewers (AB and FR) before being included in the final review.

### Data charting process and data items

Data was extracted by two independent reviewers (AB or FR). Each reviewer was responsible for half of the selected documents and the complete extraction was revised by another (VBF). In addition, discrepancies between reviewers were resolved through consensus meetings. A standardized excel spreadsheet was created and included the following data: the name of the institution involved, the document’s geographical coverage, the year the document was published, the document’s goals and description, the target population (school administrators, principals, teachers, parents, and students), the PE dimensions (policy and environment, curriculum, appropriate instruction, assessment, and information on other practices related to PE; see Supplementary Material 4).

### Summarizing and reporting the results

**Fig. 2:**
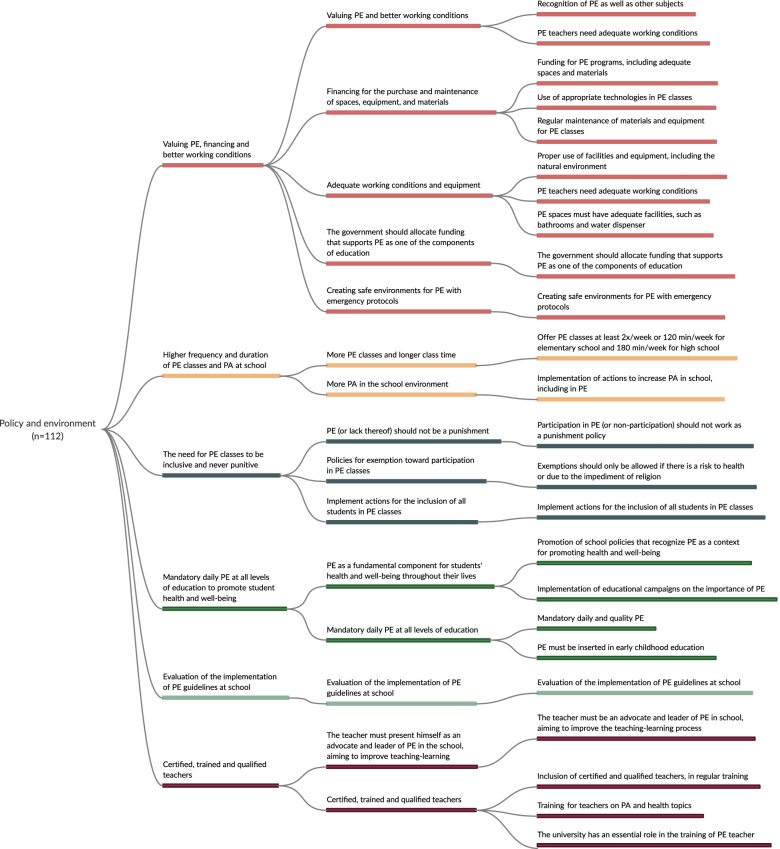
Mapping of recommended strategies to promote active and healthy lifestyles in the dimension of policy and environment

**Fig. 3:**
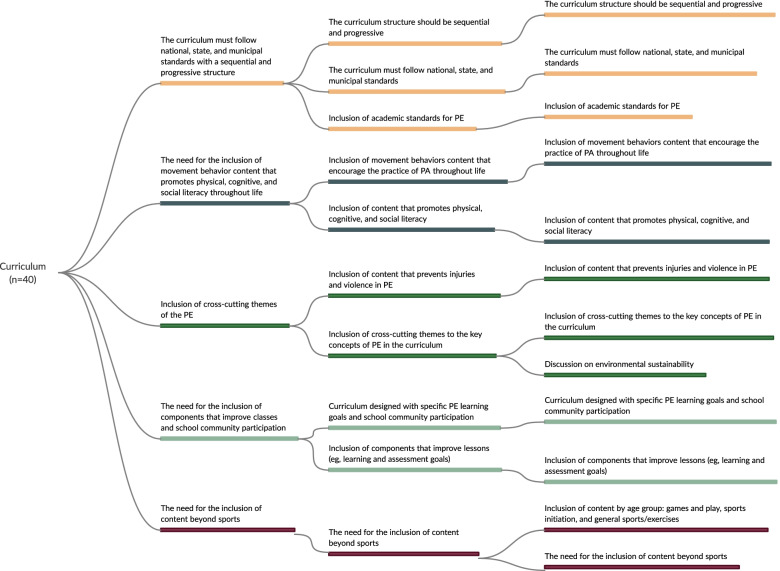
Mapping of the recommended strategies to promote active and healthy lifestyles in curriculum dimension

**Fig. 4:**
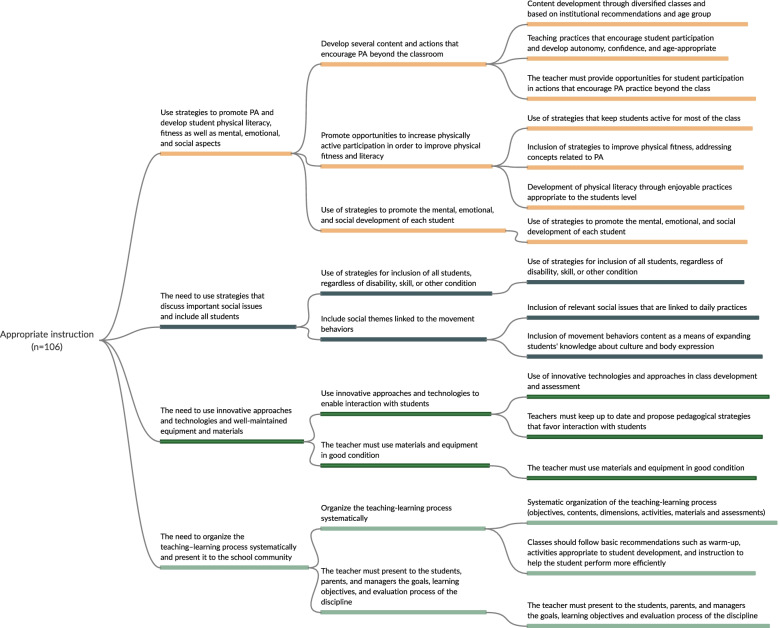
Mapping of recommended strategies to promote active and healthy lifestyles in the dimension of appropriate instruction

**Fig. 5:**
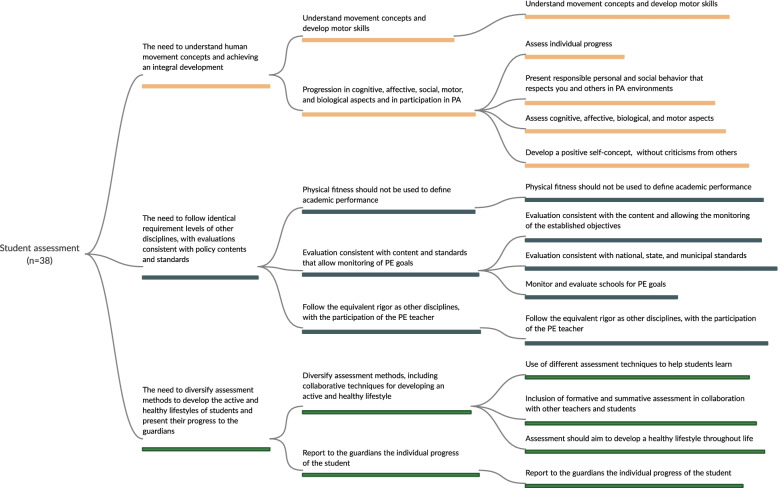
Mapping of recommended strategies to promote active and healthy lifestyles in the dimension of student assessment

**Fig. 6:**
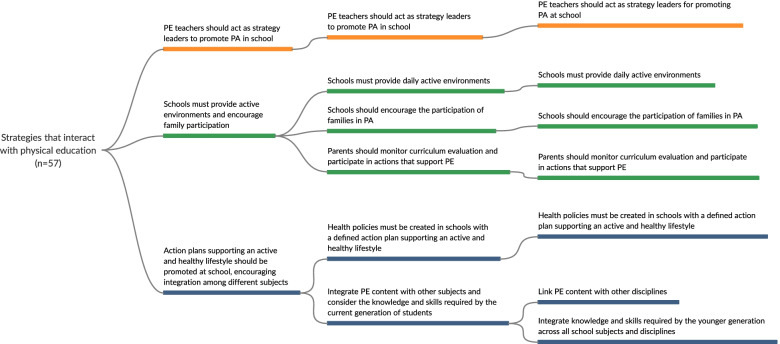
Mapping of recommended strategies that interact with physical education to promote active and healthy lifestyles

After the charting process, the same reviewers summarized the recommendations related to PE aimed at promoting a physically active and healthy lifestyle for students. The following elements were considered: the action carried out (represented by the verb in the infinitive form), the recommended strategy (the object of PE or its contribution to health), the context (targeted audience), and the PE dimensions involved (policy and environment, curriculum, appropriate instruction, assessment, and other practices related to PE). After the recommendations were compiled into the five PE dimensions, a further analysis was performed to regroup recommendations that shared similarities (e.g., 11 recommendations addressed the need to provide and support PE classes with the same rigor as other subjects). This process was discussed by four reviewers (AB, FR, and VBF or KSS) through three consensus meetings (on saturation). Lastly, another two meetings took place to establish a more comprehensive framework for the recommended strategies to promote physical activity and health in PE in each domain (Figs. 2, 3, 4, 5, and 6).

## Results

### Selection of sources of evidence

The electronic search of databases (2,312 titles) and institutional websites (96 titles) identified 2,408 potentially eligible documents, which was reduced to 1,638 after excluding duplicates. In the process of selecting titles and abstracts, 149 documents presented recommendations on strategies for PE. Of these, 86 were excluded because they were published before 2000 (*n* = 32), 20 were ineligible (e.g., abstracts), 20 did not present specific recommendations for PE, eight were documents with a local/regional scope, and six duplicated content from other documents. Therefore, 63 documents (47 and 16 from institutional websites and databases, respectively) were included in this review.

### Characteristics of sources of evidence

Most of the documents reviewed were published from 2011 to 2015 (34.9%) and from 2016 to 2020 (36.5%). Most were practical guides (42.8%), and most addressed PE classes within a broader scope, such as total physical activity, sport, and/or education (i.e., PE was not a primary focus in 65.1% of documents). Regarding the geographic scope of the institutions involved, 27 (47.8%) were national government institutions, and 18 (28.6%) were international or regional (e.g., Europe) institutions. In general, the documents presented recommendations aimed at more than one target audience, focusing mainly on school managers and politicians, and teachers. Strategies were mainly focused on the dimensions of policy and environment (81.0%) and appropriate instruction (66.7%). Detailed information for each document can be found in Tables 2 and 3.

**Table 2:** Publications and methodological characteristics of included documents (*n* = 63)

Publication/methodological characteristics	*n* (% of 63 studies)
Year	
2000–2005	6 (9.5)
2006–2010	12 (19.0)
2011–2015	22 (34.9)
2016–2020	23 (36.5)
Geographic scope of the institution/organization	
Governmental, national level	27 (47.8)
Institutional, national level	18 (28.6)
Institutional, international/regional level	13 (20.6)
Institutional, international/global level	5 (7.9)
Type of document	
Practice guide	27 (42.8)
Institutional statement	19 (30.1)
Institutional recommendations	10 (15.9)
Institutional scientific report	7 (11.1)
Is PE primary in the document?	
Yes	22 (34.9)
No	41 (65.1)
Target population^a^	
Students	20 (31.7)
Teachers	47 (74.6)
School managers and politicians	62 (98.4)
Parents/relatives	28 (44.4)
Recommendation dimensions^a^	
Policy and environment	51 (81.0)
Curriculum	29 (46.0)
Appropriate instruction	42 (66.7)
Student assessment	18 (28.6)

^a^Some documents provided recommendations for more than one target population and dimension

**Table 3:** Methodological information, target population, and recommendation dimensions for each included document (*n* = 63)

Methodological information					Target population				Recommended dimensions			
**Institution/organization**	**Geographic scope of the institution/organization**	**Year**	**Type of document**	**Is PE primary in the document?**	**Students**	**Teachers**	**School managers and politicians**	**Parents/relatives**	**Policy and environment**	**Curriculum**	**Appropriate instruction**	**Student assessment**
National Association of State Boards of Education [21]	Institutional, national level	2000	Practice guide	No	X	X	X	X	X	X	X	X
Fédération Internationale Físico de D ´éducation Phisique[22]	Institutional, International/regional level	2000	Institutional statement	Yes		X	X	X	X	X	X	
CDC [23]	Governmental, national level	2001	Institutional scientific report	No		X	X	X	X		X	
President’s Council on Physical Fitness and Sports [24]	Institutional, International/regional level	2001	Institutional statement	Yes			X		X			
CDC [25]	Governmental, national level	2003	Practice guide	No		X	X			X		
American Heart Association [26]	Governmental, national level	2004	Institutional scientific report	No	X	X	X		X		X	
American Academy of Pediatrics [27]	Governmental, national level	2006	Institutional statement	No		X	X	X	X	X	X	
American Heart Association [28]	Institutional, national level	2006	Institutional statement	No		X	X				X	
CDC [29]	Governmental, national level	2008	Institutional recommendations	No	X	X	X		X	X	X	X
NASPE [30]	Institutional, national level	2008	Institutional statement	No		X	X	X	X	X	X	X
American Heart Association [31]	Institutional, national level	2008	Institutional statement	No		X	X	X	X		X	
Physical and Health Education – Canada [32]	Institutional, national level	2009	Institutional statement	Yes		X	X		X	X	X	
Ministerio de la Protección Social – Colombia[33]	Governmental, national level	2009	Practice guide	No			X	X	X			
SHAPE America [34]	Governmental, national level	2009	Practice guide	Yes	X	X	X		X	X	X	X
Ministry of Social Affairs and Health – Finland [35]	Governmental, national level	2010	Practice guide	No	X	X	X		X	X	X	
ICSSPE [36]	Institutional, International/regional level	2010	Institutional statement	Yes			X		X			
SHAPE America [37]	Governmental, national level	2010	Practice guide	Yes	X	X	X	X	X	X	X	X
CDC [38]	Governmental, national level	2010	Institutional scientific report	Yes		X	X		X	X	X	
Global Forum for PE Pedagogy (GoFPEP) [39]	Institutional, International/regional level	2011	Institutional statement	Yes		X	X	X		X	X	
Ministerio de Salud y Ministerio de Deporte y Recreación – Costa Rica [40]	Governmental, national level	2011	Practice guide	No			X		X	X		
Ministerio de Salud Pública – Ecuador [41]	Governmental, national level	2011	Practice guide	No	X	X	X	X	X		X	
CDC [42]	Institutional, national level	2011	Institutional recommendations	No	X	X	X	X	X	X	X	X
Ministerio de Salud Pública y Bienestar Social – Costa Rica [43]	Governmental, national level	2012	Practice guide	No	X	X	X	X		X	X	
ParticipACTION Advisory Groups [44]	Institutional, national level	2012	Practice guide	No	X	X	X		X	X		
CDC [45]	Governmental, national level	2012	Practice guide	No		X	X	X				
ICSSPE [46]	Institutional, International/regional level	2012	Institutional recommendations	Yes	X	X	X	X	X	X	X	
SHAPE America [47]	Institutional, national level	2012	Practice guide	Yes		X	X				X	
CDC [48]	Governmental, national level	2012	Practice guide	No		X	X	X				
Ministerio de Salud de la Nación – Argentina [49]	Governmental, national level	2013	Institutional recommendations	No			X		X			
The Commonwealth [50]	Institutional, International/regional level	2013	Practice guide	No			X		X	X	X	
UNESCO [51]	Institutional, international/global level	2013	Institutional statement	No			X		X		X	
Federal Council of Physical Education – Brazil [52]	Institutional, national level	2014	Practice guide	Yes	X	X	X	X	X	X	X	X
UK Strength and Conditioning Association on youth resistance training [53]	Institutional, International/regional level	2014	Institutional statement	No		X	X		X		X	
Department for Physical Activity in Public Health Institute for Movement and Neurosciences German Sport University Cologne Am Sportpark Müngersdorf, 50,933 Cologne (Germany) [54]	Institutional, national level	2014	Institutional statement	No		X	X	X			X	
Society of Behavioral Medicine [55]	Institutional, international/global level	2014	Institutional statement	Yes		X	X	X	X		X	
SHAPE America [12]	Governmental, national level	2015	Institutional statement	Yes	X	X	X		X	X	X	X
UNESCO [56]	Institutional, international/global level	2015	Institutional statement	No			X		X		X	
UNESCO [11]	Institutional, international/global level	2015	Institutional statement	Yes			X		X	X	X	X
The Commonwealth [57]	Institutional, International/regional level	2015	Institutional statement	No	X	X	X	X	X	X		X
American Heart Association [58]	Governmental, national level	2015	Institutional scientific report	Yes	X	X	X		X		X	X
Ministerio del Deporte – Chile [59]	Governmental, national level	2016	Practice guide	No		X	X	X	X		X	
Federal Ministry of Health – Germany [60]	Governmental, national level	2016	Practice guide	No		X	X		X		X	
SHAPE America [61]	Governmental, national level	2016	Practice guide	Yes			X		X			
SHAPE America [62]	Governmental, national level	2016	Institutional scientific report	Yes	X	X	X	X	X	X	X	X
Department of Health – Ireland [63]	Governmental, national level	2016	Institutional recommendations	No		X	X		X			
National Physical Activity Plan – United States [64]	Institutional, national level	2016	Practice guide	No		X	X	X	X		X	X
ACHPER [65]	Governmental, national level	2017	Institutional statement									
The Commonwealth [66]	Institutional, International/regional level	2017	Institutional scientific report	No			X		X	X	X	X
CDC [67]	Institutional, national level	2017	Practice guide	No		X	X		X	X		
CDC [68]	Institutional, national level	2017	Practice guide	No	X	X	X		X			
SHAPE America [69]	Governmental, national level	2018	Institutional statement	Yes			X		X			
World Health Organization [70]	Institutional, International/regional level	2018	Institutional recommendations	No			X		X			
European Physical Education Association [71]	Institutional, International/regional level	2018	Institutional recommendations	Yes	X	X	X	X	X	X	X	X
Department of Health and Human Services – United States [72]	Institutional, national level	2018	Practice guide	No	X	X	X	X	X	X	X	
Department of Health – Australia [73]	Governmental, national level	2019	Institutional recommendations	No		X	X		X		X	X
CDC [74]	Governmental, national level	2019	Practice guide	No		X	X	X				
Organisation for Economic Co-operation and Development (OECD) [10]	Institutional, international/global level	2019	Institutional scientific report	Yes			X					
Association for Physical Education (afPE)[75]	Institutional, national level	2019	Practice guide	Yes		X	X		X			
SHAPE America[76]	Institutional, national level	2019	Practice guide	Yes		X	X	X	X		X	X
The Commonwealth[77]	Institutional, International/regional level	2019	Practice guide	No		X	X		X		X	
The Commonwealth[78]	Institutional, International/regional level	2019	Practice guide	No								
ASCD[79]	Institutional, national level	2020	Institutional recommendations	No	X	X	X	X	X	X	X	X
Association for Physical Education – United Kingdom [80]	Institutional, national level	2020	Institutional recommendations	Yes		X	X	X	X	X	X	

*PE* physical education, *CDC* Centers for Disease Control and Prevention, *AHA*, *NASPE* National Association for Sport and Physical Education, *SHAPE* Society of Health and Physical Education, *ICSSPE* International Council of Sport Science and Physical Education, *UNESCO* United Nations Educational, Scientific and Cultural Organization, *ACHPER* Australian Council for Health, Physical Education, and Recreation

### Synthesis of results

In the initial extraction phase, the following strategies were identified: 112 for policy and environment, 106 for appropriate instruction, 57 that involved interactions with school PE, 40 for curriculum, and 38 for student assessment. After three meetings with experts, strategies were grouped into dimensions and those that addressed more than one thematic in the same dimension (e.g., policy and environment) were repeated. For example, in the first meeting with experts, the extracted strategies were grouped into 24 (policy and environment), 12 (curriculum), 16 (appropriate instruction), 14 (student assessment), and 7 (strategies that interacted with PE) recommendations. In the final meeting, 6 recommended strategies were obtained for policy and environment, 5 for curriculum, 4 for appropriate instruction, 3 for student assessment, and 3 for those that involved interactions with PE. The detailed strategies for each stage are shown in Figs. 2, 3, 4, 5, and 6, and the references for each strategy can be consulted in supplementary material 7.

#### Policy and environment

Multiple strategies of policy and environment dimension were merged into six recommendations that addressed the following themes (Fig. 2 and supplementary material 7): i) valuing PE, financing, and better working conditions (*n* = 40 strategies); ii) higher frequency and duration of PE classes and physical activity at school (*n* = 61 strategies); iii) the need for PE classes to be inclusive and never punitive (*n* = 11 strategies); iv) mandatory daily PE at all levels of education to promote student health and well-being (*n* = 15 strategies); v) the evaluation of the implementation of PE guidelines at school (*n* = 6 strategies); and vi) certified, trained, and qualified teachers (*n* = 16 strategies).

#### Curriculum

Five recommendations were identified for the curriculum dimension (Fig. 3 and supplementary material 7): i) the need for the curriculum to follow national, state, and municipal standards with a sequential and progressive structure (*n* = 11 strategies); ii) the need for the inclusion of movement behavior content that promotes physical, cognitive, and social literacy throughout life (*n* = 14 strategies); iii) the need for the inclusion of cross-cutting themes for PE (*n* = 7 strategies); iv) the need for the inclusion of components that improve classes and school community participation (*n* = 8 strategies); and v) the need for the inclusion of content beyond sports (*n* = 7 strategies).

#### Appropriate instruction

For the appropriate instruction dimension, the following recommendations were identified (Fig. 4): i) the need to use strategies to promote physical activity and develop students’ physical literacy, fitness, and social aspects including mental and emotional well-being (*n* = 39 strategies); ii) the need to use strategies that discuss important social issues and include all students (*n* = 43 strategies); iii) the need to use innovative approaches, technologies, and well-maintained equipment and materials (*n* = 8 strategies); and iv) the need to organize the teaching–learning process systematically and present it clearly to the school community (*n* = 13 strategies).

#### Student assessment

The student assessment dimension included three recommendations (Fig. 5): i) the need to understand human movement concepts and achieving an integral development (*n* = 8 strategies); ii) the need to follow identical requirement levels of other disciplines, with evaluations consistent with policy contents and standards (*n* = 23 strategies); and iii) the need to diversify assessment methods to develop the active and healthy lifestyles of students and present their progress to the guardians (*n* = 8 strategies).

#### Strategies that interacted with PE

Three recommendations identified for the dimension of strategies that interacted with PE (Fig. 6) included the following: i) PE teachers should act as strategy leaders to promote physical activity in school (*n* = 10 strategies); ii) schools must provide physically active environments and encourage family participation (*n* = 17 strategies); and iii) action plans supporting an active and healthy lifestyle should be promoted at school, encouraging integration among different subjects (*n* = 17 strategies).

## Discussion

This systematic scoping review sought to summarize worldwide recommendations regarding strategies for PE classes that focus on promoting active and healthy lifestyles among children and adolescents. Overall, we summarized the contents of documents into 21 recommendations for PE classes, with 11 focusing on the dimensions of policy and environment and appropriate instruction. Furthermore, we observed that the reviewed publications did not address PE classes as their primary focus. They were also prepared by government and non-government institutions, mainly directed to managers and teachers.

We found that the most identified strategies addressed the policy and environment dimension. Most of them were related to the recommendations for increasing the frequency and duration of PE classes and physical activity at school. It also recognized the importance of PE, financing, and providing better working conditions. National government institutions have suggested that PE classes should have as much time as possible and be held more times a week [12, 61]. For example, the Society of Health and Physical Educators (SHAPE America) recommends that elementary and middle schools should provide PE at least 150 min/week and 225 min/week, respectively [12, 61]. Moreover, recent systematic reviews have observed that the quantity of PE classes (lessons per week) is associated with improved health outcomes [2] and academic performance [38]. However, additional well-designed studies are needed to clarify the relationship between physical activity and cognitive outcomes, including academic performance [81]. Despite this evidence, a global survey on PE observed that of the 232 countries/autonomous regions analyzed, 97% presented legal requirements for PE classes, but only 71% of countries adhered to implementation in accordance with legal/mandatory obligations or expectations [9]. Hence, PE should be valued as a fundamental part of education and health policies since it contributes to students' health, quality of life, and social development [7, 33, 35, 36, 40, 69], as supported by the World Health Organization [7]. Although the inclusion of specialized PE teachers in primary and secondary schools is quite different among the countries (e.g., some countries only have PE teachers for secondary schools), our results also highlighted the role of PE teachers in improving the teaching–learning process and, consequently, the importance of including trained, certified, and qualified professionals at schools [36, 46, 50, 79].

The main findings of this study regarding appropriate instruction were the need to use strategies to promote physical activity and physical literacy, i.e., motor, cognitive, emotional, and social competences; and the need for using strategies that discuss important social issues and include all students. It is recommended that teachers should consider enjoyable practices to keep students active, physically and as active learners [11, 21, 29, 34, 36, 41, 51, 52, 56, 60, 65, 78]. A favorable environment for children and adolescents' social, mental, and emotional development should also be provided. In this way, PE through diverse classes can serve as a good tool for developing students’ confidence and autonomy, complementing the practice of physical activities outside school, and contributing to maintaining this behavior throughout life [11, 21, 29, 34, 36, 41, 51, 52, 56, 65, 78]. For this purpose, teachers may consider using innovative technologies and approaches that may contribute to realizing these enjoyable practices [34, 37, 52, 82]. Equipment and materials used in classes also need to be diverse [22, 34, 37, 83], although the lack of adequate equipment and materials is a frequent problem reported in schools in low- and middle-income countries.

Our findings support the importance of national, state, and municipal standards for PE classes, considering a sequential and progressive PE curriculum. Diverse content movement behaviors should be included to promote physical literacy throughout life. Although the importance of following academic standards as established by responsible educational institutions is clear [21, 30, 37, 62, 84], it is common to find PE curricula with not well-established structures without a sequence of content and adequate progression [9]. This may be related to the historical panorama of PE, a discipline that has previously played the main role in improving students' physical fitness and sports performance. This scenario has evolved over the years to meet the needs of the school environment. Currently, in addition to sports, the importance of including content that promotes physical, cognitive, and social competencies has been highlighted to help students with demands that arise in their lives [11, 21, 34, 35, 37, 44, 78, 85]. An example is the inclusion of cross-cutting themes of PE to prevent injuries, bullying, and violence at school [10, 25, 65, 66].

According to our findings, recommendations regarding student assessment have highlighted that PE classes have to follow identical requirement levels of other disciplines, with relationship regarding content taught in the classroom and assessment consistent with such content. However, progress must be assessed individually to achieve positive self-concept development without criticism from others [34]. In this sense, monitoring this progress may be improved using different evaluation techniques, such as the inclusion of formative and summative assessments as well as the involvement of guardians to accompany the student's progress [11, 34, 37, 62, 84].

In accordance with United Kingdom’s Association for Physical Education, any evaluation form in the PE curriculum should be part of a meaningful, planned, and progressive program based on integral student concept [80], that is, the form of assessment should not focus only on physical fitness components. Also, several national institutions have recommended that, through PE classes, students should be able to understand concepts related to human movement and progress in the development of their motor, cognitive, and social skills [21, 29, 34, 37, 52]. Therefore, adjusting student assessments may contribute to PE teaching–learning process.

The main findings regarding the strategies that interact with PE address the need for action plans that support an active and healthy lifestyle to be promoted at school, encouraging integration among the different actors of the school context. Health policies at school are essential since a well-defined action plan can help adolescents adopt more active and healthy lifestyles. For instance, systematic reviews have shown that strategies that involve PE-related strategies combined with other actions (e.g., provision of environments for physical activity practice, school community training, and educational actions) are effective for promoting active and healthy lifestyles among children and adolescents [13]. In this context, each school community member has an important role in developing active lifestyles at school, and they should be supported by PE teachers, as leaders and school actors for providing supportive environments. This may help to provide daily active environments, as well as actions that encourage families and the community to participate in physical activity practices [34, 41, 44, 45, 48, 52, 59, 62, 74, 82, 84].

Lastly, our findings indicate that most publications did not have PE as their primary focus and had managers and teachers as their main target audience. This may be explained by the presence of a few institutions explicitly directed to the PE discipline, such as SHAPE America [84] and the European Physical Education Association [71]. In general, most of the institutions included in the reviewed documents presented global recommendations for physical activity and health, with most documents having certain sections related to PE classes. Moreover, managers and teachers have been the primary target audience of such documents because most of their recommendations are related to the dimensions of policy and environment, as well as appropriate instruction.

Based on our findings, the below implications can be considered for future research. Studies should focus on evaluating which strategies could be considered reliable, feasible, and sustainable when implemented on a large scale, considering the different actors involved in the schools and PE classes. For building bridges between theory and practice [86], studies should assess the types of barriers and facilitators in implementing such PE strategies, taking into account local, regional, national, and international contexts (e.g., the contextual difference between primary and secondary grades). Finally, there is a need to evaluate the long-term effectiveness of recommended PE strategies (as primary intervention strategies) to improve healthy lifestyle indicators among the population. For policy and practice, our summary of 21 recommendations for PE classes for healthy lifestyles may aid stakeholders and policymakers when proposing PE classes for local, regional, and national policies and practices.

Some key strengths of this study should be noted. First, this scoping review has produced meaningful findings that provide a mapping of strategies that have been recommended for PE, helping to understand which types of strategies should be adopted in relation to each studied dimension. Second, we conducted this review following a rigorous methodology, in line with guidance for systematic scoping reviews [17]. Finally, we encompassed a range of documents from around the world, including documents from key institutions which prioritize the PE subject, allowing us to provide a more comprehensive overview of the current situation.

This review also has some limitations that should be acknowledged. A scoping review differs from other types of systematic reviews in that it does not aim to evaluate the quality of available evidence and does not present the same rigorous systematic process as other types of reviews. Nevertheless, a systematic scoping review aims to identify the main concepts related to a specific research area, which may allow one to identify findings that would not otherwise have been identified using traditional systematic review methods. Therefore, the studies included in this review were not evaluated in terms of their methodological rigor, and we did not intend to determine which strategies are effective in promoting an active and healthy lifestyle. Moreover, although we developed a wide encompassing search strategy, it is possible that some relevant studies could not be included, for example, documents and research that were not published in journals of the searched databases. However, it is important to highlight that these characteristics are common in systematic scoping reviews. Finally, we were unable to consider the different characteristics between primary and secondary school PE strategies.

## Conclusions

We presented a mapping of evidence showing 21 potential strategies for PE classes focused on active and healthy lifestyles. Such strategies are linked to five dimensions aimed at different actors in the school community. Over half of the strategies are linked to the dimensions of policy and environment and appropriate instruction. Also, a policy with PE as a priority area is recommended, one in which PE is mandatory and valued at all educational levels, with a weekly frequency that contributes to an active and healthy lifestyle. Our findings also show the importance of guaranteeing experiences of human movement contents throughout the curriculum promoting physical, cognitive, and social literacy. This is important during the lifespan, respecting the needs, preferences, and capacities of students according to their school levels. Other aspects addressed were the inclusion of a curriculum that follows standards with a sequential and progressive structure, adequate student assessments, and action plans supporting an active and healthy lifestyle at school.

## Supplementary Information

**Additional file 1. **

**Additional file 2. **

**Additional file 3. **

**Additional file 4. **

**Additional file 5. **

**Additional file 6. **

**Additional file 7. **

## Data Availability

The summary of reviewed articles is available in Tables, Figures and Additional files.

## Abbreviation

PE: Physical education

## References

[1] National Human Development Report. Movement is Life: Sports and Physical Activities for Everyone. 2017.

[2] García-Hermoso A, Alonso-Martínez AM, Ramírez-Vélez R, Pérez-Sousa MÁ, Ramírez-Campillo R, Izquierdo M (2020). Association of Physical Education With Improvement of Health-Related Physical Fitness Outcomes and Fundamental Motor Skills Among Youths: A Systematic Review and Meta-analysis. JAMA Pediatr.

[3] White RL, Babic MJ, Parker PD, Lubans DR, Astell-Burt T, Lonsdale C (2017). Domain-Specific Physical Activity and Mental Health: A Meta-analysis. Am J Prev Med.

[4] Opstoel K, Chapelle L, Prins FJ, De Meester A, Haerens L, van Tartwijk J (2020). Personal and social development in physical education and sports A review study. European Physical Education Review SAGE Publications Ltd.

[5] Barnett LM, Dudley DA, Telford RD, Lubans DR, Bryant AS, Roberts WM (2019). Guidelines for the Selection of Physical Literacy Measures in Physical Education in Australia. J Teach Phys Educ.

[6] Dias JRA, Souza A dos S, Oliveira RC de. Physical Education and health: perspectives for high school. Physis [Internet]. IMS-UERJ; 2020 [cited 2021 Nov 23];30. Available from: http://www.scielo.br/j/physis/a/yckyGD56ph88FSwyNQN4T3P/?lang=en

[7] World Health Organization. Global action plan on physical activity 2018–2030: more active people for a healthier world [Internet]. World Health Organization; 2018 [cited 2020 Jun 1]. Available from: http://www.who.int/ncds/prevention/physical-activity/global-action-plan-2018-2030/en/

[8] Uddin R, Salmon J, Islam SMS, Khan A (2020). Physical education class participation is associated with physical activity among adolescents in 65 countries. Scientific Reports Nature Publishing Group.

[9] UNESCO. World-wide survey of school physical education. UNESCO Publishing; 2014.

[10] Organisation for Economic Co-operation and Development (OECD). Making Physical Education Dynamic and Inclusive for 2030: International Curriculum Analysis [Internet]. 2019 [cited 2020 Jul 5]. Available from: https://www.oecd.org/education/2030-project/contact/OECD_FUTURE_OF_EDUCATION_2030_MAKING_PHYSICAL_DYNAMIC_AND_INCLUSIVE_FOR_2030.pdf

[11] UNESCO. Diretrizes em educação física de qualidade (EFQ) para gestores de políticas [Internet]. 2015 [cited 2020 Jul 5]. Available from: https://unesdoc.unesco.org/ark:/48223/pf0000231963

[12] SHAPE America. The Essential Components of Physical Education [Internet]. 2015 [cited 2020 Jul 5]. Available from: https://www.shapeamerica.org/upload/TheEssentialComponentsOfPhysicalEducation.pdf

[13] Langford R, Bonell CP, Jones HE, Pouliou T, Murphy SM, Waters E, et al. The WHO Health Promoting School framework for improving the health and well-being of students and their academic achievement. Cochrane Developmental, Psychosocial and Learning Problems Group, editor. Cochrane Database of Systematic Reviews [Internet]. 2014 [cited 2018 Oct 26]; Available from: http://doi.wiley.com/10.1002/14651858.CD008958.pub2 10.1002/14651858.CD008958.pub2 PMC11214127 24737131

[14] Ramirez Varela A, Salvo D, Pratt M, Milton K, Siefken K, Bauman A (2018). Worldwide use of the first set of physical activity Country Cards: The Global Observatory for Physical Activity - GoPA!. Int J Behav Nutr Phys Act.

[15] Klepac Pogrmilovic B, Ramirez Varela A, Pratt M, Milton K, Bauman A, Biddle SJH (2020). National physical activity and sedentary behaviour policies in 76 countries: availability, comprehensiveness, implementation, and effectiveness. Int J Behav Nutr Phys Act.

[16] Beni S, Fletcher T, Chróinín DN (2017). Meaningful Experiences in Physical Education and Youth Sport: A Review of the Literature. Quest Routledge.

[17] Tricco AC, Lillie E, Zarin W, O’Brien KK, Colquhoun H, Levac D (2018). PRISMA Extension for Scoping Reviews (PRISMA-ScR): Checklist and Explanation. Ann Intern Med.

[18] Munn Z, Peters MDJ, Stern C, Tufanaru C, McArthur A, Aromataris E (2018). Systematic review or scoping review? Guidance for authors when choosing between a systematic or scoping review approach. BMC Med Res Methodol.

[19] Brasil. Base Nacional Comum Curricular [Internet]. Ministério da Educação; 2018 [cited 2020 Oct 9]. Available from: http://basenacionalcomum.mec.gov.br/images/BNCC_EI_EF_110518_versaofinal_site.pdf

[20] McGowan J, Sampson M, Salzwedel DM, Cogo E, Foerster V, Lefebvre C (2016). PRESS Peer Review of Electronic Search Strategies: 2015 Guideline Statement. J Clin Epidemiol.

[21] Bogden JF. Fit, Healthy, and Ready To Learn: A School Health Policy Guide. Part I: Physical Activity, Health Eating, and Tobacco-Use Prevention. National Association of State Boards of Education, 277 South Washington Street, Suite 100, Alexandria, VA 22314 ($22); 2000;1–230.

[22] Federation Internationale d´Éducation Physique. The World Manifest of Physical Education FIEP 2000 [Internet]. 2000 [cited 2020 Jul 5]. Available from: http://fiepeurope.eu/manifest.php

[23] Task Force on Community Preventive Services (2001). Increasing physical activity A report on recommendations of the Task Force on Community Preventive Services. MMWR Recommendations and reports : Morbidity and mortality weekly report Recommendations and reports.

[24] Haney L (2001). Physical education statement by the President’s Council on Physical Fitness and Sports. NASNewsletter.

[25] Barrios LC, Sleet DA, Mercy JA. CDC School Health Guidelines to Prevent Unintentional Injuries and Violence. American Journal of Health Education [Internet]. American Alliance for Health, Physical Education, Recreation and Dance; 2003 [cited 2020 Jun 17];34. Available from: https://eric.ed.gov/?id=EJ853628

[26] Hayman LL, Williams CL, Daniels SR, Steinberger J, Paridon S, Dennison BA (2004). American Heart Association. Circulation.

[27] American Academy of Pediatrics (2006). Active healthy living: prevention of childhood obesity through increased physical activity. Pediatrics.

[28] Pate RR, Davis MG, Robinson TN, Stone EJ, McKenzie TL, Young JC (2006). Promoting physical activity in children and youth: A leadership role for schools - A scientific statement from the American Heart Association Council on Nutrition, Physical Activity, and Metabolism (Physical Activity Committee) in collaboration with the Councils on Cardiovascular Disease in the Young and Cardiovascular Nursing. Circulation.

[29] Centers for Disease Control and Prevention (2008). A CDC Review of School Laws and Policies Concerning Child and Adolescent Health. J Sch Health.

[30] National Association for Sport and Physical Education (2008). A Position Statement from the National Association for Sport and Physical Education: Comprehensive School Physical Activity Program. Strategies A Journal for Physical and Sport Educators..

[31] Pate RR, O’Neill JR (2008). Summary of the American Heart Association scientific statement: promoting physical activity in children and youth: a leadership role for schools. The Journal of cardiovascular nursing.

[32] Mandigo J, Francis PDN, Lodewyk EDK. Position Paper Physical Literacy for Educators. 2009;13.

[33] Ministerio de la Protección Social. Guía para el desarrollo de programas intersectoriales y comunitarios para la promoción de la actividad física: programa nacional de actividad física Colombia activa y saludable [Internet]. Imprenta Nacional de Colombia; 2009. Available from: https://books.google.com.br/books?id=MWiAswEACAAJ

[34] SHAPE America. Appropriate Instructional Practice Guidelines, K-12: A Side-by-Side Comparison SHAPE America – Society of Health and Physical Educators [Internet]. 2009 [cited 2020 Jul 5]. Available from: https://www.shapeamerica.org/upload/Appropriate-Instructional-Practice-Guidelines-K-12.pdf

[35] Ministry of Social Affairs and Health. Recommendations for the promotion of physical activity in Finland. 2010.

[36] International Council of Sport Science and Physical EducationScience Education Policy. International Position Statement on Physical Education. 2010.

[37] SHAPE America. Opportunity to Learn: Guidelines for Elementary, Middle & High School Physical Education - A Side‐by‐Side Comparison [Internet]. 2010 [cited 2020 Jul 5]. Available from: https://www.shapeamerica.org/standards/guidelines/upload/Opportunity-to-Learn-Grid.pdf

[38] Centers for Disease Control and Prevention. The Association Between School-Based Physical Activity, Including Physical Education, and Academic Performance. 2010.

[39] Edginton CR, Chin MK, Geadelmann PL, Ahrabi-Fard I (2011). Global Forum for Physical Education Pedagogy 2010 (GoFPEP 2010): Health and Physical Education Pedagogy in the 21st Century - A Statement of Consensus. Int J Phys Educ.

[40] Costa Rica. Ministerio de Salud y Ministerio de Deporte y Recreación. Plan Nacional de Actividad Física y Salud 2011–2021. 2011.

[41] Ecuador. Ministerio de salud pública Del Ecuador, Coordinación Nacional de Nutrición. Guia de Actividad Física dirigida al personal de salud II. 2011.

[42] Centers for Disease Control and Prevention. School Health Guidelines to Promote Healthy Eating and Physical Activity: Executive Summary. Centers for Disease Control and Prevention, 1600 Clifton Road, Atlanta, GA 30333;2011;1–8

[43] Ministerio de Salud Pública y Bienestar Social (2012). Directrices de Evaluación de Niñas, Niños y Adolescentes para la Actividad Física Pedagógica Recreativa y Deportiva Escolar en Paraguay. Pediatr (Asunción).

[44] ParticipACTION Advisory Groups. Active Canada 20/20: A physical activity strategy and change agenda for Canada. 2012.

[45] Centers for Disease Control and Prevention. Parent Engagement: Strategies for Involving Parents in School Health. 2012.

[46] International Council of Sport Science and Physical EducationScience Education Policy. International Benchmarks for Physical Education Systems Developed by ICSSPE’s International Committee of Sport Pedagogy. 2012.

[47] SHAPE America. Instructional Framework for Fitness Education In Physical Education [Internet]. 2012 [cited 2020 Jul 5]. Available from: https://www.shapeamerica.org/standards/guidelines/upload/Instructional-Framework-for-Fitness-Education-in-Physical-Education.pdf

[48] Centers for Disease Control and Prevention. Promoting Parent Engagement: Improving Student Health and Academic Achievement. 2012.

[49] Incarbone O, Ferrante D, Bazan N, Gonzalez G, Barengo N, Kanfino J. Manual Director de actividad física y salud de la república Argentina. Plan Nacional Argentina Saludable Dirección de Promoción de la Salud y Control de Enfermedades No Transmisibles Ministerio de Salud de la Nación. 2013;8.

[50] Kay T, Dudfield O, Commonwealth Secretariat. The commonwealth guide to advancing development through sport. London: Commonwealth Secretariat; 2013.

[51] UNESCO. Declaration of Berlin: International Conference of Ministers and Senior Officials Responsible for Physical Education and Sport [Internet]. 2013 [cited 2020 Jul 5]. Available from: https://unesdoc.unesco.org/ark:/48223/pf0000221114

[52] OLIVEIRA ARC de, SARTORI SK, LAURINDO E. Recomendações para a Educação Física escolar. Sistema CONFEF/CREFs Conselhos Federal e Regionais de Educação Física. 2014;

[53] Lloyd RS, Faigenbaum AD, Stone MH, Oliver JL, Jeffreys I, Moody JA (2014). Position statement on youth resistance training: the 2014 International Consensus. Br J Sports Med.

[54] Graf C, Beneke R, Bloch W, Bucksch J, Dordel S, Eiser S (2014). Recommendations for promoting physical activity for children and adolescents in Germany. A consensus statement Obesity Facts.

[55] Buscemi J, Kong A, Fitzgibbon ML, Bustamante EE, Davis CL, Pate RR (2014). Society of Behavioral Medicine position statement: elementary school-based physical activity supports academic achievement. Translational behavioral medicine.

[56] UNESCO. International Charter of Physical Education, Physical Activity and Sport [Internet]. 2015 [cited 2020 Jul 5]. Available from: https://unesdoc.unesco.org/ark:/48223/pf0000235409

[57] The common Wealth. Sport for Development and Peace and the 2030 Agenda for Sustainable Development [Internet]. 2015 [cited 2020 Jul 5]. Available from: https://thecommonwealth.org/sites/default/files/inline/CW_SDP_2030%2BAgenda.pdf

[58] American Heart Association. Increasing and Improving Physical Education and Physical Activity in Schools: Benefits for Children’s Health and Educational Outcomes [Internet]. 2015 [cited 2020 Jul 5]. Available from: https://www.heart.org/idc/groups/heart-public/@wcm/@adv/documents/downloadable/ucm_473782.pdf

[59] Ministerio del Deporte, Gobierno de Chile. Política Nacional de Actividad Física y Deporte 2016–2025 [Internet]. 2016 [cited 2020 Jul 2]. Available from: http://www.mindep.cl/wp-content/uploads/2015/05/POLITICA-ULTIMA-VERSI%C3%93N-021116.pdf

[60] Rütten A, Pfeifer K, Banzer W, Ferrari N, Füzéki E, Geidl W, et al. National Recommendations for Physical Activity and Physical Activity Promotion [Internet]. 2016 [cited 2020 Jul 3]. Available from: https://opus4.kobv.de/opus4-fau/frontdoor/index/index/docId/7827

[61] SHAPE America. Guide for the Physical Education Policy [Internet]. 2016 [cited 2020 Jul 5]. Available from: https://www.shapeamerica.org/advocacy/upload/Guide-for-Physical-Education-Policy-9-23-14.pdf

[62] SHAPE America. Shape of the Nation Status of Physical Education in the USA [Internet]. 2016 [cited 2020 Jul 5]. Available from: https://www.shapeamerica.org/advocacy/son/2016/upload/Shape-of-the-Nation-2016_web.pdf

[63] Department of Health (Ireland). Get Ireland Active! The National Physical Activity Plan for Ireland [Internet]. 2016 [cited 2020 Jul 5]. Available from: https://assets.gov.ie/12198/5f3dbab207f2464bba3b9b3f6d02bff6.pdf

[64] National Physical Activity Plan Alliance. National Physical Activity Plan Alliance. U.S. National Physical Activity Plan. 2016 [Internet]. 2016 [cited 2020 Jul 5]. Available from: https://www.physicalactivityplan.org/docs/2016NPAP_Finalforwebsite.pdf

[65] The Australian Council for Health, Physical Education and Recreation (ACHPER). ACHPER NATIONAL POSITION STATEMENT: Support of the Australian Curriculum: Health and Physical Education [Internet]. 2017 [cited 2020 Jul 13]. Available from: https://www.achper.org.au/documents/item/393

[66] The common Wealth. Enhancing the Contribution of Sport to the Sustainable Development Goals [Internet]. OECD Publishing; 2017 [cited 2020 Jul 5]. Available from: http://www.thecommonwealth-ilibrary.org/commonwealth/development/enhancing-the-contribution-of-sport-to-the-sustainable-development-goals_9781848599598-en

[67] Centers for Disease Control and Prevention. School Health Index: A Self-Assessment and Planning Guide Elementary School 2017 [Internet]. 2017 [cited 2020 Jul 5]. Available from: https://www.cdc.gov/healthyschools/shi/pdf/Elementary-Total-2017.pdf

[68] Centers for Disease Control and Prevention. School Health Index: A Self-Assessment and Planning Guide Middle and High School 2017 [Internet]. 2017 [cited 2020 Jul 5]. Available from: https://www.cdc.gov/healthyschools/shi/pdf/Middle-High-Total-2017.pdf

[69] SHAPE America. Physical Education is Essential for All Students: No Substitutions, Waivers or Exemptions for Physical Education. 2018;6.

[70] World Health Organization. Global action plan on physical activity 2018–2030: more active people for a healthier world [Internet]. 2018 [cited 2020 Jul 5]. Available from: https://www.who.int/ncds/prevention/physical-activity/global-action-plan-2018-2030/en/

[71] European Physical Education Association. European Framework of Quality Physical Education [Internet]. 2018 [cited 2020 Jul 5]. Available from: http://www.eupea.com/wp-content/uploads/2018/02/European-Framework-of-Quality-PE.pdf

[72] US Department of Health and Human Services. Physical activity guidelines advisory committee scientific report. Washington (DC): US Department of Health and Human Services. 2018;

[73] Departament of Health of Australia. Australian 24-Hour Movement Guidelines for Children (5–12 years) and Young People (13–17 years): An Integration of Physical Activity, Sedentary Behaviour, and Sleep. 2019. 10.1186/s12966-021-01236-2 PMC8734238 34991606

[74] Centers for Disease Control and Prevention. Parents for Healthy Schools: A Guide for Getting Parents Involved from K–12. 2019;26.

[75] Association for Physical Education (afPE). The Inspection and Maintenance of Gymnastics, Sports Hall, Fixed Play, Fitness and Sports Equipment [Internet]. 2019 [cited 2020 Jul 5]. Available from: https://www.afpe.org.uk/physical-education/wp-content/uploads/Inspection-of-Equipment-of-PESSPA-Web.pdf

[76] SHAPE America. Getting to Know Your Child’s PE Program: A Parent’s Guide [Internet]. 2019 [cited 2020 Jul 5]. Available from: https://www.shapeamerica.org/uploads/pdfs/2017/downloads/eguides/Parent_Checklist.pdf

[77] The common Wealth. Measuring the contribution of sport, physical education and physical activity to the Sustainable Development Goals Toolkit and model indicators [Internet]. 2019 [cited 2020 Jul 5]. Available from: https://thecommonwealth.org/sites/default/files/inline/Sport-SDGs-Indicator-Framework.pdf

[78] The common Wealth. Model indicators on sport, physical education and physical activity and the Sustainable Development Goals [Internet]. 2019 [cited 2020 Jul 5]. Available from: https://thecommonwealth.org/sites/default/files/inline/Sport%20and%20SDG%20Indicators%20v3.1.pdf

[79] Association for Supervision and Curriculum Development (ASCD). ASCD’s Position on the Whole Child [Internet]. 2020 [cited 2020 Jul 5]. Available from: http://files.ascd.org/pdfs/programs/WholeChildNetwork/2020-whole-child-network-learning-compact-renewed.pdf

[80] Association for Physical Education. Health Position Paper afPE 2020 [Internet]. 2020 [cited 2020 Jul 5]. Available from: https://www.afpe.org.uk/physical-education/wp-content/uploads/Health-Position-Paper-2020-Web.pdf

[81] Singh AS, Saliasi E, van den Berg V, Uijtdewilligen L, de Groot RHM, Jolles J (2019). Effects of physical activity interventions on cognitive and academic performance in children and adolescents: a novel combination of a systematic review and recommendations from an expert panel. Br J Sports Med BMJ Publishing Group Ltd and British Association of Sport and Exercise Medicine.

[82] (17) (PDF) Global forum for physical education pedagogy 2010 (GoFPEP 2010) statement of consensus:: pedagogy of health and physical education in the xxith century [Internet]. ResearchGate. [cited 2020 Jun 17]. Available from: https://www.researchgate.net/publication/317462682_Global_forum_for_physical_education_pedagogy_2010_GoFPEP_2010_statement_of_consensus_pedagogy_of_health_and_physical_education_in_the_xxith_century

[83] Directrices de Evaluación de Niñas (2012). Niños y Adolescentes para la Actividad Física Pedagógica Recreativa y Deportiva Escolar en Paraguay. Pediatr (Asunción).

[84] SHAPE America-Society of Health and Physical Educators. The essential components of physical education. Author Reston, VA; 2015.

[85] Council on Sports Medicine and Fitness and Council on School Health (2006). Active healthy living: prevention of childhood obesity through increased physical activity. Pediatrics.

[86] Durlak JA, DuPre EP (2008). Implementation Matters: A Review of Research on the Influence of Implementation on Program Outcomes and the Factors Affecting Implementation. Am J Community Psychol.

