# Supplementary material for: Mapping recommended strategies to promote active and healthy lifestyles through physical education classes: a scoping review
Source: Int J Behav Nutr Phys Act. 2022 Mar 28;19:36. doi: 10.1186/s12966-022-01278-0 (PMC8962044; doi:10.1186/s12966-022-01278-0)
Supplement: Supplementary file 2 — Additional file 2. [file 12966_2022_1278_MOESM2_ESM.doc]

SUPPLEMENTARY MATERIAL 01

List of accessed web-based portals

| **Region** | **Organization** | **Publication Year** | **Title** | **Links to documents** |
| --- | --- | --- | --- | --- |
| International | Fédération Internationale D´Éducation Physique (FIEP) | 2000 | The World Manifest of Physical Education FIEP 2000 | <http://fiepeurope.eu/manifest.php> |
| Canada | Physical and Health Education Canada | 2009 | Position Paper: Physical Literacy for Educators | <https://www.speaonline.ca/uploads/2/2/0/2/22022168/phe_canada_position_paper_physical_literacy_for_educators.pdf> |
| Colombia | Ministerio de la Protección Social | 2009 | Guía para el desarrollo de programas intersectoriales y comunitarios para la promoción de la actividad física: programa nacional de actividad física: Programa Nacional de Actividad Física Colombia Activa y Saludable | [https://www.boyaca.gov.co/SecSalud/images/Documentos/INFORME%20COLOMBIA%20ACTIVA-%20Ultimo.pdf](https://www.boyaca.gov.co/SecSalud/images/Documentos/INFORME COLOMBIA ACTIVA- Ultimo.pdf) |
| USA | Society of Health and Physical Educator (SHAPE America) | 2009 | Appropriate Instructional Practice Guidelines, K-12: A Side-by-Side Comparison: SHAPE America – Society of Health and Physical Educators | <https://www.shapeamerica.org/upload/Appropriate-Instructional-Practice-Guidelines-K-12.pdf> |
| International | International Council of Sport Science and Physical EducationScience Education Policy (ICSSPE) | 2010 | International Position Statement on Physical Education | [https://www.icsspe.org/sites/default/files/International%20Position%20Statement%20on%20Physical%20Education.pdf](https://www.icsspe.org/sites/default/files/International Position Statement on Physical Education.pdf) |
| Finland | Ministry of Social Affairs and Health | 2010 | Recommendations For the Promotion of Physical Activity in Finland | <https://www.nescon.medicina.ufmg.br/biblioteca/imagem/4855.pdf> |
| USA | Department of Health and Human Services Centers for Disease Control and Prevention (CDC) | 2010 | The association between school based physical activity, including physical education, and academic performance. | <https://www.cdc.gov/healthyyouth/health_and_academics/pdf/pa-pe_paper.pdf> |
| USA | Society of Health and Physical Educator (SHAPE America) | 2010 | Opportunity to Learn Guidelinesfor Elementary, Middle & High School Physical Education: A Side‐by‐Side Comparison | <https://www.shapeamerica.org/standards/guidelines/upload/Opportunity-to-Learn-Grid.pdf> |
| Costa Rica | Ministerio de Salud y Ministerio de Deporte y Recreación | 2011 | Plan Nacional de Actividad Física y Salud 2011-2021. | <http://onarhus.ministeriodesalud.go.cr/sites/observatorio_cr/files/documentos/DPEEAS_plan_actividad_fisica_2011.pdf> |
| Equador | Unidad de Nutrición – Ministerio de Salud Pública | 2011 | Guia de Actividad Física dirigida al personal de salud II. | [https://aplicaciones.msp.gob.ec/salud/archivosdigitales/documentosDirecciones/dnn/archivos/GUIA%20DE%20ACTIVIDAD%20FISICA%202.pdf](https://aplicaciones.msp.gob.ec/salud/archivosdigitales/documentosDirecciones/dnn/archivos/GUIA DE ACTIVIDAD FISICA 2.pdf) |
| Canada | ParticipACTION Advisory Groups. | 2012 | ParticipACTION Advisory Groups. Active Canada 20/20: A Physical Activity Strategy and Change Agenda for Canada – Creating a Culture of an Active Nation, May 2012 version. Available at: http://www.activecanada2020.ca | <https://sirc.ca/app/uploads/2019/12/Active-Canada-2020-_-FinalE-May-2012.pdf> |
| USA | Society of Health and Physical Educator (SHAPE America) | 2012 | Instructional Framework for Fitness Education In Physical Education | <https://www.shapeamerica.org/standards/guidelines/upload/Instructional-Framework-for-Fitness-Education-in-Physical-Education.pdf> |
| International | International Council of Sport Science and Physical EducationScience Education Policy (ICSSPE) | 2012 | International Benchmarks for Physical Education Systems Developed by ICSSPE’s International Committee of Sport Pedagogy | [https://www.icsspe.org/system/files/Final%20ICSP%20Benchmarks%20ICSSPE%20Dec%202012.pdf](https://www.icsspe.org/system/files/Final ICSP Benchmarks ICSSPE Dec 2012.pdf) |
| USA | Department of Health and Human Services Centers for Disease Control and Prevention (CDC) | 2012 | Parent Engagement: Strategies for Involving Parents in School Health. | <https://www.cdc.gov/healthyschools/parentsforhealthyschools/pdf/parent_engagement_strategies.pdf> |
| Argentina | Ministerio de Salud de la Nación | 2013 | Manual Director de actividad física y salud de la república Argentina. | [manual-actividad-fisica.pdf (msal.gob.ar)](http://www.msal.gob.ar/images/stories/ministerio/manual-actividad-fisica.pdf) |
| International | United Nations Educational, Scientific and Cultural Organization (UNESCO) | 2013 | Declaration of Berlin. Conference:International Conference of Ministers and Senior Officials Responsible for Physical Education and Sport. | <https://unesdoc.unesco.org/ark:/48223/pf0000221114> |
| International | The common Wealth | 2013 | The Commonwealth Guide to Advancing Development through Sport: Commonwealth Secretariat | <https://thecommonwealth.org/sites/default/files/inline/The%2BCW%2BGuide%2Bto%2BAdvancing%2BSport%2BEB.pdf> |
| Brazil | Conselho Federal de Educação Física (CONFEF) | 2014 | Recomendações para Educação Física Escolar | <https://www.listasconfef.org.br/arquivos/publicacoes/RECOMENDACOES_PARA_A_EDUCACAO_FISICA_ESCOLAR2.pdf> |
| USA | American Heart Association (AHA) | 2015 | Increasing and Improving Physical Education and Physical Activity in Schools: Benefits for Children’s Health and Educational Outcomes | <https://www.heart.org/idc/groups/heart-public/@wcm/@adv/documents/downloadable/ucm_473782.pdf> |
| International | United Nations Educational, Scientific and Cultural Organization (UNESCO) | 2015 | International Charter of Physical Education, Physical Activity and Sport | <https://unesdoc.unesco.org/ark:/48223/pf0000235409> |
| International | United Nations Educational, Scientific and Cultural Organization (UNESCO) | 2015 | Quality physical education (QPE) guidelines for policy-makers. | <https://unesdoc.unesco.org/ark:/48223/pf0000231963> |
| International | The common Wealth | 2015 | Sport for Development and Peace and the 2030 Agenda for Sustainable Development: Commonwealth Secretariat | <https://thecommonwealth.org/sites/default/files/inline/CW_SDP_2030%2BAgenda.pdf> |
| USA | Society of Health and Physical Educator (SHAPE America) | 2015 | The Essential Components of Physical Education | [https://www.shapeamerica.org//upload/TheEssentialComponentsOfPhysicalEducation.pdf](https://www.shapeamerica.org/upload/TheEssentialComponentsOfPhysicalEducation.pdf) |
| Chile | Ministerio del Deporte | 2016 | Ministerio del Deporte, Gobierno de Chile. 2016. «Política Nacional de Actividad Física y Deporte 2016-2025». (1a. ed.), Santiago de Chile. | [http://www.mindep.cl/wp-content/uploads/2015/05/POLITICA-ULTIMA-VERSI%C3%93N-021116.pdf](http://www.mindep.cl/wp-content/uploads/2015/05/POLITICA-ULTIMA-VERSIÓN-021116.pdf) |
| Germany | Federal Ministry of Health | 2016 | National Recommendations for Physical Activity and Physical Activity Promotion | <https://www.sport.fau.de/files/2015/05/National-Recommendations-for-Physical-Activity-and-Physical-Activity-Promotion.pdf> |
| Irlanda | Department of Health (Ireland) | 2016 | Get Ireland Active! National Physical Activity Plan for Ireland | <https://assets.gov.ie/12198/5f3dbab207f2464bba3b9b3f6d02bff6.pdf> |
| USA | National Physical Activity Plan Alliance | 2016 | U.S. National Physical Activity Plan | <https://www.physicalactivityplan.org/docs/2016NPAP_Finalforwebsite.pdf> |
| USA | Society of Health and Physical Educator (SHAPE America) | 2016 | Shape of the Nation Status of Physical Education in the USA | [https://www.shapeamerica.org//advocacy/son/2016/upload/Shape-of-the-Nation-2016_web.pdf](https://www.shapeamerica.org/advocacy/son/2016/upload/Shape-of-the-Nation-2016_web.pdf) |
| USA | Society of Health and Physical Educator (SHAPE America) | 2016 | Guide for the Physical Education Policy | [https://www.shapeamerica.org//advocacy/upload/Guide-for-Physical-Education-Policy-9-23-14.pdf](https://www.shapeamerica.org/advocacy/upload/Guide-for-Physical-Education-Policy-9-23-14.pdf) |
| Australia | The Australian Council for Health, Physical Education and Recreation South Australian Branch Incorporated (ACHPER) | 2017 | ACHPER NATIONAL POSITION STATEMENT: Support of the Australian Curriculum: Health and Physical Education | <https://www.achper.org.au/documents/item/393> |
| USA | Department of Health and Human Services Centers for Disease Control and Prevention (CDC) | 2017 | School Health Index: A Self-Assessment and Planning Guide Elementary School | <https://www.cdc.gov/healthyschools/shi/pdf/Elementary-Total-2017.pdf> |
| USA | Department of Health and Human Services Centers for Disease Control and Prevention (CDC) | 2017 | School Health Index: A Self-Assessment and Planning Guide Middle and High School | <https://www.cdc.gov/healthyschools/shi/pdf/Middle-High-Total-2017.pdf> |
| International | The common Wealth | 2017 | Enhancing the Contribution of Sport to the Sustainable Development Goals | <https://thecommonwealth.org/sites/default/files/inline/EnhancingtheContributionofSporttotheSustainableDevelopmentGoals.pdf> |
| USA | U.S. Department of Health and Human Services | 2018 | 2018 Physical Activity Guidelines Advisory Committee Scientific Report. | <https://health.gov/sites/default/files/2019-09/PAG_Advisory_Committee_Report.pdf> |
| International | European Physical Education Association (EFQPE) | 2018 | European Framework of Quality Physical Education | <http://www.eupea.com/wp-content/uploads/2018/02/European-Framework-of-Quality-PE.pdf> |
| International | World Health Organization (WHO) | 2018 | Global action plan on physical activity 2018–2030: more active people for a healthier world | <https://apps.who.int/iris/bitstream/handle/10665/272722/9789241514187-eng.pdf> |
| USA | Society of Health and Physical Educator (SHAPE America) | 2018 | Physical Education is Essential for All Students: No Substitutions, Waivers or Exemptions for Physical Education | <https://www.shapeamerica.org/uploads/pdfs/2018/advocacy/position-statements/Physical-Education-is-Essential-for-All-Students.pdf> |
| Australia | Departament of Health of Australia | 2019 | Australian 24-Hour Movement Guidelines for Children (5-12 years) and Young People (13-17 years): An Integration of Physical Activity, Sedentary Behaviour, and Sleep | [https://www1.health.gov.au/internet/main/publishing.nsf/Content/4FA4D308272BD065CA2583D000282813/$File/Australian%2024%20Hour%20Guideline%20Development%20Report%20for%20Children%20and%20Young%20people.pdf](https://www1.health.gov.au/internet/main/publishing.nsf/Content/4FA4D308272BD065CA2583D000282813/$File/Australian 24 Hour Guideline Development Report for Children and Young people.pdf) |
| UK | Association for Physical Education (afPE) | 2019 | The Inspection and Maintenance of Gymnastics, Sports Hall, Fixed Play, Fitness and Sports Equipment | <https://www.afpe.org.uk/physical-education/wp-content/uploads/Inspection-of-Equipment-of-PESSPA-Web.pdf> |
| USA | Society of Health and Physical Educator (SHAPE America) | 2019 | Getting to Know Your Child’s PE Program: A Parent’s Guide | <https://www.shapeamerica.org/uploads/pdfs/2017/downloads/eguides/Parent_Checklist.pdf> |
| International | The common Wealth | 2019 | Measuring the contribution of sport, physical education and physical activity to the Sustainable Development Goals: Toolkit and model indicators v3.1 | <https://thecommonwealth.org/sites/default/files/inline/Sport-SDGs-Indicator-Framework.pdf> |
| International | The common Wealth | 2019 | Model indicators on sport, physical education and physical activity and the Sustainable Development Goals | [https://thecommonwealth.org/sites/default/files/inline/Sport%20and%20SDG%20Indicators%20v3.1.pdf](https://thecommonwealth.org/sites/default/files/inline/Sport and SDG Indicators v3.1.pdf) |
| International | Organisation for Economic Co-operation and Development (OECD) | 2019 | Making Physical Education Dynamic and Inclusive for 2030: International Curriculum Analysis | <https://www.oecd.org/education/2030-project/contact/OECD_FUTURE_OF_EDUCATION_2030_MAKING_PHYSICAL_DYNAMIC_AND_INCLUSIVE_FOR_2030.pdf> |
| USA | Department of Health and Human Services Centers for Disease Control and Prevention (CDC) | 2019 | Parents for Healthy Schools: A Guide for Getting Parents Involved from K–12. | <https://www.cdc.gov/healthyschools/parentsforhealthyschools/pdf/19_306913-A_PHS_guide_new_508_2.pdf> |
| USA | Department of Health and Human Services Centers for Disease Control and Prevention (CDC) | 2019 | Promoting Parent Engagement: Improving Student Health and Academic Achievement | <https://www.cdc.gov/healthyyouth/protective/pdf/parentengagement_administrators.pdf> |
| UK | Association for Physical Education (afPE) | 2020 | Health Position Paper | <https://www.afpe.org.uk/physical-education/wp-content/uploads/Health-Position-Paper-2020-Web.pdf> |
| International | Association for Supervision and Curriculum Development (ASCD) | 2020 | The Learning Compact Renewed Whole Child for The Whole World | <http://files.ascd.org/pdfs/programs/WholeChildNetwork/2020-whole-child-network-learning-compact-renewed.pdf> |
