# Supplementary material for: Mapping recommended strategies to promote active and healthy lifestyles through physical education classes: a scoping review
Source: Int J Behav Nutr Phys Act. 2022 Mar 28;19:36. doi: 10.1186/s12966-022-01278-0 (PMC8962044; doi:10.1186/s12966-022-01278-0)
Supplement: Supplementary file 3 — Additional file 3. [file 12966_2022_1278_MOESM3_ESM.docx]

| Database | Search date | Records identified through database searching |
| --- | --- | --- |
| WEB OF SCIENCE | 06.04.2020 | 381 |
| MEDLINE/PUBMED | 06.04.2020 | 143 |
| LILACS | 06.05.2020 | 231 |
| SCIELO | 06.05.2020 | 160 |
| ERIC Proquest | 06.04.2020 | 376 |
| PsycINFO | 06.04.2020 | 267 |
| Scopus | 06.04.2020 | 311 |
| SPORTDiscus | 06.04.2020 | 443 |
| TOTAL (with duplicates) | | 2.312 |
| TOTAL (excluding duplicates) | | 1638 |
| EXCLUDED | | 674 |

**MEDLINE/PUBMED**

|  | **Descriptors** | **Number of studies reached** |
| --- | --- | --- |
| **#1 – Active lifestyle: PA attributes** | **“Physical Fitness”[MeSH Terms] OR “Physical Fitness”[Text Word] OR “Physical Literacy”[Text Word] OR “Motor Skills”[MeSH Terms] OR ((movement[Text Word]) AND (skills[Text Word] OR repertoire[Text Word] OR pattern*[Text Word] OR vocabulary[Text Word] OR competence*[Text Word] OR capacity*[Text Word] OR capabilit*[Text Word] OR performance[Text Word])) OR ((Motor[Text Word]) AND (abilit*[Text Word] OR development[Text Word] OR performance[Text Word] OR coordination[Text Word] OR skills[Text Word] OR Proficiency[Text Word] OR competence*[Text Word] OR acquisition[Text Word]))** | **245,570** |
| **#2 - Active lifestyle: PA correlates** | **(((Motivational[Text Word] OR Affective[Text Word] OR emotional[Text Word] OR Psychologic*[Text Word] OR Psychosocial[Text Word] OR Cognitive[Text Word] OR physical[Text Word] OR social[Text Word]) AND (capacity[Text Word] OR capacities[Text Word] OR capabilit*[Text Word] OR determinant*[Text Word] OR correlate*[Text Word] OR mediator*[Text Word] OR factor*[Text Word])) OR ((Cultural[Text Word] OR Social[Text Word] OR Sociocultural[Text Word] OR peer*[Text Word] OR family[Text Word] OR parents[Text Word] OR friend*[Text Word]) AND (Support[Text Word] OR Modelling[Text Word] OR Norm*[Text Word] OR Rule*[Text Word])) OR “Social Support”[MeSH Terms] OR “Social Norms”[MeSH Terms] OR “Community Participation”[MeSH Terms] OR “Social Environment”[MeSH Terms] OR “Outdoor Environment”[Text Word] OR “Environmental Health”[MeSH Terms] OR “School Environment”[Text Word] OR “Recreational Environment”[Text Word] OR Safety[MeSH Terms]) AND (“Motor Activity”[MeSH Terms] OR “Physical Activity”[Text Word] OR Physical Activiti*[Text Word] OR Exercise[MeSH Terms] OR "Exercise"[Text Word] OR Exercise*[Text Word] OR Sports[MeSH Terms] OR Sport*[Text Word] OR Movement[Text Word] OR Physical Education[Text Word])** | **185,884** |
| **#3 – Active lifestyle: PA behavior** | **“Motor Activity”[MeSH Terms] OR “Physical Activity”[Text Word] OR Physical Activiti*[Text Word] OR Exercise[MeSH Terms] OR Exercise*[Text Word] OR Sports[MeSH Terms] OR Sport*[Text Word] OR Danc*[Text Word] OR Walking[MeSH Terms] OR “Active Commuting”[Text Word] OR “Active Transport”[Text Word] OR “Leisure Activities”[MeSH Terms] OR Recreation[Text Word] OR “play and playthings"[MeSH Terms] OR "play time"[Text Word] OR "playing" [Text Word]** | **787,358** |
| **#4 – Healthy lifestyle** | “health education”[MeSH Terms] OR “health education”[Text Word] OR “primary prevention”[Text Word] OR “secondary prevention”[Text Word] OR preventive measure*[Text Word] OR preventative measure*[Text Word] OR "Attitude to Health"[MeSH Terms] OR "health status"[MeSH Terms] OR "Health Behavior"[MeSH Terms] OR Health Behavio*[Text Word] OR Life Style[MeSH Terms] OR Lifestyle[Text Word] OR "health"[MeSH Terms] OR **“Body Composition”[MeSH Terms] OR “Body Composition”[Text Word] OR Obesity[Text Word] OR Obesity[Mesh Terms]** | **1,703,422** |
| **#5** | #1 OR #2 OR #3 OR #4 | **2,490,222** |
| **#6 – Context** | **"physical education and training"[MeSH Terms] OR "physical education"[Text Word]** | **15,844** |
| **#7 – Context** | **Schools[Mesh] OR School*[Text Word]** | **352,049** |
| **#8 Type of publication** | **Guidelines[MeSH] OR Guideline[Publication Type] OR Practice Guidelines[MeSH] OR Practice Guideline[Publication Type] OR Consensus Development Conferences[MeSH] OR Consensus Development Conference[Publication Type] OR** statement**[Title] OR recommendation*[Title] OR consensus[Title] OR standard*[Title] OR "position paper"[Title] OR Policy[Title] OR “**position stand”**[Title] OR Guideline*[Title] OR Plan[Title]** | **419,278** |
| **#9** | **#5 AND #6 AND #7 AND #8** | **143** |

**WEB OF SCIENCE**

|  | **Descriptors** | **Number of studies reached** |
| --- | --- | --- |
| **#1 – Active lifestyle: PA attributes** | **TS=(“Physical Fitness” OR “Physical Literacy” OR “Motor Skills”) OR TS=((movement) AND (skills OR repertoire OR pattern* OR vocabulary OR competence* OR capacity* OR capabilit* OR performance)) OR TS= ((Motor) AND (abilit* OR development OR performance OR coordination OR skills OR Proficiency OR competence* OR acquisition))** | **344.077** |
| **#2 - Active lifestyle: PA correlates** | **(TS=((Motivational OR Affective OR emotional OR Psychologic* OR Psychosocial OR Cognitive OR physical OR social) AND (capacity OR capacities OR capabilit* OR determinant* OR correlate* OR mediator* OR factor*)) OR TS=((Cultural OR Social OR Sociocultural OR peer* OR family OR parents OR friend*) AND (Support OR Modelling OR Norm* OR Rule*)) OR TS= (“Social Support” OR “Social Norms” OR “Community Participation” OR “Social Environment” OR “Outdoor Environment” OR “Environmental Health” OR “School Environment” OR “Recreational Environment” OR Safety)) AND TS=(“Motor Activity” OR “Physical Activity” OR “Physical Activities” OR Exercise* OR Sport* OR Movement OR Physical Education)** | **212.711** |
| **#3 – Active lifestyle: PA behavior** | **TS=(“Motor Activity” OR “Physical Activity” OR “Physical Activities” OR Exercise* OR Sport* OR Danc* OR Walking OR “Active Commuting” OR “Active Transport” OR “Leisure Activities” OR Recreation OR “play and playthings" OR "play time" OR "leisure time" OR "playing")** | **1.015.028** |
| **#4 – Healthy lifestyle** | TS=(“health education”OR “primary prevention” OR “secondary prevention” OR preventive measure*OR preventative measure* OR "Attitude to Health"OR "health status" OR Health Behavio*OR “Life Style” OR LifestyleOR "health" OR **“Body Composition”OR Obesity)** | **2.653.633** |
| **#5** | #1 OR #2 OR #3 OR #4 | **3.777.876** |
| **#6 – Context** | **TS=("physical education and training") OR TI=("physical education") OR AB=("physical education")** | **15.904** |
| **#7 – Context** | **TS=(School*)** | **606.716** |
| **#8 Type of publication** | **TS=(Guideline* OR “Practice Guidelines” OR “Practice Guideline” OR “Consensus Development Conferences” OR “Consensus Development Conference”) OR TI=(**Statement **OR recommendation* OR consensus OR standard* OR "position paper" OR Policy OR “**position stand” **OR Guideline* OR Plan)** | **1.337.654** |
| **#9** | **#5 AND #6 AND #7 AND #8** | **381** |

**ERIC – PROQUEST**

|  | **Descriptors** | **Number of studies reached** |
| --- | --- | --- |
| **#1 – Active lifestyle: PA attributes** | **(“Physical Fitness” OR “Physical Literacy” OR “Motor Skills”) OR ((movement) AND (skills OR repertoire OR pattern* OR vocabulary OR competence* OR capacity* OR capabilit* OR performance)) OR ((Motor) AND (abilit* OR development OR performance OR coordination OR skills OR Proficiency OR competence* OR acquisition))** | **25.644** |
| **#2 - Active lifestyle: PA correlates** | **(((Motivational OR Affective OR emotional OR Psychologic* OR Psychosocial OR Cognitive OR physical OR social) AND (capacity OR capacities OR capabilit* OR determinant* OR correlate* OR mediator* OR factor*)) OR ((Cultural OR Social OR Sociocultural OR peer* OR family OR parents OR friend*) AND (Support OR Modelling OR Norm* OR Rule*)) OR (“Social Support” OR “Social Norms” OR “Community Participation” OR “Social Environment” OR “Outdoor Environment” OR “Environmental Health” OR “School Environment” OR “Recreational Environment” OR Safety)) AND (“Motor Activity” OR “Physical Activity” OR “Physical Activities” OR Exercise* OR Sport* OR Movement OR Physical Education)** | **14.289** |
| **#3 – Active lifestyle: PA behavior** | **(“Motor Activity” OR “Physical Activity” OR “Physical Activities” OR Exercise* OR Sport* OR Danc* OR Walking OR “Active Commuting” OR “Active Transport” OR “Leisure Activities” OR Recreation OR “play and playthings" OR "play time" OR "leisure time" OR "playing")** | **79.036** |
| **#4 – Healthy lifestyle** | (“health education” OR “primary prevention” OR “secondary prevention” OR preventive measure* OR preventative measure* OR "Attitude to Health" OR "health status" OR Health Behavio* OR “Life Style” OR Lifestyle OR "health" OR **“Body Composition” OR Obesity)** | **126.978** |
| **#5** | #1 OR #2 OR #3 OR #4 | **212.060** |
| **#6 – Context** | **("physical education and training"OR "physical education")** | **17.084** |
| **#7 – Context** | **(School*)** | **695.146** |
| **#8 Type of publication** | **ti(Guideline* OR “Practice Guidelines” OR “Practice Guideline” OR “Consensus Development Conferences” OR “Consensus Development Conference” OR** Statement **OR recommendation* OR consensus OR standard* OR "position paper" OR Policy OR “**position stand” **OR Guideline* OR Plan)** | **62.047** |
| **#9** | **#5 AND #6 AND #7 AND #8** | **376** |

**SCOPUS**

|  | **Descriptors**  TITLE-ABS-KEY | **Number of studies reached** |
| --- | --- | --- |
| **#1 – Active lifestyle: PA attributes** | TITLE-ABS-KEY **("Physical Fitness" OR "Physical Literacy" OR "Motor Skills") OR** TITLE-ABS-KEY**((movement) AND (skills OR repertoire OR pattern* OR vocabulary OR competence* OR capacity* OR capabilit* OR performance)) OR** TITLE-ABS-KEY**((Motor) AND (abilit* OR development OR performance OR coordination OR skills OR Proficiency OR competence* OR acquisition))** | **613,136** |
| **#2 - Active lifestyle: PA correlates** | TITLE-ABS-KEY**(((Motivational OR Affective OR emotional OR Psychologic* OR Psychosocial OR Cognitive OR physical OR social) AND (capacity OR capacities OR capabilit* OR determinant* OR correlate* OR mediator* OR factor*)) OR ((Cultural OR Social OR Sociocultural OR peer* OR family OR parents OR friend*) AND (Support OR Modelling OR Norm* OR Rule*)) OR ("Social Support" OR "Social Norms" OR " Community Participation" OR "Social Environment" OR "Outdoor Environment" OR "Environmental Health" OR "School Environment" OR "Recreational Environment" OR Safety)) AND** TITLE-ABS-KEY**("Motor Activity" OR "Physical Activity" OR "Physical Activities" OR Exercise* OR Sport* OR Movement OR "Physical Education")** | **324,121** |
| **#3 – Active lifestyle: PA behavior** | TITLE-ABS-KEY**("Motor Activity" OR "Physical Activity" OR "Physical Activities" OR Exercise* OR Sport* OR Danc* OR Walking OR "Active Commuting" OR "Active Transport" OR "Leisure Activities" OR Recreation OR "play and playthings" OR "play time" OR "leisure time" OR "playing")** | **1,423,719** |
| **#4 – Healthy lifestyle** | TITLE-ABS-KEY(**"**health education**"** OR **"**primary prevention**"** OR **"**secondary prevention**"** OR (preventive measure*) OR (preventative measure*) OR **"**Attitude to Health**"** OR **"**health status" OR (Health Behavio*) OR **"**Life Style**"** OR Lifestyle OR health OR**"Body Composition" OR Obesity)** | **5,220,554** |
| **#5** | #1 OR #2 OR #3 OR #4 | **6,820,974** |
| **#6 – Context** | TITLE-ABS-KEY **("physical education and training" OR "physical education")** | **33,791** |
| **#7 – Context** | TITLE-ABS-KEY **(School*)** | **1,191,646** |
| **#8 Type of publication** | TITLE**(Guideline* OR "Practice Guidelines" OR "Practice Guideline" OR "Consensus Development Conferences" OR "Consensus Development Conference" OR Statement OR recommendation* OR consensus OR standard* OR "position paper" OR Policy OR "position stand" OR Plan)** | **862,673** |
| **#9** | **#5 AND #6 AND #7 AND #8** | **311** |

**SPORTDISCUS**

|  | **Descriptors** | **Number of studies reached** |
| --- | --- | --- |
| **#1 – Active lifestyle: PA attributes** | **("Physical Fitness" OR "Physical Literacy" OR "Motor Skills") OR ((movement) AND (skills OR repertoire OR pattern* OR vocabulary OR competence* OR capacity* OR capabilit* OR performance)) OR ((Motor) AND (abilit* OR development OR performance OR coordination OR skills OR Proficiency OR competence* OR acquisition))** | **165,723** |
| **#2 - Active lifestyle: PA correlates** | **(((Motivational OR Affective OR emotional OR Psychologic* OR Psychosocial OR Cognitive OR physical OR social) AND (capacity OR capacities OR capabilit* OR determinant* OR correlate* OR mediator* OR factor*)) OR ((Cultural OR Social OR Sociocultural OR peer* OR family OR parents OR friend*) AND (Support OR Modelling OR Norm* OR Rule*)) OR ("Social Support" OR "Social Norms" OR " Community Participation" OR "Social Environment" OR "Outdoor Environment" OR "Environmental Health" OR "School Environment" OR "Recreational Environment" OR Safety)) AND ("Motor Activity" OR "Physical Activity" OR "Physical Activities" OR Exercise* OR Sport* OR Movement OR "Physical Education")** | **79,948** |
| **#3 – Active lifestyle: PA behavior** | **("Motor Activity" OR "Physical Activity" OR "Physical Activities" OR Exercise* OR Sport* OR Danc* OR Walking OR "Active Commuting" OR "Active Transport" OR "Leisure Activities" OR Recreation OR "play and playthings" OR "play time" OR "leisure time" OR "playing")** | **1,213,892** |
| **#4 – Healthy lifestyle** | (**"**health education**"** OR **"**primary prevention**"** OR **"**secondary prevention**"** OR preventive measure* OR preventative measure* OR **"**Attitude to Health**"** OR **"**health status" OR Health Behavio* OR **"**Life Style**"** OR Lifestyle OR health OR**"Body Composition" OR Obesity)** | **350,384** |
| **#5** | #1 OR #2 OR #3 OR #4 | **1,445,119** |
| **#6 – Context** | **TI ( ("physical education and training" OR "physical education") ) OR AB ( ("physical education and training" OR "physical education") ) OR KW ( ("physical education and training" OR "physical education") )** | **40,510** |
| **#7 – Context** | **School*** | **227,149** |
| **#8 Type of publication** | **TI (Guideline* OR "Practice Guidelines" OR "Practice Guideline" OR "Consensus Development Conferences" OR "Consensus Development Conference" OR Statement OR recommendation* OR consensus OR standard* OR "position paper" OR Policy OR "position stand" OR Plan)** | **28,667** |
| **#9** | **#5 AND #6 AND #7 AND #8** | **443** |

**PSYCHINFO**

|  | **Descriptors** | **Number of studies reached** |
| --- | --- | --- |
| **#1 – Active lifestyle: PA attributes** | **("Physical Fitness" OR "Physical Literacy" OR "Motor Skills") OR ((movement) AND (skills OR repertoire OR pattern* OR vocabulary OR competence* OR capacity* OR capabilit* OR performance)) OR ((Motor) AND (abilit* OR development OR performance OR coordination OR skills OR Proficiency OR competence* OR acquisition))** | **157,023** |
| **#2 - Active lifestyle: PA correlates** | **(((Motivational OR Affective OR emotional OR Psychologic* OR Psychosocial OR Cognitive OR physical OR social) AND (capacity OR capacities OR capabilit* OR determinant* OR correlate* OR mediator* OR factor*)) OR ((Cultural OR Social OR Sociocultural OR peer* OR family OR parents OR friend*) AND (Support OR Modelling OR Norm* OR Rule*)) OR ("Social Support" OR "Social Norms" OR " Community Participation" OR "Social Environment" OR "Outdoor Environment" OR "Environmental Health" OR "School Environment" OR "Recreational Environment" OR Safety)) AND ("Motor Activity" OR "Physical Activity" OR "Physical Activities" OR Exercise* OR Sport* OR Movement OR "Physical Education")** | **117,919** |
| **#3 – Active lifestyle: PA behavior** | **("Motor Activity" OR "Physical Activity" OR "Physical Activities" OR Exercise* OR Sport* OR Danc* OR Walking OR "Active Commuting" OR "Active Transport" OR "Leisure Activities" OR Recreation OR "play and playthings" OR "play time" OR "leisure time" OR "playing")** | **261,729** |
| **#4 – Healthy lifestyle** | (**"**health education**"** OR **"**primary prevention**"** OR **"**secondary prevention**"** OR (preventive AND measure*) OR (preventative AND measure*) OR **"**Attitude to Health**"** OR **"**health status" OR Health Behavio* OR **"**Life Style**"** OR Lifestyle OR health OR**"Body Composition" OR Obesity)** | **1,416,761** |
| **#5** | #1 OR #2 OR #3 OR #4 | **1,663,550** |
| **#6 – Context** | **("physical education and training" OR "physical education")** | **16,329** |
| **#7 – Context** | **School*** | **1,339,734** |
| **#8 Type of publication** | **(Title: Guideline* OR Title: "Practice Guidelines" OR Title: "Practice Guideline" OR Title: "Consensus Development Conferences" OR Title: "Consensus Development Conference" OR Title: Statement OR Title: recommendation* OR Title: consensus OR Title: standard* OR Title: "position paper" OR Title: Policy OR Title: "position stand" OR Title: Plan) OR (Keywords: Guideline* OR Keywords: "Practice Guidelines" OR Keywords: "Practice Guideline" OR Keywords: "Consensus Development Conferences" OR Keywords: "Consensus Development Conference" OR Keywords: Statement OR Keywords: recommendation* OR Keywords: consensus OR Keywords: standard* OR Keywords: "position paper" OR Keywords: Policy OR Keywords: "position stand" OR Keywords: Plan) AND (Abstract: Guideline* OR Abstract: "Practice Guidelines" OR Abstract: "Practice Guideline" OR Abstract: "Consensus Development Conferences" OR Abstract: "Consensus Development Conference" OR Abstract: Statement OR Abstract: recommendation* OR Abstract: consensus OR Abstract: standard* OR Abstract: "position paper" OR Abstract: Policy OR Abstract: "position stand" OR Abstract: Plan)** | **96,765** |
| **#9** | **#5 AND #6 AND #7 AND #8** | **267** |

**LILACS**

|  | **Descriptors** | **Number of studies reached** |
| --- | --- | --- |
| **#1 – Active lifestyle: PA attributes** | **((tw:(skills OR repertoire OR pattern* OR vocabulary OR competence* OR capacity* OR capabilit* OR performance)) AND (tw:(movement))) OR ((tw:(Motor)) AND (tw:(abilit* OR development OR performance OR coordination OR skills OR Proficiency OR competence* OR acquisition))) OR (tw:(("Physical Fitness" OR "Physical Literacy" OR "Motor Skills" OR "Aptidão Física" OR "Letramento Corporal" OR "Letramento Físico" OR "Capacidade Motora" OR "Habilidade Motora" OR "Coordenação Motora" OR "Repertório Motor" OR "Vocabulário Motor" OR "Controle Motor" OR "Padrão de Movimento" OR "competência motora" OR "desempenho Motor" OR "aquisição motora")))** | **8,419** |
| **#2 - Active lifestyle: PA correlates** | **(tw:(((Motivational OR Affective OR emotional OR Psychologic* OR Psychosocial OR Cognitive OR physical OR social) AND (determinant* OR correlate* OR mediator* OR factor*)) OR ((Cultural OR Social OR Sociocultural OR peer* OR family OR parents OR friend*) AND (Support OR Modelling OR Norm* OR Rule*)) OR ("Social Norms" OR "Community Participation" OR "Social Environment" OR "Environmental Health" OR "School Environment" OR "Recreational Environment" OR Safety OR "Suporte Social" OR "Apoio Social" OR "Normas Sociais" OR "Regras Sociais" OR "Participação Comunitária" OR "Ambiente Social" OR "Saúde Ambiental" OR "Ambiente Escolar" OR "Ambiente Recreacional" OR segurança) OR ((motivacional OR afetivo OR psicológico OR psicossocial OR cognitivo OR físico OR social) AND (determinante OR correlato* OR mediador* OR fator*)) OR ((cultural OR social OR sociocultural OR pares OR colega OR família OR pais OR amigo) AND (apoio OR suporte OR support OR modelo OR norma OR regra)))) AND (tw:(("Atividade Motora" OR "Atividade Física" OR exercício OR esporte OR esportes OR movimento OR "Educação Física" OR "Motor Activity" OR "Physical Activity" OR "Physical Activities" OR Exercise* OR Sport* OR Movement OR "Physical Education")))** | **11,007** |
| **#3 – Active lifestyle: PA behavior** | **(tw:("Motor Activity" OR "Physical Activity" OR "Physical Activities" OR Exercise* OR Sport* OR Danc* OR Walking OR "Active Commuting" OR "Active Transport" OR "Leisure Activities" OR Recreation OR "play and playthings" OR "play time" OR "leisure time" OR "playing" OR "Atividade Motora" OR "Atividade Física" OR exercício OR esporte OR esportes OR dança OR caminhada OR ciclismo OR "Transporte Ativo" OR "Deslocamento Ativo" OR "Educação Física" OR "Atividades de Lazer" OR recreação OR "Jogos" OR "Brinquedos" OR brincadeira OR brincar OR "práticas corporais"))** | **39,226** |
| **#4 – Healthy lifestyle** | "health education" OR "primary prevention" OR "secondary prevention" OR (preventive measure*) OR (preventative measure*) OR "Attitude to Health" OR "health status" OR (Health Behavio*) OR "Life Style" OR Lifestyle OR health OR "Body Composition" OR Obesity OR "Educação em Saúde" OR "prevenção" OR "Condição de Saúde" OR "Estilo de vida" OR "Comportamento de saúde" OR "Composição Corporal" OR "Obesidade" | **261,859** |
| **#5** | #1 OR #2 OR #3 OR #4  (tw:("health education" OR "primary prevention" OR "secondary prevention" OR (preventive measure*) OR (preventative measure*) OR "Attitude to Health" OR "health status" OR (Health Behavio*) OR "Life Style" OR Lifestyle OR health OR "Body Composition" OR Obesity OR "Educação em Saúde" OR "prevenção" OR "Condição de Saúde" OR "Estilo de vida" OR "Comportamento de saúde" OR "Composição Corporal" OR "Obesidade")) OR (tw:((tw:("Motor Activity" OR "Physical Activity" OR "Physical Activities" OR Exercise* OR Sport* OR Danc* OR Walking OR "Active Commuting" OR "Active Transport" OR "Leisure Activities" OR Recreation OR "play and playthings" OR "play time" OR "leisure time" OR "playing" OR "Atividade Motora" OR "Atividade Física" OR exercício OR esporte OR esportes OR dança OR caminhada OR ciclismo OR "Transporte Ativo" OR "Deslocamento Ativo" OR "Educação Física" OR "Atividades de Lazer" OR recreação OR "Jogos" OR "Brinquedos" OR brincadeira OR brincar OR "práticas corporais")))) OR (tw:((tw:(((Motivational OR Affective OR emotional OR Psychologic* OR Psychosocial OR Cognitive OR physical OR social) AND (determinant* OR correlate* OR mediator* OR factor*)) OR ((Cultural OR Social OR Sociocultural OR peer* OR family OR parents OR friend*) AND (Support OR Modelling OR Norm* OR Rule*)) OR ("Social Norms" OR "Community Participation" OR "Social Environment" OR "Environmental Health" OR "School Environment" OR "Recreational Environment" OR Safety OR "Suporte Social" OR "Apoio Social" OR "Normas Sociais" OR "Regras Sociais" OR "Participação Comunitária" OR "Ambiente Social" OR "Saúde Ambiental" OR "Ambiente Escolar" OR "Ambiente Recreacional" OR segurança) OR ((motivacional OR afetivo OR psicológico OR psicossocial OR cognitivo OR físico OR social) AND (determinante OR correlato* OR mediador* OR fator*)) OR ((cultural OR social OR sociocultural OR pares OR colega OR família OR pais OR amigo) AND (apoio OR suporte OR support OR modelo OR norma OR regra)))) AND (tw:(("Atividade Motora" OR "Atividade Física" OR exercício OR esporte OR esportes OR movimento OR "Educação Física" OR "Motor Activity" OR "Physical Activity" OR "Physical Activities" OR Exercise* OR Sport* OR Movement OR "Physical Education"))))) OR (tw:(((tw:(skills OR repertoire OR pattern* OR vocabulary OR competence* OR capacity* OR capabilit* OR performance)) AND (tw:(movement))) OR ((tw:(Motor)) AND (tw:(abilit* OR development OR performance OR coordination OR skills OR Proficiency OR competence* OR acquisition))) OR (tw:(("Physical Fitness" OR "Physical Literacy" OR "Motor Skills" OR "Aptidão Física" OR "Letramento Corporal" OR "Letramento Físico" OR "Capacidade Motora" OR "Habilidade Motora" OR "Coordenação Motora" OR "Repertório Motor" OR "Vocabulário Motor" OR "Controle Motor" OR "Padrão de Movimento" OR "competência motora" OR "desempenho Motor" OR "aquisição motora"))))) | **288,144** |
| **#6 – Context** | **("physical education and training" OR "physical education" OR "Educação Física" OR "Educação Física Escolar")** | **3,273** |
| **#7 – Context** | **School* OR Escola*** | **86,250** |
| **#8 Type of publication** | **(Guideline* OR Statement OR recommendation* OR consensus OR standard* OR "position paper" OR Policy OR "position stand" OR Plan OR Guia OR "Guia de Prática" OR "Manual de Prática"OR "Diretrizes de Prática" OR Consens* OR Posicionamento*OR Recomenda* OR Padroniza* OR Política* OR Plano)** | **101,347** |
| **#9** | **#5 AND #6 AND #7 AND #8**  **(tw:((Guideline* OR Statement OR recommendation* OR consensus OR standard* OR "position paper" OR Policy OR "position stand" OR Plan OR Guia OR "Guia de Prática" OR "Manual de Prática" OR "Diretrizes de Prática" OR Consens* OR Posicionamento* OR Recomenda* OR Padroniza* OR Política* OR Plano))) AND (ti:(("physical education and training" OR "physical education" OR "Educação Física" OR "Educação Física Escolar"))) AND (tw:((tw:("health education" OR "primary prevention" OR "secondary prevention" OR (preventive measure*) OR (preventative measure*) OR "Attitude to Health" OR "health status" OR (Health Behavio*) OR "Life Style" OR Lifestyle OR health OR "Body Composition" OR Obesity OR "Educação em Saúde" OR "prevenção" OR "Condição de Saúde" OR "Estilo de vida" OR "Comportamento de saúde" OR "Composição Corporal" OR "Obesidade")) OR (tw:((tw:("Motor Activity" OR "Physical Activity" OR "Physical Activities" OR Exercise* OR Sport* OR Danc* OR Walking OR "Active Commuting" OR "Active Transport" OR "Leisure Activities" OR Recreation OR "play and playthings" OR "play time" OR "leisure time" OR "playing" OR "Atividade Motora" OR "Atividade Física" OR exercício OR esporte OR esportes OR dança OR caminhada OR ciclismo OR "Transporte Ativo" OR "Deslocamento Ativo" OR "Educação Física" OR "Atividades de Lazer" OR recreação OR "Jogos" OR "Brinquedos" OR brincadeira OR brincar OR "práticas corporais")))) OR (tw:((tw:(((Motivational OR Affective OR emotional OR Psychologic* OR Psychosocial OR Cognitive OR physical OR social) AND (determinant* OR correlate* OR mediator* OR factor*)) OR ((Cultural OR Social OR Sociocultural OR peer* OR family OR parents OR friend*) AND (Support OR Modelling OR Norm* OR Rule*)) OR ("Social Norms" OR "Community Participation" OR "Social Environment" OR "Environmental Health" OR "School Environment" OR "Recreational Environment" OR Safety OR "Suporte Social" OR "Apoio Social" OR "Normas Sociais" OR "Regras Sociais" OR "Participação Comunitária" OR "Ambiente Social" OR "Saúde Ambiental" OR "Ambiente Escolar" OR "Ambiente Recreacional" OR segurança) OR ((motivacional OR afetivo OR psicológico OR psicossocial OR cognitivo OR físico OR social) AND (determinante OR correlato* OR mediador* OR fator*)) OR ((cultural OR social OR sociocultural OR pares OR colega OR família OR pais OR amigo) AND (apoio OR suporte OR support OR modelo OR norma OR regra)))) AND (tw:(("Atividade Motora" OR "Atividade Física" OR exercício OR esporte OR esportes OR movimento OR "Educação Física" OR "Motor Activity" OR "Physical Activity" OR "Physical Activities" OR Exercise* OR Sport* OR Movement OR "Physical Education"))))) OR (tw:(((tw:(skills OR repertoire OR pattern* OR vocabulary OR competence* OR capacity* OR capabilit* OR performance)) AND (tw:(movement))) OR ((tw:(Motor)) AND (tw:(abilit* OR development OR performance OR coordination OR skills OR Proficiency OR competence* OR acquisition))) OR (tw:(("Physical Fitness" OR "Physical Literacy" OR "Motor Skills" OR "Aptidão Física" OR "Letramento Corporal" OR "Letramento Físico" OR "Capacidade Motora" OR "Habilidade Motora" OR "Coordenação Motora" OR "Repertório Motor" OR "Vocabulário Motor" OR "Controle Motor" OR "Padrão de Movimento" OR "competência motora" OR "desempenho Motor" OR "aquisição motora"))))))) AND (tw:(School* OR Escola*))** | **231** |

**SCIELO**

|  | **Descriptors** | **Number of studies reached** |
| --- | --- | --- |
| **#1 – Active lifestyle: PA attributes** | **((skills OR repertoire OR pattern* OR vocabulary OR competence* OR capacity* OR capabilit* OR performance) AND (movement)) OR ((Motor) AND (abilit* OR development OR performance OR coordination OR skills OR Proficiency OR competence* OR acquisition)) OR ("Physical Fitness" OR "Physical Literacy" OR "Motor Skills" OR "Aptidão Física" OR "Letramento Corporal" OR "Letramento Físico" OR "Capacidade Motora" OR "Habilidade Motora" OR "Coordenação Motora" OR "Repertório Motor" OR "Vocabulário Motor" OR "Controle Motor" OR "Padrão de Movimento" OR "competência motora" OR "desempenho Motor" OR "aquisição motora")** | **4,712** |
| **#2 - Active lifestyle: PA correlates** | **(((Motivational OR Affective OR emotional OR Psychologic* OR Psychosocial OR Cognitive OR physical OR social) AND (determinant* OR correlate* OR mediator* OR factor*)) OR ((Cultural OR Social OR Sociocultural OR peer* OR family OR parents OR friend*) AND (Support OR Modelling OR Norm* OR Rule*)) OR ("Social Norms" OR "Community Participation" OR "Social Environment" OR "Environmental Health" OR "School Environment" OR "Recreational Environment" OR Safety OR "Suporte Social" OR "Apoio Social" OR "Normas Sociais" OR "Regras Sociais" OR "Participação Comunitária" OR "Ambiente Social" OR "Saúde Ambiental" OR "Ambiente Escolar" OR "Ambiente Recreacional" OR segurança) OR ((motivacional OR afetivo OR psicológico OR psicossocial OR cognitivo OR físico OR social) AND (determinante* OR correlato* OR mediador* OR fator*)) OR ((cultural OR social OR sociocultural OR pares OR colega OR família OR pais OR amigo) AND (apoio OR suporte OR support OR modelo OR norma OR regra)) OR (Cultural OR Social OR Sociocultural OR peer* OR family OR parents OR friend*) AND (Support OR Modelling OR Norm* OR Rule*)) AND (("Atividade Motora" OR "Atividade Física" OR exercício OR esporte OR esportes OR movimento OR "Educação Física" OR "Motor Activity" OR "Physical Activity" OR "Physical Activities" OR Exercise* OR Sport* OR Movement OR "Physical Education"))** | **1,726** |
| **#3 – Active lifestyle: PA behavior** | **("Motor Activity" OR "Physical Activity" OR "Physical Activities" OR Exercise* OR Sport* OR Danc* OR Walking OR "Active Commuting" OR "Active Transport" OR "Leisure Activities" OR Recreation OR "play and playthings" OR "play time" OR "leisure time" OR "playing" OR "Atividade Motora" OR "Atividade Física" OR exercício OR esporte OR esportes OR dança OR caminhada OR ciclismo OR "Transporte Ativo" OR "Deslocamento Ativo" OR "Educação Física" OR "Atividades de Lazer" OR recreação OR "Jogos" OR "Brinquedos" OR brincadeira OR brincar OR "práticas corporais")** | **29,618** |
| **#4 – Healthy lifestyle** | "health education" OR "primary prevention" OR "secondary prevention" OR (preventive measure*) OR (preventative measure*) OR "Attitude to Health" OR "health status" OR (Health Behavio*) OR "Life Style" OR Lifestyle OR health OR "Body Composition" OR Obesity OR "Educação em Saúde" OR "prevenção" OR "Condição de Saúde" OR "Estilo de vida" OR "Comportamento de saúde" OR "Composição Corporal" OR "Obesidade" | **131 084** |
| **#5** | #1 OR #2 OR #3 OR #4  ("health education" OR "primary prevention" OR "secondary prevention" OR (preventive measure*) OR (preventative measure*) OR "Attitude to Health" OR "health status" OR (Health Behavio*) OR "Life Style" OR Lifestyle OR health OR "Body Composition" OR Obesity OR "Educação em Saúde" OR "prevenção" OR "Condição de Saúde" OR "Estilo de vida" OR "Comportamento de saúde" OR "Composição Corporal" OR "Obesidade") OR (("Motor Activity" OR "Physical Activity" OR "Physical Activities" OR Exercise* OR Sport* OR Danc* OR Walking OR "Active Commuting" OR "Active Transport" OR "Leisure Activities" OR Recreation OR "play and playthings" OR "play time" OR "leisure time" OR "playing" OR "Atividade Motora" OR "Atividade Física" OR exercício OR esporte OR esportes OR dança OR caminhada OR ciclismo OR "Transporte Ativo" OR "Deslocamento Ativo" OR "Educação Física" OR "Atividades de Lazer" OR recreação OR "Jogos" OR "Brinquedos" OR brincadeira OR brincar OR "práticas corporais")) OR ((((Motivational OR Affective OR emotional OR Psychologic* OR Psychosocial OR Cognitive OR physical OR social) AND (determinant* OR correlate* OR mediator* OR factor*)) OR ((Cultural OR Social OR Sociocultural OR peer* OR family OR parents OR friend*) AND (Support OR Modelling OR Norm* OR Rule*)) OR ("Social Norms" OR "Community Participation" OR "Social Environment" OR "Environmental Health" OR "School Environment" OR "Recreational Environment" OR Safety OR "Suporte Social" OR "Apoio Social" OR "Normas Sociais" OR "Regras Sociais" OR "Participação Comunitária" OR "Ambiente Social" OR "Saúde Ambiental" OR "Ambiente Escolar" OR "Ambiente Recreacional" OR segurança) OR ((motivacional OR afetivo OR psicológico OR psicossocial OR cognitivo OR físico OR social) AND (determinante* OR correlato* OR mediador* OR fator*)) OR ((cultural OR social OR sociocultural OR pares OR colega OR família OR pais OR amigo) AND (apoio OR suporte OR support OR modelo OR norma OR regra)) OR (Cultural OR Social OR Sociocultural OR peer* OR family OR parents OR friend*) AND (Support OR Modelling OR Norm* OR Rule*)) AND (("Atividade Motora" OR "Atividade Física" OR exercício OR esporte OR esportes OR movimento OR "Educação Física" OR "Motor Activity" OR "Physical Activity" OR "Physical Activities" OR Exercise* OR Sport* OR Movement OR "Physical Education"))) OR (((skills OR repertoire OR pattern* OR vocabulary OR competence* OR capacity* OR capabilit* OR performance) AND (movement)) OR ((Motor) AND (abilit* OR development OR performance OR coordination OR skills OR Proficiency OR competence* OR acquisition)) OR ("Physical Fitness" OR "Physical Literacy" OR "Motor Skills" OR "Aptidão Física" OR "Letramento Corporal" OR "Letramento Físico" OR "Capacidade Motora" OR "Habilidade Motora" OR "Coordenação Motora" OR "Repertório Motor" OR "Vocabulário Motor" OR "Controle Motor" OR "Padrão de Movimento" OR "competência motora" OR "desempenho Motor" OR "aquisição motora")) | **153,787** |
| **#6 – Context** | **("physical education and training" OR "physical education" OR "Educação Física" OR "Educação Física Escolar")** | **2 815** |
| **#7 – Context** | **School* OR Escola*** | **55 497** |
| **#8 Type of publication** | **(Guideline* OR Statement OR recommendation* OR consensus OR standard* OR "position paper" OR Policy OR "position stand" OR Plan OR Guia OR "Guia de Prática" OR "Manual de Prática"OR "Diretrizes de Prática" OR Consens* OR Posicionamento*OR Recomenda* OR Padroniza* OR Política* OR Plano)** | **147 672** |
| **#9** | **#5 AND #6 AND #7 AND #8**  **(("health education" OR "primary prevention" OR "secondary prevention" OR (preventive measure*) OR (preventative measure*) OR "Attitude to Health" OR "health status" OR (Health Behavio*) OR "Life Style" OR Lifestyle OR health OR "Body Composition" OR Obesity OR "Educação em Saúde" OR "prevenção" OR "Condição de Saúde" OR "Estilo de vida" OR "Comportamento de saúde" OR "Composição Corporal" OR "Obesidade") OR (("Motor Activity" OR "Physical Activity" OR "Physical Activities" OR Exercise* OR Sport* OR Danc* OR Walking OR "Active Commuting" OR "Active Transport" OR "Leisure Activities" OR Recreation OR "play and playthings" OR "play time" OR "leisure time" OR "playing" OR "Atividade Motora" OR "Atividade Física" OR exercício OR esporte OR esportes OR dança OR caminhada OR ciclismo OR "Transporte Ativo" OR "Deslocamento Ativo" OR "Educação Física" OR "Atividades de Lazer" OR recreação OR "Jogos" OR "Brinquedos" OR brincadeira OR brincar OR "práticas corporais")) OR ((((Motivational OR Affective OR emotional OR Psychologic* OR Psychosocial OR Cognitive OR physical OR social) AND (determinant* OR correlate* OR mediator* OR factor*)) OR ((Cultural OR Social OR Sociocultural OR peer* OR family OR parents OR friend*) AND (Support OR Modelling OR Norm* OR Rule*)) OR ("Social Norms" OR "Community Participation" OR "Social Environment" OR "Environmental Health" OR "School Environment" OR "Recreational Environment" OR Safety OR "Suporte Social" OR "Apoio Social" OR "Normas Sociais" OR "Regras Sociais" OR "Participação Comunitária" OR "Ambiente Social" OR "Saúde Ambiental" OR "Ambiente Escolar" OR "Ambiente Recreacional" OR segurança) OR ((motivacional OR afetivo OR psicológico OR psicossocial OR cognitivo OR físico OR social) AND (determinante* OR correlato* OR mediador* OR fator*)) OR ((cultural OR social OR sociocultural OR pares OR colega OR família OR pais OR amigo) AND (apoio OR suporte OR support OR modelo OR norma OR regra)) OR (Cultural OR Social OR Sociocultural OR peer* OR family OR parents OR friend*) AND (Support OR Modelling OR Norm* OR Rule*)) AND (("Atividade Motora" OR "Atividade Física" OR exercício OR esporte OR esportes OR movimento OR "Educação Física" OR "Motor Activity" OR "Physical Activity" OR "Physical Activities" OR Exercise* OR Sport* OR Movement OR "Physical Education"))) OR (((skills OR repertoire OR pattern* OR vocabulary OR competence* OR capacity* OR capabilit* OR performance) AND (movement)) OR ((Motor) AND (abilit* OR development OR performance OR coordination OR skills OR Proficiency OR competence* OR acquisition)) OR ("Physical Fitness" OR "Physical Literacy" OR "Motor Skills" OR "Aptidão Física" OR "Letramento Corporal" OR "Letramento Físico" OR "Capacidade Motora" OR "Habilidade Motora" OR "Coordenação Motora" OR "Repertório Motor" OR "Vocabulário Motor" OR "Controle Motor" OR "Padrão de Movimento" OR "competência motora" OR "desempenho Motor" OR "aquisição motora"))) AND (("physical education and training" OR "physical education" OR "Educação Física" OR "Educação Física Escolar")) AND (School* OR Escola*) AND ((Guideline* OR Statement OR recommendation* OR consensus OR standard* OR "position paper" OR Policy OR "position stand" OR Plan OR Guia OR "Guia de Prática" OR "Manual de Prática" OR "Diretrizes de Prática" OR Consens* OR Posicionamento* OR Recomenda* OR Padroniza* OR Política* OR Plano))** | **160** |
