# Supplementary material for: Mapping recommended strategies to promote active and healthy lifestyles through physical education classes: a scoping review
Source: Int J Behav Nutr Phys Act. 2022 Mar 28;19:36. doi: 10.1186/s12966-022-01278-0 (PMC8962044; doi:10.1186/s12966-022-01278-0)
Supplement: Supplementary file 4 — Additional file 4. [file 12966_2022_1278_MOESM4_ESM.doc]

SUPPLEMENTARY MATERIAL 02

Synthesis of the variables extracted from the recommendation documents on PE strategies for the promotion of an active and healthy lifestyle.

| Groups of information | Extracted variables |
| --- | --- |
| Group 1: Identification and description of documents and population. | - - - Reference;     - Is PE primary in the document? (yes/not);     - Institution/organization     - Geographic scope of the institution/organization;     - Year;     - Type of document;     - Main goal;     - Description of the methodological process for constructing the document;     - Target population (students, teachers, schools managers, politicians, and parents/relatives). |
| Group 2 – Recommended strategies according dimensions | - - - Policy and environment;     - Curriculum;     - Appropriate instruction;     - Student assessment;     - Other practices related to PE. |
