# Supplementary material for: Mapping recommended strategies to promote active and healthy lifestyles through physical education classes: a scoping review
Source: Int J Behav Nutr Phys Act. 2022 Mar 28;19:36. doi: 10.1186/s12966-022-01278-0 (PMC8962044; doi:10.1186/s12966-022-01278-0)
Supplement: Supplementary file 5 — Additional file 5. [file 12966_2022_1278_MOESM5_ESM.doc]

Articles included in the studies

| **Publication Year** | **Organization** | **Author** | **Title** |
| --- | --- | --- | --- |
| 2000 | National Association of State Boards of Education (NASBE) | Bogden, J. F. | Fit, Healthy, and Ready To Learn: A School Health Policy Guide. Part I: Physical Activity, Health Eating, and Tobacco-Use Prevention |
| 2001 | Centers for Disease Control and Prevention (CDC) | ------ | Increasing physical activity. A report on recommendations of the Task Force on Community Preventive Services |
| 2001 | ------ | Lee Haney, Chair | Physical education statement by the President's Council on Physical Fitness and Sports |
| 2003 | Centers for Disease Control and Prevention (CDC) | Barrios, L. C.; Sleet, D. A.; Mercy, J. A. | CDC School Health Guidelines to Prevent Unintentional Injuries and Violence |
| 2004 | American Heart Association (AHS) | Hayman, L. L.; Williams, C. L.; Daniels, S. R. et al. | Cardiovascular health promotion in the schools: a statement for health and education professionals and child health advocates from the Committee on Atherosclerosis, Hypertension, and Obesity in Youth (AHOY) of the Council on Cardiovascular Disease in the Young, American Heart Association |
| 2006 | American Academy of Pediatrics (AAP) | ------ | Active healthy living: prevention of childhood obesity through increased physical activity |
| 2006 | ------ | Pate, R. R.; Davis, M. G.; Robinson, T. N.et al. | Promoting physical activity in children and youth: A leadership role for schools - A scientific statement from the American Heart Association Council on Nutrition, Physical Activity, and Metabolism (Physical Activity Committee) in collaboration with the Councils on Cardiovascular Disease in the Young and Cardiovascular Nursing |
| 2008 | Centers for Disease Control and Prevention (CDC) | ------ | A CDC Review of School Laws and Policies Concerning Child and Adolescent Health |
| 2008 | National Association for Sport and Physical Education. | ------ | A Position Statement from the National Association for Sport and Physical Education: Comprehensive School Physical Activity Program |
| 2008 | American Heart Association (AHS) | Pate, R. R.; O'Neill, J. R. | Summary of the American Heart Association scientific statement: promoting physical activity in children and youth: a leadership role for schools |
| 2011 | Global Forum for Physical Education Pedagogy 2010 (GoFPEP 2010) | ------ | Health and Physical Education Pedagogy in the 21st Century-A Statement of Consensus |
| 2011 | Centers for Disease Control and Prevention (CDC) | Lee, S. M. | School health guidelines to promote healthy eating and physical activity |
| 2012 | Ministerio de Salud Pública y Bienestar Social | ------ | Directrices de Evaluación de Niñas, Niños y Adolescentes para la Actividad Física Pedagógica Recreativa y Deportiva Escolar en Paraguay |
| 2014 | UK Strength and Conditioning Association on youth resistance training | Lloyd, R. S.; Faigenbaum, A. D.; Stone, M. H. et al. | Position statement on youth resistance training: the 2014 International Consensus |
| 2014 | ------ | Graf, C.; Beneke, R.; Bloch, W. et al. | Recommendations for promoting physical activity for children and adolescents in Germany. A consensus statement |
| 2014 | ------ | Buscemi, J.; Kong, A.; Fitzgibbon, M. L. et al. | Society of Behavioral Medicine position statement: elementary school-based physical activity supports academic achievement |
