# Supplementary material for: Mapping recommended strategies to promote active and healthy lifestyles through physical education classes: a scoping review
Source: Int J Behav Nutr Phys Act. 2022 Mar 28;19:36. doi: 10.1186/s12966-022-01278-0 (PMC8962044; doi:10.1186/s12966-022-01278-0)
Supplement: Supplementary file 6 — Additional file 6. [file 12966_2022_1278_MOESM6_ESM.doc]

**LIST OF EXCLUDED STUDIES AND JUSTIFICATION**

| **Publication Year** | **Author** | **Title** | **reason for the exclusion** |
| --- | --- | --- | --- |
| 1966 | Reichert, J. L. et al. | Suggested School Health Policies. Fourth Edition | Published more than 20 years ago |
| 1970 | ------- | Guidelines for secondary school physical education: a position paper | Published more than 20 years ago |
| 1971 | ------- | Guidelines for Secondary School Physical Education. A Position Paper | Published more than 20 years ago |
| 1974 | ------- | What the Society of State Directors of Health, Physical Education, and Recreation believes about school health education: A statement of basic beliefs | Published more than 20 years ago |
| 1977 | Holbrook, L. | Current Status of Physical Education, Sport and Active Recreation. Documentary Statement | Published more than 20 years ago |
| 1978 | Watkins, W. | Administrative guidelines for community use of physical education and recreation facilities | Published more than 20 years ago |
| 1978 | ------- | Guidelines for Physical Education in the Secondary Schools | Published more than 20 years ago |
| 1979 | ------- | Guidelines for Secondary School Physical Education. A Position Paper. Revised | Published more than 20 years ago |
| 1982 | Abbott, C. | Developing a school physical education curriculum policy | Published more than 20 years ago |
| 1983 | ------- | ACHPER position statement: quality physical education in Australian schools | Published more than 20 years ago |
| 1983 | Turkington, D.; Wall, J.; Alberts, P. et al. | Secondary school physical education: a position paper prepared by the School Physical Activities Programs Committee. / Education physique au niveau secondaire: document de principe prepare par le Comite des programmes d'activite physique scolaire | Published more than 20 years ago |
| 1986 | ------- | Guidelines for Middle School Physical Education. A Position Paper. Revised 1986 | Published more than 20 years ago |
| 1986 | ------- | Guidelines for Secondary School Physical Education. A Position Paper. Revised 1986 | Published more than 20 years ago |
| 1988 | ------- | Health-related fitness testing and monitoring in schools: a position statement on behalf of the Physical Education Association by its Fitness and Health Advisory Committee | Published more than 20 years ago |
| 1991 | Greene, L.; Adeyanju, M. | Exercise and Fitness Guidelines for Elementary and Middle School Children | Published more than 20 years ago |
| 1991 | ------- | Physical Education Model Curriculum Standards. Grades Nine through Twelve | Published more than 20 years ago |
| 1991 | ------- | Physical Education Outcomes: Grades K-5. Report of the Task Force To Develop HPERD Program Standards | Published more than 20 years ago |
| 1992 | ------- | Dietary Guidelines and Your Health: Health Educator's Guide to Nutrition and Fitness | Published more than 20 years ago |
| 1993 | ------- | Physical Education Outcomes: Grades 6-8, Report of the Task Force To Develop HPERD Program Standards | Published more than 20 years ago |
| 1994 | ------- | A statement on health and physical education for Australian schools | Published more than 20 years ago |
| 1995 | ------- | National Health Education Standards: Achieving Health Literacy | Published more than 20 years ago |
| 1995 | Ross, J. | Physical activity guidelines and compliance of New Zealand adolescents and children | Published more than 20 years ago |
| 1995 | Fried, D. H. | Recommendations for the safe and proper conduct of physical education, athletics and recreational programs | Published more than 20 years ago |
| 1995 | Kolbe, Lloyd J.; et al. | The School Health Policies and Programs Study (SHPPS): Context, Methods, General Findings, and Future Efforts | Published more than 20 years ago |
| 1997 | ------- | Guidelines for school and community programs to promote lifelong physical activity among young people. National Center for Chronic Disease Prevention and Health Promotion, Centers for Disease Control and prevention | Published more than 20 years ago |
| 1997 | Fletcher, G. F. | How to implement physical activity in primary and secondary prevention. A statement for healthcare-professionals from the Task Force on Risk-reduction, American Heart Association | Published more than 20 years ago |
| 1997 | Leijten, C. CY -; | AIESEP Singapore 1997 World Conference on Teaching, Coaching and Fitness Needs in Physical Education and the Sport Sciences. Proceedings, School of Physical Education : Nanyang Technological University, p.115-124. | Published more than 20 years ago |
| 1997 | Young, J. C. | National standards for physical education, nutrition, health, and safety | Published more than 20 years ago |
| 1997 | Morgan, I. | Standards and quality in primary and secondary schools good practice in physical education and sport | Published more than 20 years ago |
| 1998 | Sammann, P. | Active youth: ideas for implementing CDC physical activity promotion guidelines | Published more than 20 years ago |
| 1999 | Strand, B.; Roesler, K. | Calorie Education: A New Plan of Study in Physical Education | Published more than 20 years ago |
| 1999 | ------- | Japanese Government Policies in Education, Science, Sports and Culture, 1998. Mental and Physical Health and Sports | Published more than 20 years ago |
| 2001 |  | CDC Releases School Health Policies and Programs Study Findings | Ineligible documents |
| 2002 | ------- | Co-curricular physical activity and sport programs for middle school students: a position statement | No specific recommendations for PE |
| 2002 | Decker, J. I.; Mize, M. | Walking games and activities, Champaign, Ill., Human Kinetics, 2002. | Ineligible documents |
| 2002 | Schiemer, S. | Proposed academic standards for health, safety, physical education: 10.5 concepts, principles and strategies of movement | Local / regional coverage |
| 2005 | Allen, J. B.; Petrie, K. C. | Connecting through Physical Education: A Position Paper Exploring Social Connectedness | Ineligible documents |
| 2005 | Nakai, T.; Metzler, M. W. | Standards and Practice for K-12 Physical Education in Japan: In Both Content and Approach, Japanese Physical Education Exhibits Similarities to, and Differences from, Physical Education in the United States | Ineligible documents |
| 2005 | Wright, S. C.; McNeill, M. C.; Schempp, P. G. | Standards and Practice for K-12 Physical Education in Singapore | Ineligible documents |
| 2005 | Wright, S. C.; McNeill, M. C.; Schempp, P. G. | Standards and Practice for K-12 Physical Education in Singapore: Although Physical Education is Required at All Grade Levels in Singapore, Physical Educators There Still Battle Marginalization of Their Subject | Duplicate content |
| 2005 | Liang, G; Walls, R. T.; Lu, C. | Standards and Practice for Physical Education in China | Ineligible documents |
| 2005 | Yoo, Sang Suk; Kim, Ha Young | Standards and Practice in Korean Physical Education: Six Revisions of the National Physical Education Curriculum have Brought Greater Autonomy to Schools and Teachers, Yet Better Teacher Practices are Still Needed | Ineligible documents |
| 2008 | Scruggs, P. W.; Mungen, J. D.; Oh, Y. | Physical activity guideline compliance in US middle school physical education | Ineligible documents |
| 2008 | ------- | Comprehensive School Physical Activity Programs. Position Statement | Duplicate content |
| 2008 | Trost, S. G.; Loprinzi, P. D. | Exercise-Promoting healthy lifestyles in children and adolescents | Ineligible documents |
| 2008 | Emmel, J. | The Curriculum Future of Health and Physical Education in Australia: A National Statement and National Action Plan | Local / regional coverage |
| 2009 | Faigenbaum, A. D.; Kraemer, W. J. et al. | Youth resistance training: updated position statement paper from the national strength and conditioning association | No specific recommendations for PE |
| 2009 | Shu-yan, Wang; Xiao-ting, Sun | An Analysis of Influencing Factors on Policy Execution in School Sports | Ineligible documents |
| 2009 | ------- | CDC reviews school laws, policies on child/adolescent health | Duplicate content |
| 2009 | Dobay, B.; Diosi, E. | GUIDELINES OF PHYSICAL EDUCATION IN LOWER DEPARTMENT OF ELEMENTARY SCHOOLS | Ineligible documents |
| 2009 | Leatherdale, S. T.; Manske, S.; Wong, S. L. et al. | Integrating Research, Policy, and Practice in School-Based Physical Activity Prevention Programming: The School Health Action, Planning, and Evaluation System (SHAPES) Physical Activity Module | No specific recommendations for PE |
| 2010 | Larson, James S.; Winn, Mylon | Health Policy and Exercise: A Brief BRFSS Study and Recommendations | No specific recommendations for PE |
| 2011 | Edginton, C. R.; Chin, M. K.; Geadelmann, P. L. et al. | Global Forum for Physical Education Pedagogy 2010 (GoFPEP 2010): Health and Physical Education Pedagogy in the 21st Century - A Statement of Consensus | Local / regional coverage |
| 2011 | Eyler, Amy A. | Promoting Physical Activity through Policy | Ineligible documents |
| 2011 | Edginton, C. R.; Ming-kai, C.; Geadelmann, P. L. et al. | Revitalizing Health and Physical Education in the 21st Century: A Global Statement of Consensus | Duplicate content |
| 2011 | ------- | School Health Guidelines to Promote Healthy Eating and Physical Activity: Executive Summary | Duplicate content |
| 2011 | Radojević, J. | STANDARDS IN FUNCTION OF QUALITY PROMOTION IN PHYSICAL EDUCATION | Local / regional coverage |
| 2013 | Wei, Xiao; Ben-lian, W. U. | Compulsory physical education and health curriculum standard implementation strategies | Ineligible documents |
| 2013 | Erwin, P; Fitzhugh, E. C; Frederick, V. et al | Policies to Increase Youth Physical Activity in School and Community Settings | Duplicate content |
| 2013 | Yubin, H. A. N.; Landian, W. E. N. | Post Modern Perspective on Curricular View of Physical Education, Physicion Education and Health Curricular Standard | Ineligible documents |
| 2013 | Ming, H. A. O. | Strategies for promoting the effective implementation of physical education and health curriculum standard | Ineligible documents |
| 2015 | Fobbs, E. | Promoting Student Achievement through Improved Health Policy | Local / regional coverage |
| 2015 | Hills, A. P.; Dengel, D. R.; Lubans, D. R. | Supporting public health priorities: recommendations for physical education and physical activity promotion in schools | Ineligible documents |
| 2016 | Pate, R. R.; Flynn, J. I.; Dowda, M. | Policies for promotion of physical activity and prevention of obesity in adolescence | Local / regional coverage |
| 2016 | Skovgaard, Thomas | Sport in Danish Schools: Physical Activity and Public Policy Formulation in Denmark | Local / regional coverage |
| 2017 | Neumann, L.; Combe, L.; Lambert, P. et al. | Whole School, Whole Community, Whole Child: Implications for 21st Century School Nurses. Position Statement | No specific recommendations for PE |
| 2019 | Barnett, L. M.; Dudley, D. A.; Telford, R. D. et al. | Guidelines for the Selection of Physical Literacy Measures in Physical Education in Australia. | Local / regional coverage |

| **Full-text articles excluded, with reasons** | **Amount** |
| --- | --- |
| • Published more than 20 years ago | 32 |
| • Ineligible documents | 16 |
| • Duplicate content | 6 |
| • Local / regional coverage | 8 |
| • No specific recommendations for PE | 5 |
