# Supplementary material for: Mapping recommended strategies to promote active and healthy lifestyles through physical education classes: a scoping review
Source: Int J Behav Nutr Phys Act. 2022 Mar 28;19:36. doi: 10.1186/s12966-022-01278-0 (PMC8962044; doi:10.1186/s12966-022-01278-0)
Supplement: Supplementary file 7 — Additional file 7. [file 12966_2022_1278_MOESM7_ESM.docx]

**7. Supplement Material 07 – References for each recommendation**

| **POLICY AND ENVIRONMENT** | | | |
| --- | --- | --- | --- |
| **Recommendations** | | | **References** |
| Recognizing the importance of the PE, financing, and providing better working conditions | Recognizing the importance of the PE and providing better working conditions | 1 - Recognition of PE as well as other subjects (n=7) | 1. Bogden JF. Fit, Healthy, and Ready To Learn: A School Health Policy Guide. Part I: Physical Activity, Health Eating, and Tobacco-Use Prevention. 2000 Mar;1–230.  2. National Association for Sport and Physical Education. A Position Statement from the National Association for Sport and Physical Education: Comprehensive School Physical Activity Program. Strategies: A Journal for Physical and Sport Educators. 2008 Jul;21(6):29–33.  3. SHAPE America-Society of Health and Physical Educators. The essential components of physical education. Author Reston, VA; 2015.  4. SHAPE America. Shape of the Nation Status of Physical Education in the USA [Internet]. 2016 [cited 2020 Jul 5]. Available from: https://www.shapeamerica.org/advocacy/son/2016/upload/Shape-of-the-Nation-2016_web.pdf  5. SHAPE America. Opportunity to Learn: Guidelines for Elementary, Middle & High School Physical Education - A Side‐by‐Side Comparison [Internet]. 2010 [cited 2020 Jul 5]. Available from: https://www.shapeamerica.org/standards/guidelines/upload/Opportunity-to-Learn-Grid.pdf  6. European Physical Education Association. European Framework of Quality Physical Education [Internet]. 2018 [cited 2020 Jul 5]. Available from: http://www.eupea.com/wp-content/uploads/2018/02/European-Framework-of-Quality-PE.pdf  7. SHAPE America. Appropriate Instructional Practice Guidelines, K-12: A Side-by-Side Comparison SHAPE America – Society of Health and Physical Educators [Internet]. 2009 [cited 2020 Jul 5]. Available from: https://www.shapeamerica.org/upload/Appropriate-Instructional-Practice-Guidelines-K-12.pdf  8. OLIVEIRA ARC de, SARTORI SK, LAURINDO E. Recomendações para a Educação Física escolar. Sistema CONFEF/CREFs Conselhos Federal e Regionais de Educação Física. 2014;  9. UNESCO. Diretrizes em educação física de qualidade (EFQ) para gestores de políticas [Internet]. 2015 [cited 2020 Jul 5]. Available from: https://unesdoc.unesco.org/ark:/48223/pf0000231963  10. SHAPE America. Getting to Know Your Child’s PE Program: A Parent’s Guide [Internet]. 2019 [cited 2020 Jul 5]. Available from: https://www.shapeamerica.org/uploads/pdfs/2017/downloads/eguides/Parent_Checklist.pdf  11. American Heart Association. Increasing and Improving Physical Education and Physical Activity in Schools: Benefits for Children’s Health and Educational Outcomes [Internet]. 2015 [cited 2020 Jul 5]. Available from: https://www.heart.org/idc/groups/heart-public/@wcm/@adv/documents/downloadable/ucm_473782.pdf  12. SHAPE America. Physical Education is Essential for All Students: No Substitutions, Waivers or Exemptions for Physical Education. 2018;6. |
|  |  | 2 - Managers need to provide adequate working conditions (n=4) | 1. OLIVEIRA ARC de, SARTORI SK, LAURINDO E. Recomendações para a Educação Física escolar. Sistema CONFEF/CREFs Conselhos Federal e Regionais de Educação Física. 2014;  2. International Council of Sport Science and Physical EducationScience Education Policy. International Position Statement on Physical Education. 2010.  3. Kay T, Dudfield O, Commonwealth Secretariat. The commonwealth guide to advancing development through sport. London: Commonwealth Secretariat; 2013.  4. International Council of Sport Science and Physical EducationScience Education Policy. International Benchmarks for Physical Education Systems Developed by ICSSPE’s International Committee of Sport Pedagogy. 2012.  5. Association for Supervision and Curriculum Development (ASCD). ASCD’s Position on the Whole Child [Internet]. 2020 [cited 2020 Jul 5]. Available from: http://files.ascd.org/pdfs/programs/WholeChildNetwork/2020-whole-child-network-learning-compact-renewed.pdf  6. Costa Rica. Ministerio de Salud y Ministerio de Deporte y Recreación. Plan Nacional de Actividad Física y Salud 2011-2021. 2011.  7. The common Wealth. Measuring the contribution of sport, physical education and physical activity to the Sustainable Development Goals Toolkit and model indicators [Internet]. 2019 [cited 2020 Jul 5]. Available from: https://thecommonwealth.org/sites/default/files/inline/Sport-SDGs-Indicator-Framework.pdf |
|  | 2 - Financing for the purchase and maintenance of spaces, equipment, and materials | 3 - Funding for PE programs, including adequate spaces and materials (n=9) | 1. ParticipACTION Advisory Groups. Active Canada 20/20: A physical activity strategy and change agenda for Canada. 2012.  2. International Council of Sport Science and Physical EducationScience Education Policy. International Position Statement on Physical Education. 2010.  3. UNESCO. Declaration of Berlin: International Conference of Ministers and Senior Officials Responsible for Physical Education and Sport [Internet]. 2013 [cited 2020 Jul 5]. Available from: https://unesdoc.unesco.org/ark:/48223/pf0000221114  4. UNESCO. Diretrizes em educação física de qualidade (EFQ) para gestores de políticas [Internet]. 2015 [cited 2020 Jul 5]. Available from: https://unesdoc.unesco.org/ark:/48223/pf0000231963  5. Council on Sports Medicine and Fitness and Council on School Health. Active healthy living: prevention of childhood obesity through increased physical activity. Pediatrics. 2006/05/03 ed. 2006 May;117(5):1834–42.  6. SHAPE America. Opportunity to Learn: Guidelines for Elementary, Middle & High School Physical Education - A Side‐by‐Side Comparison [Internet]. 2010 [cited 2020 Jul 5]. Available from: https://www.shapeamerica.org/standards/guidelines/upload/Opportunity-to-Learn-Grid.pdf  7. SHAPE America. Guide for the Physical Education Policy [Internet]. 2016 [cited 2020 Jul 5]. Available from: https://www.shapeamerica.org/advocacy/upload/Guide-for-Physical-Education-Policy-9-23-14.pdf  8. Department of Health (Ireland). Get Ireland Active! The National Physical Activity Plan for Ireland [Internet]. 2016 [cited 2020 Jul 5]. Available from: https://assets.gov.ie/12198/5f3dbab207f2464bba3b9b3f6d02bff6.pdf  9. Kay T, Dudfield O, Commonwealth Secretariat. The commonwealth guide to advancing development through sport. London: Commonwealth Secretariat; 2013.  10. The common Wealth. Measuring the contribution of sport, physical education and physical activity to the Sustainable Development Goals Toolkit and model indicators [Internet]. 2019 [cited 2020 Jul 5]. Available from: https://thecommonwealth.org/sites/default/files/inline/Sport-SDGs-Indicator-Framework.pdf  11. Bogden JF. Fit, Healthy, and Ready To Learn: A School Health Policy Guide. Part I: Physical Activity, Health Eating, and Tobacco-Use Prevention. 2000 Mar;1–230.  12. Lee SM. School health guidelines to promote healthy eating and physical activity. Morbidity and Mortality Weekly Report. 2011;60(RR-5):1–78.  13. Federation Internationale d´Éducation Physique. The World Manifest of Physical Education FIEP 2000 [Internet]. 2000 [cited 2020 Jul 5]. Available from: http://fiepeurope.eu/manifest.php  14. American Heart Association. Increasing and Improving Physical Education and Physical Activity in Schools: Benefits for Children’s Health and Educational Outcomes [Internet]. 2015 [cited 2020 Jul 5]. Available from: https://www.heart.org/idc/groups/heart-public/@wcm/@adv/documents/downloadable/ucm_473782.pdf  15. UNESCO. International Charter of Physical Education, Physical Activity and Sport [Internet]. 2015 [cited 2020 Jul 5]. Available from: https://unesdoc.unesco.org/ark:/48223/pf0000235409  16. The common Wealth. Sport for Development and Peace and the 2030 Agenda for Sustainable Development [Internet]. 2015 [cited 2020 Jul 5]. Available from: https://thecommonwealth.org/sites/default/files/inline/CW_SDP_2030%2BAgenda.pdf |
|  |  | 4 - Use of appropriate technologies in PE classes (n=1) | 1. European Physical Education Association. European Framework of Quality Physical Education [Internet]. 2018 [cited 2020 Jul 5]. Available from: http://www.eupea.com/wp-content/uploads/2018/02/European-Framework-of-Quality-PE.pdf |
|  |  | 5 - Regular maintenance of materials and equipment for PE classes (n=4) | 1. SHAPE America. Opportunity to Learn: Guidelines for Elementary, Middle & High School Physical Education - A Side‐by‐Side Comparison [Internet]. 2010 [cited 2020 Jul 5]. Available from: https://www.shapeamerica.org/standards/guidelines/upload/Opportunity-to-Learn-Grid.pdf  2. SHAPE America. Appropriate Instructional Practice Guidelines, K-12: A Side-by-Side Comparison SHAPE America – Society of Health and Physical Educators [Internet]. 2009 [cited 2020 Jul 5]. Available from: https://www.shapeamerica.org/upload/Appropriate-Instructional-Practice-Guidelines-K-12.pdf  3. Association for Physical Education (afPE). The Inspection and Maintenance of Gymnastics, Sports Hall, Fixed Play, Fitness and Sports Equipment [Internet]. 2019 [cited 2020 Jul 5]. Available from: https://www.afpe.org.uk/physical-education/wp-content/uploads/Inspection-of-Equipment-of-PESSPA-Web.pdf |
|  | 3 - Adequate working conditions and equipment | 6 - Proper use of facilities and equipment, including the natural environment (n=4) | 1. Bogden JF. Fit, Healthy, and Ready To Learn: A School Health Policy Guide. Part I: Physical Activity, Health Eating, and Tobacco-Use Prevention. 2000 Mar;1–230.  2. SHAPE America. Opportunity to Learn: Guidelines for Elementary, Middle & High School Physical Education - A Side‐by‐Side Comparison [Internet]. 2010 [cited 2020 Jul 5]. Available from: https://www.shapeamerica.org/standards/guidelines/upload/Opportunity-to-Learn-Grid.pdf  3. Mandigo J, Francis PDN, Lodewyk EDK. Position Paper Physical Literacy for Educators. 2009;13.  4. UNESCO. International Charter of Physical Education, Physical Activity and Sport [Internet]. 2015 [cited 2020 Jul 5]. Available from: https://unesdoc.unesco.org/ark:/48223/pf0000235409  5. UNESCO. Diretrizes em educação física de qualidade (EFQ) para gestores de políticas [Internet]. 2015 [cited 2020 Jul 5]. Available from: https://unesdoc.unesco.org/ark:/48223/pf0000231963 |
|  |  | 7 - PE teachers need adequate working conditions (n=5) | 1. SHAPE America. Opportunity to Learn: Guidelines for Elementary, Middle & High School Physical Education - A Side‐by‐Side Comparison [Internet]. 2010 [cited 2020 Jul 5]. Available from: https://www.shapeamerica.org/standards/guidelines/upload/Opportunity-to-Learn-Grid.pdf |
|  |  | 8 - PE spaces must have adequate facilities, such as bathrooms and water dispenser (n=5) | 1. SHAPE America. Opportunity to Learn: Guidelines for Elementary, Middle & High School Physical Education - A Side‐by‐Side Comparison [Internet]. 2010 [cited 2020 Jul 5]. Available from: https://www.shapeamerica.org/standards/guidelines/upload/Opportunity-to-Learn-Grid.pdf  2. The common Wealth. Enhancing the Contribution of Sport to the Sustainable Development Goals [Internet]. OECD Publishing; 2017 [cited 2020 Jul 5]. Available from: http://www.thecommonwealth-ilibrary.org/commonwealth/development/enhancing-the-contribution-of-sport-to-the-sustainable-development-goals_9781848599598-en  3. UNESCO. Diretrizes em educação física de qualidade (EFQ) para gestores de políticas [Internet]. 2015 [cited 2020 Jul 5]. Available from: https://unesdoc.unesco.org/ark:/48223/pf0000231963 |
|  | 4 - The government should allocate funding that supports PE as one of the components of education | 9 - The government should allocate funding that supports PE as one of the components of education (n=3) | 1. International Council of Sport Science and Physical EducationScience Education Policy. International Position Statement on Physical Education. 2010.  2. International Council of Sport Science and Physical EducationScience Education Policy. International Benchmarks for Physical Education Systems Developed by ICSSPE’s International Committee of Sport Pedagogy. 2012.  3. The common Wealth. Model indicators on sport, physical education and physical activity and the Sustainable Development Goals [Internet]. 2019 [cited 2020 Jul 5]. Available from: https://thecommonwealth.org/sites/default/files/inline/Sport%20and%20SDG%20Indicators%20v3.1.pdf  4. UNESCO. Diretrizes em educação física de qualidade (EFQ) para gestores de políticas [Internet]. 2015 [cited 2020 Jul 5]. Available from: https://unesdoc.unesco.org/ark:/48223/pf0000231963 |
|  | 5 - Creating safe environments for PE with emergency protocols | 10 - Creating safe environments for PE with emergency protocols (n=3) | 1. SHAPE America. Opportunity to Learn: Guidelines for Elementary, Middle & High School Physical Education - A Side‐by‐Side Comparison [Internet]. 2010 [cited 2020 Jul 5]. Available from: https://www.shapeamerica.org/standards/guidelines/upload/Opportunity-to-Learn-Grid.pdf  2. European Physical Education Association. European Framework of Quality Physical Education [Internet]. 2018 [cited 2020 Jul 5]. Available from: http://www.eupea.com/wp-content/uploads/2018/02/European-Framework-of-Quality-PE.pdf |
| Higher frequency and duration of PE classes and PA at school | 6 - More PE classes and longer class time | 11 - Offer PE classes at least 2x/week or 120 min/week for elementary school and 180 min/week for high school (n=13) | 1. Bogden JF. Fit, Healthy, and Ready To Learn: A School Health Policy Guide. Part I: Physical Activity, Health Eating, and Tobacco-Use Prevention. 2000 Mar;1–230.  2. National Association for Sport and Physical Education. A Position Statement from the National Association for Sport and Physical Education: Comprehensive School Physical Activity Program. Strategies: A Journal for Physical and Sport Educators. 2008 Jul;21(6):29–33.  3. Pate RR, Davis MG, Robinson TN, Stone EJ, McKenzie TL, Young JC. Promoting physical activity in children and youth: A leadership role for schools - A scientific statement from the American Heart Association Council on Nutrition, Physical Activity, and Metabolism (Physical Activity Committee) in collaboration with the Councils on Cardiovascular Disease in the Young and Cardiovascular Nursing. Circulation. 2006;114(11):1214–24.  4. Lee SM. School health guidelines to promote healthy eating and physical activity. Morbidity and Mortality Weekly Report. 2011;60(RR-5):1–78.  5. SHAPE America-Society of Health and Physical Educators. The essential components of physical education. Author Reston, VA; 2015.  6. SHAPE America. Shape of the Nation Status of Physical Education in the USA [Internet]. 2016 [cited 2020 Jul 5]. Available from: https://www.shapeamerica.org/advocacy/son/2016/upload/Shape-of-the-Nation-2016_web.pdf  7. SHAPE America. Opportunity to Learn: Guidelines for Elementary, Middle & High School Physical Education - A Side‐by‐Side Comparison [Internet]. 2010 [cited 2020 Jul 5]. Available from: https://www.shapeamerica.org/standards/guidelines/upload/Opportunity-to-Learn-Grid.pdf  8. SHAPE America. Guide for the Physical Education Policy [Internet]. 2016 [cited 2020 Jul 5]. Available from: https://www.shapeamerica.org/advocacy/upload/Guide-for-Physical-Education-Policy-9-23-14.pdf  9. Hayman LL, Williams CL, Daniels SR, Steinberger J, Paridon S, Dennison BA, et al. Cardiovascular health promotion in the schools: a statement for health and education professionals and child health advocates from the Committee on Atherosclerosis, Hypertension, and Obesity in Youth (AHOY) of the Council on Cardiovascular Disease in the Young, American Heart Association. Circulation. 2004/10/13 ed. 2004 Oct 12;110(15):2266–75.  10. Rütten A, Pfeifer K, Banzer W, Ferrari N, Füzéki E, Geidl W, et al. National Recommendations for Physical Activity and Physical Activity Promotion [Internet]. 2016 [cited 2020 Jul 3]. Available from: https://opus4.kobv.de/opus4-fau/frontdoor/index/index/docId/7827  11. American Heart Association. Increasing and Improving Physical Education and Physical Activity in Schools: Benefits for Children’s Health and Educational Outcomes [Internet]. 2015 [cited 2020 Jul 5]. Available from: https://www.heart.org/idc/groups/heart-public/@wcm/@adv/documents/downloadable/ucm_473782.pdf  12. Mandigo J, Francis PDN, Lodewyk EDK. Position Paper Physical Literacy for Educators. 2009;13.  13. Australian 24-Hour Movement Guidelines for Children (5-12 years) and Young People (13-17 years): An Integration of Physical Activity, Sedentary Behaviour, and Sleep. :148.  14. OLIVEIRA ARC de, SARTORI SK, LAURINDO E. Recomendações para a Educação Física escolar. Sistema CONFEF/CREFs Conselhos Federal e Regionais de Educação Física. 2014;  15. Lloyd RS, Faigenbaum AD, Stone MH, Oliver JL, Jeffreys I, Moody JA, et al. Position statement on youth resistance training: the 2014 International Consensus. British Journal of Sports Medicine. 2014;48(7):498–505.  16. U.S. Department of Health and Human Services. 2018 Physical Activity Guidelines Advisory Committee [Internet]. 2018 [cited 2020 Jul 5]. Available from: https://health.gov/sites/default/files/2019-09/PAG_Advisory_Committee_Report.pdf  17. Centers for Disease Control and Prevention. The Association Between School-Based Physical Activity, Including Physical Education, and Academic Performance. 2010.  18. The common Wealth. Model indicators on sport, physical education and physical activity and the Sustainable Development Goals [Internet]. 2019 [cited 2020 Jul 5]. Available from: https://thecommonwealth.org/sites/default/files/inline/Sport%20and%20SDG%20Indicators%20v3.1.pdf  19. UNESCO. Diretrizes em educação física de qualidade (EFQ) para gestores de políticas [Internet]. 2015 [cited 2020 Jul 5]. Available from: https://unesdoc.unesco.org/ark:/48223/pf0000231963  20. The common Wealth. Measuring the contribution of sport, physical education and physical activity to the Sustainable Development Goals Toolkit and model indicators [Internet]. 2019 [cited 2020 Jul 5]. Available from: https://thecommonwealth.org/sites/default/files/inline/Sport-SDGs-Indicator-Framework.pdf  21. Centers for Disease Control and Prevention. School Health Index: A Self-Assessment and Planning Guide Elementary School 2017 [Internet]. 2017 [cited 2020 Jul 5]. Available from: https://www.cdc.gov/healthyschools/shi/pdf/Elementary-Total-2017.pdf  22. Centers for Disease Control and Prevention. School Health Index: A Self-Assessment and Planning Guide Middle and High School 2017 [Internet]. 2017 [cited 2020 Jul 5]. Available from: https://www.cdc.gov/healthyschools/shi/pdf/Middle-High-Total-2017.pdf |
|  | 7 - More PA in the school environment | 12 - Implementation of actions to increase PA in school, including in PE (n=8) | 1. Buscemi J, Kong A, Fitzgibbon ML, Bustamante EE, Davis CL, Pate RR, et al. Society of Behavioral Medicine position statement: elementary school-based physical activity supports academic achievement. Translational behavioral medicine. 2015/01/15 ed. 2014 Dec;4(4):436–8.  2. Association for Physical Education. Health Position Paper afPE 2020 [Internet]. 2020 [cited 2020 Jul 5]. Available from: https://www.afpe.org.uk/physical-education/wp-content/uploads/Health-Position-Paper-2020-Web.pdf  3. American Heart Association. Increasing and Improving Physical Education and Physical Activity in Schools: Benefits for Children’s Health and Educational Outcomes [Internet]. 2015 [cited 2020 Jul 5]. Available from: https://www.heart.org/idc/groups/heart-public/@wcm/@adv/documents/downloadable/ucm_473782.pdf  4. Increasing physical activity. A report on recommendations of the Task Force on Community Preventive Services. MMWR Recommendations and reports : Morbidity and mortality weekly report Recommendations and reports. 2001/11/09 ed. 2001 Oct 26;50(Rr-18):1–14.  5. The common Wealth. Measuring the contribution of sport, physical education and physical activity to the Sustainable Development Goals Toolkit and model indicators [Internet]. 2019 [cited 2020 Jul 5]. Available from: https://thecommonwealth.org/sites/default/files/inline/Sport-SDGs-Indicator-Framework.pdf  6. Department of Health (Ireland). Get Ireland Active! The National Physical Activity Plan for Ireland [Internet]. 2016 [cited 2020 Jul 5]. Available from: https://assets.gov.ie/12198/5f3dbab207f2464bba3b9b3f6d02bff6.pdf |
| The need for PE classes to be inclusive and never punitive | 8 - PE (or lack thereof) should not be a punishment | 13 - Participation in PE (or non-participation) should not work as a punishment policy (n=2) | 1. Bogden JF. Fit, Healthy, and Ready To Learn: A School Health Policy Guide. Part I: Physical Activity, Health Eating, and Tobacco-Use Prevention. 2000 Mar;1–230.  2. Ecuador. Ministerio de salud pública Del Ecuador, Coordinación Nacional de Nutrición. Guia de Actividad Física dirigida al personal de salud II. 2011.  3. SHAPE America-Society of Health and Physical Educators. The essential components of physical education. Author Reston, VA; 2015.  4. SHAPE America. Shape of the Nation Status of Physical Education in the USA [Internet]. 2016 [cited 2020 Jul 5]. Available from: https://www.shapeamerica.org/advocacy/son/2016/upload/Shape-of-the-Nation-2016_web.pdf  5. SHAPE America. Opportunity to Learn: Guidelines for Elementary, Middle & High School Physical Education - A Side‐by‐Side Comparison [Internet]. 2010 [cited 2020 Jul 5]. Available from: https://www.shapeamerica.org/standards/guidelines/upload/Opportunity-to-Learn-Grid.pdf  6. SHAPE America. Getting to Know Your Child’s PE Program: A Parent’s Guide [Internet]. 2019 [cited 2020 Jul 5]. Available from: https://www.shapeamerica.org/uploads/pdfs/2017/downloads/eguides/Parent_Checklist.pdf  7. Rütten A, Pfeifer K, Banzer W, Ferrari N, Füzéki E, Geidl W, et al. National Recommendations for Physical Activity and Physical Activity Promotion [Internet]. 2016 [cited 2020 Jul 3]. Available from: https://opus4.kobv.de/opus4-fau/frontdoor/index/index/docId/7827  8. Centers for Disease Control and Prevention. School Health Index: A Self-Assessment and Planning Guide Elementary School 2017 [Internet]. 2017 [cited 2020 Jul 5]. Available from: https://www.cdc.gov/healthyschools/shi/pdf/Elementary-Total-2017.pdf  9. Centers for Disease Control and Prevention. School Health Index: A Self-Assessment and Planning Guide Middle and High School 2017 [Internet]. 2017 [cited 2020 Jul 5]. Available from: https://www.cdc.gov/healthyschools/shi/pdf/Middle-High-Total-2017.pdf |
|  | 9 - Policies for exemption toward participation in PE classes | 14 - Exemptions should only be allowed if there is a risk to health or due to the impediment of religion (n=4) | 1. Bogden JF. Fit, Healthy, and Ready To Learn: A School Health Policy Guide. Part I: Physical Activity, Health Eating, and Tobacco-Use Prevention. 2000 Mar;1–230.  2. SHAPE America. Guide for the Physical Education Policy [Internet]. 2016 [cited 2020 Jul 5]. Available from: https://www.shapeamerica.org/advocacy/upload/Guide-for-Physical-Education-Policy-9-23-14.pdf  3. Lee SM. School health guidelines to promote healthy eating and physical activity. Morbidity and Mortality Weekly Report. 2011;60(RR-5):1–78.  4. SHAPE America-Society of Health and Physical Educators. The essential components of physical education. Author Reston, VA; 2015.  5. SHAPE America. Shape of the Nation Status of Physical Education in the USA [Internet]. 2016 [cited 2020 Jul 5]. Available from: https://www.shapeamerica.org/advocacy/son/2016/upload/Shape-of-the-Nation-2016_web.pdf  6. SHAPE America. Opportunity to Learn: Guidelines for Elementary, Middle & High School Physical Education - A Side‐by‐Side Comparison [Internet]. 2010 [cited 2020 Jul 5]. Available from: https://www.shapeamerica.org/standards/guidelines/upload/Opportunity-to-Learn-Grid.pdf  7. SHAPE America. Physical Education is Essential for All Students: No Substitutions, Waivers or Exemptions for Physical Education. 2018;6.  8. SHAPE America. Getting to Know Your Child’s PE Program: A Parent’s Guide [Internet]. 2019 [cited 2020 Jul 5]. Available from: https://www.shapeamerica.org/uploads/pdfs/2017/downloads/eguides/Parent_Checklist.pdf |
|  | 10 - Implement actions for the inclusion of all students in PE classes | 15 - Implement actions for the inclusion of all students in PE classes (n=5) | 1. Rütten A, Pfeifer K, Banzer W, Ferrari N, Füzéki E, Geidl W, et al. National Recommendations for Physical Activity and Physical Activity Promotion [Internet]. 2016 [cited 2020 Jul 3]. Available from: https://opus4.kobv.de/opus4-fau/frontdoor/index/index/docId/7827  2. American Heart Association. Increasing and Improving Physical Education and Physical Activity in Schools: Benefits for Children’s Health and Educational Outcomes [Internet]. 2015 [cited 2020 Jul 5]. Available from: https://www.heart.org/idc/groups/heart-public/@wcm/@adv/documents/downloadable/ucm_473782.pdf  3. The common Wealth. Measuring the contribution of sport, physical education and physical activity to the Sustainable Development Goals Toolkit and model indicators [Internet]. 2019 [cited 2020 Jul 5]. Available from: https://thecommonwealth.org/sites/default/files/inline/Sport-SDGs-Indicator-Framework.pdf  4. UNESCO. International Charter of Physical Education, Physical Activity and Sport [Internet]. 2015 [cited 2020 Jul 5]. Available from: https://unesdoc.unesco.org/ark:/48223/pf0000235409  5. UNESCO. Diretrizes em educação física de qualidade (EFQ) para gestores de políticas [Internet]. 2015 [cited 2020 Jul 5]. Available from: https://unesdoc.unesco.org/ark:/48223/pf0000231963 |
| Mandatory daily PE at all levels of education to promote student health and well-being | 11 - PE as a fundamental component for students' health and well-being throughout their lives | 16 - Promotion of school policies that recognize PE as a context for promoting health and well-being (n=6) | 1. Ministry of Social Affairs and Health. Recommendations for the promotion of physical activity in Finland. 2010.  2. Ministerio de la Protección Social. Guía para el desarrollo de programas intersectoriales y comunitarios para la promoción de la actividad física: programa nacional de actividad física Colombia activa y saludable [Internet]. Imprenta Nacional de Colombia; 2009. Available from: https://books.google.com.br/books?id=MWiAswEACAAJ  3. Costa Rica. Ministerio de Salud y Ministerio de Deporte y Recreación. Plan Nacional de Actividad Física y Salud 2011-2021. 2011.  4. International Council of Sport Science and Physical EducationScience Education Policy. International Position Statement on Physical Education. 2010.  5. SHAPE America. Physical Education is Essential for All Students: No Substitutions, Waivers or Exemptions for Physical Education. 2018;6.  6. World Health Organization. Global action plan on physical activity 2018–2030: more active people for a healthier world [Internet]. 2018 [cited 2020 Jul 5]. Available from: https://www.who.int/ncds/prevention/physical-activity/global-action-plan-2018-2030/en/  7. International Council of Sport Science and Physical EducationScience Education Policy. International Benchmarks for Physical Education Systems Developed by ICSSPE’s International Committee of Sport Pedagogy. 2012. |
|  |  | 17 - Implementation of educational campaigns on the importance of PE (n=1) | 1. UNESCO. Diretrizes em educação física de qualidade (EFQ) para gestores de políticas [Internet]. 2015 [cited 2020 Jul 5]. Available from: https://unesdoc.unesco.org/ark:/48223/pf0000231963 |
|  | 12 - Mandatory daily PE at all levels of education | 18 - Mandatory daily and quality PE (n=5) | 1. Centers for Disease Control and Prevention. A CDC Review of School Laws and Policies Concerning Child and Adolescent Health. Journal of School Health. 2008 Feb;78(2):69–128.  2. Bogden JF. Fit, Healthy, and Ready To Learn: A School Health Policy Guide. Part I: Physical Activity, Health Eating, and Tobacco-Use Prevention. 2000 Mar;1–230.  3. National Association for Sport and Physical Education. A Position Statement from the National Association for Sport and Physical Education: Comprehensive School Physical Activity Program. Strategies: A Journal for Physical and Sport Educators. 2008 Jul;21(6):29–33.  4. ParticipACTION Advisory Groups. Active Canada 20/20: A physical activity strategy and change agenda for Canada. 2012.  5. Mandigo J, Francis PDN, Lodewyk EDK. Position Paper Physical Literacy for Educators. 2009;13.  6. Pate RR, Davis MG, Robinson TN, Stone EJ, McKenzie TL, Young JC. Promoting physical activity in children and youth: A leadership role for schools - A scientific statement from the American Heart Association Council on Nutrition, Physical Activity, and Metabolism (Physical Activity Committee) in collaboration with the Councils on Cardiovascular Disease in the Young and Cardiovascular Nursing. Circulation. 2006;114(11):1214–24.  7. Increasing physical activity. A report on recommendations of the Task Force on Community Preventive Services. MMWR Recommendations and reports : Morbidity and mortality weekly report Recommendations and reports. 2001/11/09 ed. 2001 Oct 26;50(Rr-18):1–14.  8. Lee SM. School health guidelines to promote healthy eating and physical activity. Morbidity and Mortality Weekly Report. 2011;60(RR-5):1–78.  9. Physical education statement by the President’s Council on Physical Fitness and Sports. NASNewsletter. 2002/05/04 ed. 2001 Jan;16(1):13.  10. The Australian Council for Health, Physical Education and Recreation (ACHPER). ACHPER NATIONAL POSITION STATEMENT: Support of the Australian Curriculum: Health and Physical Education [Internet]. 2017 [cited 2020 Jul 13]. Available from: https://www.achper.org.au/documents/item/393  11. Ecuador. Ministerio de salud pública Del Ecuador, Coordinación Nacional de Nutrición. Guia de Actividad Física dirigida al personal de salud II. 2011.  12. UNESCO. Declaration of Berlin: International Conference of Ministers and Senior Officials Responsible for Physical Education and Sport [Internet]. 2013 [cited 2020 Jul 5]. Available from: https://unesdoc.unesco.org/ark:/48223/pf0000221114  13. UNESCO. International Charter of Physical Education, Physical Activity and Sport [Internet]. 2015 [cited 2020 Jul 5]. Available from: https://unesdoc.unesco.org/ark:/48223/pf0000235409  14. Council on Sports Medicine and Fitness and Council on School Health. Active healthy living: prevention of childhood obesity through increased physical activity. Pediatrics. 2006/05/03 ed. 2006 May;117(5):1834–42.  15. SHAPE America-Society of Health and Physical Educators. The essential components of physical education. Author Reston, VA; 2015.  16. OLIVEIRA ARC de, SARTORI SK, LAURINDO E. Recomendações para a Educação Física escolar. Sistema CONFEF/CREFs Conselhos Federal e Regionais de Educação Física. 2014;  17. International Council of Sport Science and Physical EducationScience Education Policy. International Position Statement on Physical Education. 2010.  18. The common Wealth. Model indicators on sport, physical education and physical activity and the Sustainable Development Goals [Internet]. 2019 [cited 2020 Jul 5]. Available from: https://thecommonwealth.org/sites/default/files/inline/Sport%20and%20SDG%20Indicators%20v3.1.pdf  19. UNESCO. Diretrizes em educação física de qualidade (EFQ) para gestores de políticas [Internet]. 2015 [cited 2020 Jul 5]. Available from: https://unesdoc.unesco.org/ark:/48223/pf0000231963  20. International Council of Sport Science and Physical EducationScience Education Policy. International Benchmarks for Physical Education Systems Developed by ICSSPE’s International Committee of Sport Pedagogy. 2012.  21. SHAPE America. Shape of the Nation Status of Physical Education in the USA [Internet]. 2016 [cited 2020 Jul 5]. Available from: https://www.shapeamerica.org/advocacy/son/2016/upload/Shape-of-the-Nation-2016_web.pdf  22. SHAPE America. Guide for the Physical Education Policy [Internet]. 2016 [cited 2020 Jul 5]. Available from: https://www.shapeamerica.org/advocacy/upload/Guide-for-Physical-Education-Policy-9-23-14.pdf  23. Federation Internationale d´Éducation Physique. The World Manifest of Physical Education FIEP 2000 [Internet]. 2000 [cited 2020 Jul 5]. Available from: http://fiepeurope.eu/manifest.php |
|  |  | 19 - PE must be inserted in early childhood education (n=3) | 1. OLIVEIRA ARC de, SARTORI SK, LAURINDO E. Recomendações para a Educação Física escolar. Sistema CONFEF/CREFs Conselhos Federal e Regionais de Educação Física. 2014;  2. Ministerio del Deporte, Gobierno de Chile. Política Nacional de Actividad Física y Deporte 2016-2025 [Internet]. 2016 [cited 2020 Jul 2]. Available from: http://www.mindep.cl/wp-content/uploads/2015/05/POLITICA-ULTIMA-VERSI%C3%93N-021116.pdf |
| Evaluation of the implementation of PE guidelines at school | 13 - Evaluation of the implementation of PE guidelines at school | 20 - Evaluation of the implementation of PE guidelines at school (n=6) | 1. The Australian Council for Health, Physical Education and Recreation (ACHPER). ACHPER NATIONAL POSITION STATEMENT: Support of the Australian Curriculum: Health and Physical Education [Internet]. 2017 [cited 2020 Jul 13]. Available from: https://www.achper.org.au/documents/item/393  2. International Council of Sport Science and Physical EducationScience Education Policy. International Position Statement on Physical Education. 2010.  3. International Council of Sport Science and Physical EducationScience Education Policy. International Benchmarks for Physical Education Systems Developed by ICSSPE’s International Committee of Sport Pedagogy. 2012.  4. SHAPE America. Getting to Know Your Child’s PE Program: A Parent’s Guide [Internet]. 2019 [cited 2020 Jul 5]. Available from: https://www.shapeamerica.org/uploads/pdfs/2017/downloads/eguides/Parent_Checklist.pdf  5. The common Wealth. Enhancing the Contribution of Sport to the Sustainable Development Goals [Internet]. OECD Publishing; 2017 [cited 2020 Jul 5]. Available from: http://www.thecommonwealth-ilibrary.org/commonwealth/development/enhancing-the-contribution-of-sport-to-the-sustainable-development-goals_9781848599598-en  6. The common Wealth. Model indicators on sport, physical education and physical activity and the Sustainable Development Goals [Internet]. 2019 [cited 2020 Jul 5]. Available from: https://thecommonwealth.org/sites/default/files/inline/Sport%20and%20SDG%20Indicators%20v3.1.pdf  7. UNESCO. Diretrizes em educação física de qualidade (EFQ) para gestores de políticas [Internet]. 2015 [cited 2020 Jul 5]. Available from: https://unesdoc.unesco.org/ark:/48223/pf0000231963 |
| Certified, trained, qualified teachers and advocates of PE | 14 - The teacher must present himself as an advocate and leader of PE in the school, aiming to improve teaching-learning | 21 - The teacher must be an advocate and leader of PE in school, aiming to improve the teaching-learning process (n=3) | 1. SHAPE America. Opportunity to Learn: Guidelines for Elementary, Middle & High School Physical Education - A Side‐by‐Side Comparison [Internet]. 2010 [cited 2020 Jul 5]. Available from: https://www.shapeamerica.org/standards/guidelines/upload/Opportunity-to-Learn-Grid.pdf |
|  | 15 - Certified, trained and qualified teachers | 22 - Inclusion of certified and qualified teachers, in regular training (n=10) | 1. Costa Rica. Ministerio de Salud y Ministerio de Deporte y Recreación. Plan Nacional de Actividad Física y Salud 2011-2021. 2011.  2. Rütten A, Pfeifer K, Banzer W, Ferrari N, Füzéki E, Geidl W, et al. National Recommendations for Physical Activity and Physical Activity Promotion [Internet]. 2016 [cited 2020 Jul 3]. Available from: https://opus4.kobv.de/opus4-fau/frontdoor/index/index/docId/7827  3. International Council of Sport Science and Physical EducationScience Education Policy. International Position Statement on Physical Education. 2010.  4. Kay T, Dudfield O, Commonwealth Secretariat. The commonwealth guide to advancing development through sport. London: Commonwealth Secretariat; 2013.  5. International Council of Sport Science and Physical EducationScience Education Policy. International Benchmarks for Physical Education Systems Developed by ICSSPE’s International Committee of Sport Pedagogy. 2012.  6. Association for Supervision and Curriculum Development (ASCD). ASCD’s Position on the Whole Child [Internet]. 2020 [cited 2020 Jul 5]. Available from: http://files.ascd.org/pdfs/programs/WholeChildNetwork/2020-whole-child-network-learning-compact-renewed.pdf  7. Pate RR, Davis MG, Robinson TN, Stone EJ, McKenzie TL, Young JC. Promoting physical activity in children and youth: A leadership role for schools - A scientific statement from the American Heart Association Council on Nutrition, Physical Activity, and Metabolism (Physical Activity Committee) in collaboration with the Councils on Cardiovascular Disease in the Young and Cardiovascular Nursing. Circulation. 2006;114(11):1214–24.  8. SHAPE America. Shape of the Nation Status of Physical Education in the USA [Internet]. 2016 [cited 2020 Jul 5]. Available from: https://www.shapeamerica.org/advocacy/son/2016/upload/Shape-of-the-Nation-2016_web.pdf  9. SHAPE America. Guide for the Physical Education Policy [Internet]. 2016 [cited 2020 Jul 5]. Available from: https://www.shapeamerica.org/advocacy/upload/Guide-for-Physical-Education-Policy-9-23-14.pdf  10. Buscemi J, Kong A, Fitzgibbon ML, Bustamante EE, Davis CL, Pate RR, et al. Society of Behavioral Medicine position statement: elementary school-based physical activity supports academic achievement. Translational behavioral medicine. 2015/01/15 ed. 2014 Dec;4(4):436–8.  11. SHAPE America. Opportunity to Learn: Guidelines for Elementary, Middle & High School Physical Education - A Side‐by‐Side Comparison [Internet]. 2010 [cited 2020 Jul 5]. Available from: https://www.shapeamerica.org/standards/guidelines/upload/Opportunity-to-Learn-Grid.pdf  12. Lee SM. School health guidelines to promote healthy eating and physical activity. Morbidity and Mortality Weekly Report. 2011;60(RR-5):1–78.  13. SHAPE America. Getting to Know Your Child’s PE Program: A Parent’s Guide [Internet]. 2019 [cited 2020 Jul 5]. Available from: https://www.shapeamerica.org/uploads/pdfs/2017/downloads/eguides/Parent_Checklist.pdf  14. U.S. Department of Health and Human Services. 2018 Physical Activity Guidelines Advisory Committee [Internet]. 2018 [cited 2020 Jul 5]. Available from: https://health.gov/sites/default/files/2019-09/PAG_Advisory_Committee_Report.pdf  15. American Heart Association. Increasing and Improving Physical Education and Physical Activity in Schools: Benefits for Children’s Health and Educational Outcomes [Internet]. 2015 [cited 2020 Jul 5]. Available from: https://www.heart.org/idc/groups/heart-public/@wcm/@adv/documents/downloadable/ucm_473782.pdf  16. European Physical Education Association. European Framework of Quality Physical Education [Internet]. 2018 [cited 2020 Jul 5]. Available from: http://www.eupea.com/wp-content/uploads/2018/02/European-Framework-of-Quality-PE.pdf  17. UNESCO. Declaration of Berlin: International Conference of Ministers and Senior Officials Responsible for Physical Education and Sport [Internet]. 2013 [cited 2020 Jul 5]. Available from: https://unesdoc.unesco.org/ark:/48223/pf0000221114  18. The common Wealth. Enhancing the Contribution of Sport to the Sustainable Development Goals [Internet]. OECD Publishing; 2017 [cited 2020 Jul 5]. Available from: http://www.thecommonwealth-ilibrary.org/commonwealth/development/enhancing-the-contribution-of-sport-to-the-sustainable-development-goals_9781848599598-en |
|  |  | 23 - Training for teachers on PA and health topics (n=1) | 1. Ministerio del Deporte, Gobierno de Chile. Política Nacional de Actividad Física y Deporte 2016-2025 [Internet]. 2016 [cited 2020 Jul 2]. Available from: http://www.mindep.cl/wp-content/uploads/2015/05/POLITICA-ULTIMA-VERSI%C3%93N-021116.pdf |
|  |  | 24 - The University has an essential role in the training of PE teachers (n=2) | 1. Ministerio del Deporte, Gobierno de Chile. Política Nacional de Actividad Física y Deporte 2016-2025 [Internet]. 2016 [cited 2020 Jul 2]. Available from: http://www.mindep.cl/wp-content/uploads/2015/05/POLITICA-ULTIMA-VERSI%C3%93N-021116.pdf  2. The Australian Council for Health, Physical Education and Recreation (ACHPER). ACHPER NATIONAL POSITION STATEMENT: Support of the Australian Curriculum: Health and Physical Education [Internet]. 2017 [cited 2020 Jul 13]. Available from: https://www.achper.org.au/documents/item/393  3. ParticipACTION Advisory Groups. Active Canada 20/20: A physical activity strategy and change agenda for Canada. 2012.  4. The common Wealth. Measuring the contribution of sport, physical education and physical activity to the Sustainable Development Goals Toolkit and model indicators [Internet]. 2019 [cited 2020 Jul 5]. Available from: https://thecommonwealth.org/sites/default/files/inline/Sport-SDGs-Indicator-Framework.pdf  5. UNESCO. Diretrizes em educação física de qualidade (EFQ) para gestores de políticas [Internet]. 2015 [cited 2020 Jul 5]. Available from: https://unesdoc.unesco.org/ark:/48223/pf0000231963 |

| **CURRICULUM** | | | |
| --- | --- | --- | --- |
| **Recommendations** | | | **References** |
| The curriculum must follow national, state, and municipal standards with a sequential and progressive structure | 1 - The curriculum structure should be sequential and progressive | 1 - The curriculum structure should be sequential and progressive (n=5) | 1. Centers for Disease Control and Prevention. A CDC Review of School Laws and Policies Concerning Child and Adolescent Health. Journal of School Health. 2008 Feb;78(2):69–128.  2. OLIVEIRA ARC de, SARTORI SK, LAURINDO E. Recomendações para a Educação Física escolar. Sistema CONFEF/CREFs Conselhos Federal e Regionais de Educação Física. 2014;  3. Bogden JF. Fit, Healthy, and Ready To Learn: A School Health Policy Guide. Part I: Physical Activity, Health Eating, and Tobacco-Use Prevention. 2000 Mar;1–230.  4. UNESCO. Diretrizes em educação física de qualidade (EFQ) para gestores de políticas [Internet]. 2015 [cited 2020 Jul 5]. Available from: https://unesdoc.unesco.org/ark:/48223/pf0000231963  5. Mandigo J, Francis PDN, Lodewyk EDK. Position Paper Physical Literacy for Educators. 2009;13.  6. SHAPE America. Appropriate Instructional Practice Guidelines, K-12: A Side-by-Side Comparison SHAPE America – Society of Health and Physical Educators [Internet]. 2009 [cited 2020 Jul 5]. Available from: https://www.shapeamerica.org/upload/Appropriate-Instructional-Practice-Guidelines-K-12.pdf  7. The common Wealth. Model indicators on sport, physical education and physical activity and the Sustainable Development Goals [Internet]. 2019 [cited 2020 Jul 5]. Available from: https://thecommonwealth.org/sites/default/files/inline/Sport%20and%20SDG%20Indicators%20v3.1.pdf  8. Lee SM. School health guidelines to promote healthy eating and physical activity. Morbidity and Mortality Weekly Report. 2011;60(RR-5):1–78.  9. SHAPE America. Opportunity to Learn: Guidelines for Elementary, Middle & High School Physical Education - A Side‐by‐Side Comparison [Internet]. 2010 [cited 2020 Jul 5]. Available from: https://www.shapeamerica.org/standards/guidelines/upload/Opportunity-to-Learn-Grid.pdf  10. SHAPE America-Society of Health and Physical Educators. The essential components of physical education. Author Reston, VA; 2015.  11. SHAPE America. Shape of the Nation Status of Physical Education in the USA [Internet]. 2016 [cited 2020 Jul 5]. Available from: https://www.shapeamerica.org/advocacy/son/2016/upload/Shape-of-the-Nation-2016_web.pdf |
|  | 2 - The curriculum must follow national, state, and municipal standards | 2 - The curriculum must follow national, state, and municipal standards (n=3) | 1. National Association for Sport and Physical Education. A Position Statement from the National Association for Sport and Physical Education: Comprehensive School Physical Activity Program. Strategies: A Journal for Physical and Sport Educators. 2008 Jul;21(6):29–33.  2. Bogden JF. Fit, Healthy, and Ready To Learn: A School Health Policy Guide. Part I: Physical Activity, Health Eating, and Tobacco-Use Prevention. 2000 Mar;1–230.  3. SHAPE America-Society of Health and Physical Educators. The essential components of physical education. Author Reston, VA; 2015.  4. SHAPE America. Shape of the Nation Status of Physical Education in the USA [Internet]. 2016 [cited 2020 Jul 5]. Available from: https://www.shapeamerica.org/advocacy/son/2016/upload/Shape-of-the-Nation-2016_web.pdf  5. SHAPE America. Opportunity to Learn: Guidelines for Elementary, Middle & High School Physical Education - A Side‐by‐Side Comparison [Internet]. 2010 [cited 2020 Jul 5]. Available from: https://www.shapeamerica.org/standards/guidelines/upload/Opportunity-to-Learn-Grid.pdf |
|  | 3 - Inclusion of academic standards for PE | 3 - Inclusion of academic standards for PE (n=3) | 1. SHAPE America. Guide for the Physical Education Policy [Internet]. 2016 [cited 2020 Jul 5]. Available from: https://www.shapeamerica.org/advocacy/upload/Guide-for-Physical-Education-Policy-9-23-14.pdf  2. SHAPE America. Physical Education is Essential for All Students: No Substitutions, Waivers or Exemptions for Physical Education. 2018;6.  3. SHAPE America. Opportunity to Learn: Guidelines for Elementary, Middle & High School Physical Education - A Side‐by‐Side Comparison [Internet]. 2010 [cited 2020 Jul 5]. Available from: https://www.shapeamerica.org/standards/guidelines/upload/Opportunity-to-Learn-Grid.pdf |
| The need for the inclusion of movement behaviors content that promotes physical, cognitive, and social literacy throughout life | 4 - Inclusion of movement behaviors content that encourage the practice of PA throughout life | 4 - Inclusion of movement behaviors content that encourage the practice of PA throughout life (n=10) | 1. Bogden JF. Fit, Healthy, and Ready To Learn: A School Health Policy Guide. Part I: Physical Activity, Health Eating, and Tobacco-Use Prevention. 2000 Mar;1–230.  2. Ministry of Social Affairs and Health. Recommendations for the promotion of physical activity in Finland. 2010.  3. ParticipACTION Advisory Groups. Active Canada 20/20: A physical activity strategy and change agenda for Canada. 2012.  4. SHAPE America. Appropriate Instructional Practice Guidelines, K-12: A Side-by-Side Comparison SHAPE America – Society of Health and Physical Educators [Internet]. 2009 [cited 2020 Jul 5]. Available from: https://www.shapeamerica.org/upload/Appropriate-Instructional-Practice-Guidelines-K-12.pdf  5. The common Wealth. Model indicators on sport, physical education and physical activity and the Sustainable Development Goals [Internet]. 2019 [cited 2020 Jul 5]. Available from: https://thecommonwealth.org/sites/default/files/inline/Sport%20and%20SDG%20Indicators%20v3.1.pdf  6. UNESCO. Diretrizes em educação física de qualidade (EFQ) para gestores de políticas [Internet]. 2015 [cited 2020 Jul 5]. Available from: https://unesdoc.unesco.org/ark:/48223/pf0000231963  7. Council on Sports Medicine and Fitness and Council on School Health. Active healthy living: prevention of childhood obesity through increased physical activity. Pediatrics. 2006/05/03 ed. 2006 May;117(5):1834–42.  8. SHAPE America. Opportunity to Learn: Guidelines for Elementary, Middle & High School Physical Education - A Side‐by‐Side Comparison [Internet]. 2010 [cited 2020 Jul 5]. Available from: https://www.shapeamerica.org/standards/guidelines/upload/Opportunity-to-Learn-Grid.pdf  9. OLIVEIRA ARC de, SARTORI SK, LAURINDO E. Recomendações para a Educação Física escolar. Sistema CONFEF/CREFs Conselhos Federal e Regionais de Educação Física. 2014;  10. (17) (PDF) Global forum for physical education pedagogy 2010 (GoFPEP 2010) statement of consensus:: pedagogy of health and physical education in the xxith century [Internet]. ResearchGate. [cited 2020 Jun 17]. Available from: https://www.researchgate.net/publication/317462682_Global_forum_for_physical_education_pedagogy_2010_GoFPEP_2010_statement_of_consensus_pedagogy_of_health_and_physical_education_in_the_xxith_century  11. Lee SM. School health guidelines to promote healthy eating and physical activity. Morbidity and Mortality Weekly Report. 2011;60(RR-5):1–78.  12. Ecuador. Ministerio de salud pública Del Ecuador, Coordinación Nacional de Nutrición. Guia de Actividad Física dirigida al personal de salud II. 2011.  13. Costa Rica. Ministerio de Salud y Ministerio de Deporte y Recreación. Plan Nacional de Actividad Física y Salud 2011-2021. 2011.  14. European Physical Education Association. European Framework of Quality Physical Education [Internet]. 2018 [cited 2020 Jul 5]. Available from: http://www.eupea.com/wp-content/uploads/2018/02/European-Framework-of-Quality-PE.pdf  15. The common Wealth. Measuring the contribution of sport, physical education and physical activity to the Sustainable Development Goals Toolkit and model indicators [Internet]. 2019 [cited 2020 Jul 5]. Available from: https://thecommonwealth.org/sites/default/files/inline/Sport-SDGs-Indicator-Framework.pdf  16. Association for Physical Education. Health Position Paper afPE 2020 [Internet]. 2020 [cited 2020 Jul 5]. Available from: https://www.afpe.org.uk/physical-education/wp-content/uploads/Health-Position-Paper-2020-Web.pdf |
|  | 5 - Inclusion of content that promotes physical, cognitive, and social literacy | 5 - Inclusion of content that promotes physical, cognitive, and social literacy (n=4) | 1. Centers for Disease Control and Prevention. A CDC Review of School Laws and Policies Concerning Child and Adolescent Health. Journal of School Health. 2008 Feb;78(2):69–128.  2. OLIVEIRA ARC de, SARTORI SK, LAURINDO E. Recomendações para a Educação Física escolar. Sistema CONFEF/CREFs Conselhos Federal e Regionais de Educação Física. 2014;  3. Mandigo J, Francis PDN, Lodewyk EDK. Position Paper Physical Literacy for Educators. 2009;13.  4. The common Wealth. Measuring the contribution of sport, physical education and physical activity to the Sustainable Development Goals Toolkit and model indicators [Internet]. 2019 [cited 2020 Jul 5]. Available from: https://thecommonwealth.org/sites/default/files/inline/Sport-SDGs-Indicator-Framework.pdf  5. The common Wealth. Enhancing the Contribution of Sport to the Sustainable Development Goals [Internet]. OECD Publishing; 2017 [cited 2020 Jul 5]. Available from: http://www.thecommonwealth-ilibrary.org/commonwealth/development/enhancing-the-contribution-of-sport-to-the-sustainable-development-goals_9781848599598-en  6. UNESCO. Diretrizes em educação física de qualidade (EFQ) para gestores de políticas [Internet]. 2015 [cited 2020 Jul 5]. Available from: https://unesdoc.unesco.org/ark:/48223/pf0000231963  7. Organisation for Economic Co-operation and Development (OECD). Making Physical Education Dynamic and Inclusive for 2030: International Curriculum Analysis [Internet]. 2019 [cited 2020 Jul 5]. Available from: https://www.oecd.org/education/2030-project/contact/OECD_FUTURE_OF_EDUCATION_2030_MAKING_PHYSICAL_DYNAMIC_AND_INCLUSIVE_FOR_2030.pdf  8. Association for Physical Education. Health Position Paper afPE 2020 [Internet]. 2020 [cited 2020 Jul 5]. Available from: https://www.afpe.org.uk/physical-education/wp-content/uploads/Health-Position-Paper-2020-Web.pdf |
| Inclusion of cross-cutting themes to the key concepts of PE | 6 - Inclusion of content that prevents injuries and violence in PE | 6 - Inclusion of content that prevents injuries and violence in PE (n=2) | 1. Barrios LC, Sleet DA, Mercy JA. CDC school health guidelines to prevent unintentional injuries and violence. American Journal of Health Education. 2003;34(5 Suppl):S-18-s-22. |
|  | 7 - Inclusion of cross-cutting themes to the key  concepts of PE in the curriculum | 7- Inclusion of cross-cutting themes to the key concepts of PE in the curriculum (n=4) | 1. OLIVEIRA ARC de, SARTORI SK, LAURINDO E. Recomendações para a Educação Física escolar. Sistema CONFEF/CREFs Conselhos Federal e Regionais de Educação Física. 2014;  2. Organisation for Economic Co-operation and Development (OECD). Making Physical Education Dynamic and Inclusive for 2030: International Curriculum Analysis [Internet]. 2019 [cited 2020 Jul 5]. Available from: https://www.oecd.org/education/2030-project/contact/OECD_FUTURE_OF_EDUCATION_2030_MAKING_PHYSICAL_DYNAMIC_AND_INCLUSIVE_FOR_2030.pdf  3. The Australian Council for Health, Physical Education and Recreation (ACHPER). ACHPER NATIONAL POSITION STATEMENT: Support of the Australian Curriculum: Health and Physical Education [Internet]. 2017 [cited 2020 Jul 13]. Available from: https://www.achper.org.au/documents/item/393  4. The common Wealth. Enhancing the Contribution of Sport to the Sustainable Development Goals [Internet]. OECD Publishing; 2017 [cited 2020 Jul 5]. Available from: http://www.thecommonwealth-ilibrary.org/commonwealth/development/enhancing-the-contribution-of-sport-to-the-sustainable-development-goals_9781848599598-en |
|  |  | 8 -  Discussion on environmental sustainability (n=1) | 1. OLIVEIRA ARC de, SARTORI SK, LAURINDO E. Recomendações para a Educação Física escolar. Sistema CONFEF/CREFs Conselhos Federal e Regionais de Educação Física. 2014; |
| The need for the inclusion components that improve classes and school community participation | 8 -  Curriculum designed with specific PE learning goals and school community participation | 9 -  Curriculum designed with specific PE learning goals and school community participation (n=4) | 1. Bogden JF. Fit, Healthy, and Ready To Learn: A School Health Policy Guide. Part I: Physical Activity, Health Eating, and Tobacco-Use Prevention. 2000 Mar;1–230.  2. UNESCO. Diretrizes em educação física de qualidade (EFQ) para gestores de políticas [Internet]. 2015 [cited 2020 Jul 5]. Available from: https://unesdoc.unesco.org/ark:/48223/pf0000231963  3. (17) (PDF) Global forum for physical education pedagogy 2010 (GoFPEP 2010) statement of consensus:: pedagogy of health and physical education in the xxith century [Internet]. ResearchGate. [cited 2020 Jun 17]. Available from: https://www.researchgate.net/publication/317462682_Global_forum_for_physical_education_pedagogy_2010_GoFPEP_2010_statement_of_consensus_pedagogy_of_health_and_physical_education_in_the_xxith_century  4. OLIVEIRA ARC de, SARTORI SK, LAURINDO E. Recomendações para a Educação Física escolar. Sistema CONFEF/CREFs Conselhos Federal e Regionais de Educação Física. 2014;  5. Association for Physical Education. Health Position Paper afPE 2020 [Internet]. 2020 [cited 2020 Jul 5]. Available from: https://www.afpe.org.uk/physical-education/wp-content/uploads/Health-Position-Paper-2020-Web.pdf |
|  | 9 - Inclusion of components that improve lessons (e.g., learning and assessment goals) | 10 - Inclusion of components that improve lessons (e.g., learning and assessment goals) (n=4) | 1. Centers for Disease Control and Prevention. The Association Between School-Based Physical Activity, Including Physical Education, and Academic Performance. 2010.  2. Organisation for Economic Co-operation and Development (OECD). Making Physical Education Dynamic and Inclusive for 2030: International Curriculum Analysis [Internet]. 2019 [cited 2020 Jul 5]. Available from: https://www.oecd.org/education/2030-project/contact/OECD_FUTURE_OF_EDUCATION_2030_MAKING_PHYSICAL_DYNAMIC_AND_INCLUSIVE_FOR_2030.pdf  3. The common Wealth. Sport for Development and Peace and the 2030 Agenda for Sustainable Development [Internet]. 2015 [cited 2020 Jul 5]. Available from: https://thecommonwealth.org/sites/default/files/inline/CW_SDP_2030%2BAgenda.pdf  4. U.S. Department of Health and Human Services. 2018 Physical Activity Guidelines Advisory Committee [Internet]. 2018 [cited 2020 Jul 5]. Available from: https://health.gov/sites/default/files/2019-09/PAG_Advisory_Committee_Report.pdf |
| The need for the inclusion of content beyond sports | 10 - The need for the inclusion of content beyond sports | 11 - Inclusion of content by age group: games and play, sports initiation, and general sports/exercises (n=4) | 1. Directrices de Evaluación de Niñas, Niños y Adolescentes para la Actividad Física Pedagógica Recreativa y Deportiva Escolar en Paraguay. Pediatr (Asunción). 2012 Apr;39(1):47–56.  2. OLIVEIRA ARC de, SARTORI SK, LAURINDO E. Recomendações para a Educação Física escolar. Sistema CONFEF/CREFs Conselhos Federal e Regionais de Educação Física. 2014;  3. SHAPE America. Opportunity to Learn: Guidelines for Elementary, Middle & High School Physical Education - A Side‐by‐Side Comparison [Internet]. 2010 [cited 2020 Jul 5]. Available from: https://www.shapeamerica.org/standards/guidelines/upload/Opportunity-to-Learn-Grid.pdf |
|  |  | 12 - The need for the inclusion of content beyond sports (n=3) | 1. UNESCO. International Charter of Physical Education, Physical Activity and Sport [Internet]. 2015 [cited 2020 Jul 5]. Available from: https://unesdoc.unesco.org/ark:/48223/pf0000235409  2. Lee SM. School health guidelines to promote healthy eating and physical activity. Morbidity and Mortality Weekly Report. 2011;60(RR-5):1–78.  3. UNESCO. Diretrizes em educação física de qualidade (EFQ) para gestores de políticas [Internet]. 2015 [cited 2020 Jul 5]. Available from: https://unesdoc.unesco.org/ark:/48223/pf0000231963 |

| **APPROPRIATION INSTRUCTION** | | | |
| --- | --- | --- | --- |
| **Recommendations** | | | **References** |
| Use strategies to promote PA and develop student physical literacy, fitness as well as mental, emotional, and social aspects | 1 - Develop several content and actions that encourage PA beyond the classroom | 1 - Content development through diversified classes and based on institutional recommendations and age group (n=7) | 1. Centers for Disease Control and Prevention. A CDC Review of School Laws and Policies Concerning Child and Adolescent Health. Journal of School Health. 2008 Feb;78(2):69–128.  2. UNESCO. International Charter of Physical Education, Physical Activity and Sport [Internet]. 2015 [cited 2020 Jul 5]. Available from: https://unesdoc.unesco.org/ark:/48223/pf0000235409  3. (17) (PDF) Global forum for physical education pedagogy 2010 (GoFPEP 2010) statement of consensus:: pedagogy of health and physical education in the xxith century [Internet]. ResearchGate. [cited 2020 Jun 17]. Available from: https://www.researchgate.net/publication/317462682_Global_forum_for_physical_education_pedagogy_2010_GoFPEP_2010_statement_of_consensus_pedagogy_of_health_and_physical_education_in_the_xxith_century  4. OLIVEIRA ARC de, SARTORI SK, LAURINDO E. Recomendações para a Educação Física escolar. Sistema CONFEF/CREFs Conselhos Federal e Regionais de Educação Física. 2014;  5. National Association for Sport and Physical Education. A Position Statement from the National Association for Sport and Physical Education: Comprehensive School Physical Activity Program. Strategies: A Journal for Physical and Sport Educators. 2008 Jul;21(6):29–33.  6. Ecuador. Ministerio de salud pública Del Ecuador, Coordinación Nacional de Nutrición. Guia de Actividad Física dirigida al personal de salud II. 2011.  7. Mandigo J, Francis PDN, Lodewyk EDK. Position Paper Physical Literacy for Educators. 2009;13.  8. SHAPE America. Appropriate Instructional Practice Guidelines, K-12: A Side-by-Side Comparison SHAPE America – Society of Health and Physical Educators [Internet]. 2009 [cited 2020 Jul 5]. Available from: https://www.shapeamerica.org/upload/Appropriate-Instructional-Practice-Guidelines-K-12.pdf  9. Centers for Disease Control and Prevention. The Association Between School-Based Physical Activity, Including Physical Education, and Academic Performance. 2010.  10. Bogden JF. Fit, Healthy, and Ready To Learn: A School Health Policy Guide. Part I: Physical Activity, Health Eating, and Tobacco-Use Prevention. 2000 Mar;1–230.  11. The common Wealth. Enhancing the Contribution of Sport to the Sustainable Development Goals [Internet]. OECD Publishing; 2017 [cited 2020 Jul 5]. Available from: http://www.thecommonwealth-ilibrary.org/commonwealth/development/enhancing-the-contribution-of-sport-to-the-sustainable-development-goals_9781848599598-en |
|  |  | 2 - Teaching practices that encourage student participation and develop autonomy, confidence, and age-appropriate (n=19) | 1. Centers for Disease Control and Prevention. A CDC Review of School Laws and Policies Concerning Child and Adolescent Health. Journal of School Health. 2008 Feb;78(2):69–128.  2. OLIVEIRA ARC de, SARTORI SK, LAURINDO E. Recomendações para a Educação Física escolar. Sistema CONFEF/CREFs Conselhos Federal e Regionais de Educação Física. 2014;  3. Bogden JF. Fit, Healthy, and Ready To Learn: A School Health Policy Guide. Part I: Physical Activity, Health Eating, and Tobacco-Use Prevention. 2000 Mar;1–230.  4. The Australian Council for Health, Physical Education and Recreation (ACHPER). ACHPER NATIONAL POSITION STATEMENT: Support of the Australian Curriculum: Health and Physical Education [Internet]. 2017 [cited 2020 Jul 13]. Available from: https://www.achper.org.au/documents/item/393  5. Ecuador. Ministerio de salud pública Del Ecuador, Coordinación Nacional de Nutrición. Guia de Actividad Física dirigida al personal de salud II. 2011.  6. International Council of Sport Science and Physical EducationScience Education Policy. International Position Statement on Physical Education. 2010.  7. SHAPE America. Appropriate Instructional Practice Guidelines, K-12: A Side-by-Side Comparison SHAPE America – Society of Health and Physical Educators [Internet]. 2009 [cited 2020 Jul 5]. Available from: https://www.shapeamerica.org/upload/Appropriate-Instructional-Practice-Guidelines-K-12.pdf  8. The common Wealth. Model indicators on sport, physical education and physical activity and the Sustainable Development Goals [Internet]. 2019 [cited 2020 Jul 5]. Available from: https://thecommonwealth.org/sites/default/files/inline/Sport%20and%20SDG%20Indicators%20v3.1.pdf  9. UNESCO. Declaration of Berlin: International Conference of Ministers and Senior Officials Responsible for Physical Education and Sport [Internet]. 2013 [cited 2020 Jul 5]. Available from: https://unesdoc.unesco.org/ark:/48223/pf0000221114  10. UNESCO. International Charter of Physical Education, Physical Activity and Sport [Internet]. 2015 [cited 2020 Jul 5]. Available from: https://unesdoc.unesco.org/ark:/48223/pf0000235409  11. UNESCO. Diretrizes em educação física de qualidade (EFQ) para gestores de políticas [Internet]. 2015 [cited 2020 Jul 5]. Available from: https://unesdoc.unesco.org/ark:/48223/pf0000231963  12. Mandigo J, Francis PDN, Lodewyk EDK. Position Paper Physical Literacy for Educators. 2009;13.  13. The common Wealth. Measuring the contribution of sport, physical education and physical activity to the Sustainable Development Goals Toolkit and model indicators [Internet]. 2019 [cited 2020 Jul 5]. Available from: https://thecommonwealth.org/sites/default/files/inline/Sport-SDGs-Indicator-Framework.pdf  14. Lee SM. School health guidelines to promote healthy eating and physical activity. Morbidity and Mortality Weekly Report. 2011;60(RR-5):1–78.  15. SHAPE America. Instructional Framework for Fitness Education In Physical Education [Internet]. 2012 [cited 2020 Jul 5]. Available from: https://www.shapeamerica.org/standards/guidelines/upload/Instructional-Framework-for-Fitness-Education-in-Physical-Education.pdf  16. (17) (PDF) Global forum for physical education pedagogy 2010 (GoFPEP 2010) statement of consensus:: pedagogy of health and physical education in the xxith century [Internet]. ResearchGate. [cited 2020 Jun 17]. Available from: https://www.researchgate.net/publication/317462682_Global_forum_for_physical_education_pedagogy_2010_GoFPEP_2010_statement_of_consensus_pedagogy_of_health_and_physical_education_in_the_xxith_century  17. Pate RR, Davis MG, Robinson TN, Stone EJ, McKenzie TL, Young JC. Promoting physical activity in children and youth: A leadership role for schools - A scientific statement from the American Heart Association Council on Nutrition, Physical Activity, and Metabolism (Physical Activity Committee) in collaboration with the Councils on Cardiovascular Disease in the Young and Cardiovascular Nursing. Circulation. 2006;114(11):1214–24.  18. SHAPE America. Opportunity to Learn: Guidelines for Elementary, Middle & High School Physical Education - A Side‐by‐Side Comparison [Internet]. 2010 [cited 2020 Jul 5]. Available from: https://www.shapeamerica.org/standards/guidelines/upload/Opportunity-to-Learn-Grid.pdf  19. SHAPE America-Society of Health and Physical Educators. The essential components of physical education. Author Reston, VA; 2015.  20. SHAPE America. Shape of the Nation Status of Physical Education in the USA [Internet]. 2016 [cited 2020 Jul 5]. Available from: https://www.shapeamerica.org/advocacy/son/2016/upload/Shape-of-the-Nation-2016_web.pdf |
|  |  | 3 - The teacher must provide opportunities for student participation in actions that encourage PA practice beyond the class (n=2) | 1. OLIVEIRA ARC de, SARTORI SK, LAURINDO E. Recomendações para a Educação Física escolar. Sistema CONFEF/CREFs Conselhos Federal e Regionais de Educação Física. 2014;  2. UNESCO. Diretrizes em educação física de qualidade (EFQ) para gestores de políticas [Internet]. 2015 [cited 2020 Jul 5]. Available from: https://unesdoc.unesco.org/ark:/48223/pf0000231963  3. SHAPE America. Appropriate Instructional Practice Guidelines, K-12: A Side-by-Side Comparison SHAPE America – Society of Health and Physical Educators [Internet]. 2009 [cited 2020 Jul 5]. Available from: https://www.shapeamerica.org/upload/Appropriate-Instructional-Practice-Guidelines-K-12.pdf |
|  | 2 - Promote opportunities to increase physically active participation in order to improve physical fitness and literacy | 4 - Use of strategies that keep students active for most of the class (n=12) | 1. Centers for Disease Control and Prevention. A CDC Review of School Laws and Policies Concerning Child and Adolescent Health. Journal of School Health. 2008 Feb;78(2):69–128.  2. Bogden JF. Fit, Healthy, and Ready To Learn: A School Health Policy Guide. Part I: Physical Activity, Health Eating, and Tobacco-Use Prevention. 2000 Mar;1–230.  3. Pate RR, Davis MG, Robinson TN, Stone EJ, McKenzie TL, Young JC. Promoting physical activity in children and youth: A leadership role for schools - A scientific statement from the American Heart Association Council on Nutrition, Physical Activity, and Metabolism (Physical Activity Committee) in collaboration with the Councils on Cardiovascular Disease in the Young and Cardiovascular Nursing. Circulation. 2006;114(11):1214–24.  4. SHAPE America. Appropriate Instructional Practice Guidelines, K-12: A Side-by-Side Comparison SHAPE America – Society of Health and Physical Educators [Internet]. 2009 [cited 2020 Jul 5]. Available from: https://www.shapeamerica.org/upload/Appropriate-Instructional-Practice-Guidelines-K-12.pdf  5. SHAPE America-Society of Health and Physical Educators. The essential components of physical education. Author Reston, VA; 2015.  6. SHAPE America. Guide for the Physical Education Policy [Internet]. 2016 [cited 2020 Jul 5]. Available from: https://www.shapeamerica.org/advocacy/upload/Guide-for-Physical-Education-Policy-9-23-14.pdf  7. Hayman LL, Williams CL, Daniels SR, Steinberger J, Paridon S, Dennison BA, et al. Cardiovascular health promotion in the schools: a statement for health and education professionals and child health advocates from the Committee on Atherosclerosis, Hypertension, and Obesity in Youth (AHOY) of the Council on Cardiovascular Disease in the Young, American Heart Association. Circulation. 2004/10/13 ed. 2004 Oct 12;110(15):2266–75.  8. U.S. Department of Health and Human Services. 2018 Physical Activity Guidelines Advisory Committee [Internet]. 2018 [cited 2020 Jul 5]. Available from: https://health.gov/sites/default/files/2019-09/PAG_Advisory_Committee_Report.pdf  9. OLIVEIRA ARC de, SARTORI SK, LAURINDO E. Recomendações para a Educação Física escolar. Sistema CONFEF/CREFs Conselhos Federal e Regionais de Educação Física. 2014;  10. Mandigo J, Francis PDN, Lodewyk EDK. Position Paper Physical Literacy for Educators. 2009;13.  11. Ecuador. Ministerio de salud pública Del Ecuador, Coordinación Nacional de Nutrición. Guia de Actividad Física dirigida al personal de salud II. 2011.  12. SHAPE America. Getting to Know Your Child’s PE Program: A Parent’s Guide [Internet]. 2019 [cited 2020 Jul 5]. Available from: https://www.shapeamerica.org/uploads/pdfs/2017/downloads/eguides/Parent_Checklist.pdf  13. Rütten A, Pfeifer K, Banzer W, Ferrari N, Füzéki E, Geidl W, et al. National Recommendations for Physical Activity and Physical Activity Promotion [Internet]. 2016 [cited 2020 Jul 3]. Available from: https://opus4.kobv.de/opus4-fau/frontdoor/index/index/docId/7827  14. SHAPE America. Opportunity to Learn: Guidelines for Elementary, Middle & High School Physical Education - A Side‐by‐Side Comparison [Internet]. 2010 [cited 2020 Jul 5]. Available from: https://www.shapeamerica.org/standards/guidelines/upload/Opportunity-to-Learn-Grid.pdf  15. Pate RR, O’Neill JR. Summary of the American Heart Association scientific statement: promoting physical activity in children and youth: a leadership role for schools. The Journal of cardiovascular nursing. 2007/12/26 ed. 2008 Jan;23(1):44–9.  16. SHAPE America. Shape of the Nation Status of Physical Education in the USA [Internet]. 2016 [cited 2020 Jul 5]. Available from: https://www.shapeamerica.org/advocacy/son/2016/upload/Shape-of-the-Nation-2016_web.pdf  17. American Heart Association. Increasing and Improving Physical Education and Physical Activity in Schools: Benefits for Children’s Health and Educational Outcomes [Internet]. 2015 [cited 2020 Jul 5]. Available from: https://www.heart.org/idc/groups/heart-public/@wcm/@adv/documents/downloadable/ucm_473782.pdf  18. SHAPE America. Instructional Framework for Fitness Education In Physical Education [Internet]. 2012 [cited 2020 Jul 5]. Available from: https://www.shapeamerica.org/standards/guidelines/upload/Instructional-Framework-for-Fitness-Education-in-Physical-Education.pdf  19. Lee SM. School health guidelines to promote healthy eating and physical activity. Morbidity and Mortality Weekly Report. 2011;60(RR-5):1–78.  20. Increasing physical activity. A report on recommendations of the Task Force on Community Preventive Services. MMWR Recommendations and reports : Morbidity and mortality weekly report Recommendations and reports. 2001/11/09 ed. 2001 Oct 26;50(Rr-18):1–14. |
|  |  | 5 - Inclusion of strategies to improve physical fitness, addressing concepts related to PA (n=12) | 1. Bogden JF. Fit, Healthy, and Ready To Learn: A School Health Policy Guide. Part I: Physical Activity, Health Eating, and Tobacco-Use Prevention. 2000 Mar;1–230.  2. Mandigo J, Francis PDN, Lodewyk EDK. Position Paper Physical Literacy for Educators. 2009;13.  3. Directrices de Evaluación de Niñas, Niños y Adolescentes para la Actividad Física Pedagógica Recreativa y Deportiva Escolar en Paraguay. Pediatr (Asunción). 2012 Apr;39(1):47–56.  4. Ministerio del Deporte, Gobierno de Chile. Política Nacional de Actividad Física y Deporte 2016-2025 [Internet]. 2016 [cited 2020 Jul 2]. Available from: http://www.mindep.cl/wp-content/uploads/2015/05/POLITICA-ULTIMA-VERSI%C3%93N-021116.pdf  5. Lee SM. School health guidelines to promote healthy eating and physical activity. Morbidity and Mortality Weekly Report. 2011;60(RR-5):1–78.  6. SHAPE America. Instructional Framework for Fitness Education In Physical Education [Internet]. 2012 [cited 2020 Jul 5]. Available from: https://www.shapeamerica.org/standards/guidelines/upload/Instructional-Framework-for-Fitness-Education-in-Physical-Education.pdf  7. SHAPE America. Appropriate Instructional Practice Guidelines, K-12: A Side-by-Side Comparison SHAPE America – Society of Health and Physical Educators [Internet]. 2009 [cited 2020 Jul 5]. Available from: https://www.shapeamerica.org/upload/Appropriate-Instructional-Practice-Guidelines-K-12.pdf  8. Association for Physical Education. Health Position Paper afPE 2020 [Internet]. 2020 [cited 2020 Jul 5]. Available from: https://www.afpe.org.uk/physical-education/wp-content/uploads/Health-Position-Paper-2020-Web.pdf |
|  |  | 6 - Development of physical literacy through enjoyable practices appropriate to the student’s level (n=6) | 1. World Health Organization. Global action plan on physical activity 2018–2030: more active people for a healthier world [Internet]. 2018 [cited 2020 Jul 5]. Available from: https://www.who.int/ncds/prevention/physical-activity/global-action-plan-2018-2030/en/  2. Graf C, Beneke R, Bloch W, Bucksch J, Dordel S, Eiser S, et al. Recommendations for promoting physical activity for children and adolescents in Germany. A consensus statement. Obesity Facts. 2014;7(3):178–90.  3. OLIVEIRA ARC de, SARTORI SK, LAURINDO E. Recomendações para a Educação Física escolar. Sistema CONFEF/CREFs Conselhos Federal e Regionais de Educação Física. 2014;  4. Ministry of Social Affairs and Health. Recommendations for the promotion of physical activity in Finland. 2010.  5. Ecuador. Ministerio de salud pública Del Ecuador, Coordinación Nacional de Nutrición. Guia de Actividad Física dirigida al personal de salud II. 2011.  6. SHAPE America. Appropriate Instructional Practice Guidelines, K-12: A Side-by-Side Comparison SHAPE America – Society of Health and Physical Educators [Internet]. 2009 [cited 2020 Jul 5]. Available from: https://www.shapeamerica.org/upload/Appropriate-Instructional-Practice-Guidelines-K-12.pdf  7. Pate RR, Davis MG, Robinson TN, Stone EJ, McKenzie TL, Young JC. Promoting physical activity in children and youth: A leadership role for schools - A scientific statement from the American Heart Association Council on Nutrition, Physical Activity, and Metabolism (Physical Activity Committee) in collaboration with the Councils on Cardiovascular Disease in the Young and Cardiovascular Nursing. Circulation. 2006;114(11):1214–24.  8. SHAPE America. Opportunity to Learn: Guidelines for Elementary, Middle & High School Physical Education - A Side‐by‐Side Comparison [Internet]. 2010 [cited 2020 Jul 5]. Available from: https://www.shapeamerica.org/standards/guidelines/upload/Opportunity-to-Learn-Grid.pdf |
|  | 3 - Use of strategies to promote the mental, emotional, and social development of each student | 7 - Use of strategies to promote the mental, emotional, and social development of each student (n=7) | 1. UNESCO. Diretrizes em educação física de qualidade (EFQ) para gestores de políticas [Internet]. 2015 [cited 2020 Jul 5]. Available from: https://unesdoc.unesco.org/ark:/48223/pf0000231963  2. Organisation for Economic Co-operation and Development (OECD). Making Physical Education Dynamic and Inclusive for 2030: International Curriculum Analysis [Internet]. 2019 [cited 2020 Jul 5]. Available from: https://www.oecd.org/education/2030-project/contact/OECD_FUTURE_OF_EDUCATION_2030_MAKING_PHYSICAL_DYNAMIC_AND_INCLUSIVE_FOR_2030.pdf  3. SHAPE America. Appropriate Instructional Practice Guidelines, K-12: A Side-by-Side Comparison SHAPE America – Society of Health and Physical Educators [Internet]. 2009 [cited 2020 Jul 5]. Available from: https://www.shapeamerica.org/upload/Appropriate-Instructional-Practice-Guidelines-K-12.pdf  4. Centers for Disease Control and Prevention. A CDC Review of School Laws and Policies Concerning Child and Adolescent Health. Journal of School Health. 2008 Feb;78(2):69–128.  5. OLIVEIRA ARC de, SARTORI SK, LAURINDO E. Recomendações para a Educação Física escolar. Sistema CONFEF/CREFs Conselhos Federal e Regionais de Educação Física. 2014;  6. Bogden JF. Fit, Healthy, and Ready To Learn: A School Health Policy Guide. Part I: Physical Activity, Health Eating, and Tobacco-Use Prevention. 2000 Mar;1–230.  7. Ministry of Social Affairs and Health. Recommendations for the promotion of physical activity in Finland. 2010.  8. The Australian Council for Health, Physical Education and Recreation (ACHPER). ACHPER NATIONAL POSITION STATEMENT: Support of the Australian Curriculum: Health and Physical Education [Internet]. 2017 [cited 2020 Jul 13]. Available from: https://www.achper.org.au/documents/item/393  9. SHAPE America-Society of Health and Physical Educators. The essential components of physical education. Author Reston, VA; 2015.  10. SHAPE America. Opportunity to Learn: Guidelines for Elementary, Middle & High School Physical Education - A Side‐by‐Side Comparison [Internet]. 2010 [cited 2020 Jul 5]. Available from: https://www.shapeamerica.org/standards/guidelines/upload/Opportunity-to-Learn-Grid.pdf  11. UNESCO. International Charter of Physical Education, Physical Activity and Sport [Internet]. 2015 [cited 2020 Jul 5]. Available from: https://unesdoc.unesco.org/ark:/48223/pf0000235409  12. European Physical Education Association. European Framework of Quality Physical Education [Internet]. 2018 [cited 2020 Jul 5]. Available from: http://www.eupea.com/wp-content/uploads/2018/02/European-Framework-of-Quality-PE.pdf |
| The need to use strategies that discuss important social issues and include all students | 4 - Use of strategies for inclusion of all students, regardless of disability, skill, or other condition | 8 - Use of strategies for inclusion of all students, regardless of disability, skill, or other condition (n=14) | 1. OLIVEIRA ARC de, SARTORI SK, LAURINDO E. Recomendações para a Educação Física escolar. Sistema CONFEF/CREFs Conselhos Federal e Regionais de Educação Física. 2014;  2. Bogden JF. Fit, Healthy, and Ready To Learn: A School Health Policy Guide. Part I: Physical Activity, Health Eating, and Tobacco-Use Prevention. 2000 Mar;1–230.  3. Mandigo J, Francis PDN, Lodewyk EDK. Position Paper Physical Literacy for Educators. 2009;13.  4. Ecuador. Ministerio de salud pública Del Ecuador, Coordinación Nacional de Nutrición. Guia de Actividad Física dirigida al personal de salud II. 2011.  5. SHAPE America. Getting to Know Your Child’s PE Program: A Parent’s Guide [Internet]. 2019 [cited 2020 Jul 5]. Available from: https://www.shapeamerica.org/uploads/pdfs/2017/downloads/eguides/Parent_Checklist.pdf  6. Rütten A, Pfeifer K, Banzer W, Ferrari N, Füzéki E, Geidl W, et al. National Recommendations for Physical Activity and Physical Activity Promotion [Internet]. 2016 [cited 2020 Jul 3]. Available from: https://opus4.kobv.de/opus4-fau/frontdoor/index/index/docId/7827  7. Lee SM. School health guidelines to promote healthy eating and physical activity. Morbidity and Mortality Weekly Report. 2011;60(RR-5):1–78.  8. Increasing physical activity. A report on recommendations of the Task Force on Community Preventive Services. MMWR Recommendations and reports : Morbidity and mortality weekly report Recommendations and reports. 2001/11/09 ed. 2001 Oct 26;50(Rr-18):1–14.  9. International Council of Sport Science and Physical EducationScience Education Policy. International Position Statement on Physical Education. 2010.  10. SHAPE America. Appropriate Instructional Practice Guidelines, K-12: A Side-by-Side Comparison SHAPE America – Society of Health and Physical Educators [Internet]. 2009 [cited 2020 Jul 5]. Available from: https://www.shapeamerica.org/upload/Appropriate-Instructional-Practice-Guidelines-K-12.pdf  11. The common Wealth. Model indicators on sport, physical education and physical activity and the Sustainable Development Goals [Internet]. 2019 [cited 2020 Jul 5]. Available from: https://thecommonwealth.org/sites/default/files/inline/Sport%20and%20SDG%20Indicators%20v3.1.pdf  12. UNESCO. Diretrizes em educação física de qualidade (EFQ) para gestores de políticas [Internet]. 2015 [cited 2020 Jul 5]. Available from: https://unesdoc.unesco.org/ark:/48223/pf0000231963  13. Council on Sports Medicine and Fitness and Council on School Health. Active healthy living: prevention of childhood obesity through increased physical activity. Pediatrics. 2006/05/03 ed. 2006 May;117(5):1834–42.  14. SHAPE America-Society of Health and Physical Educators. The essential components of physical education. Author Reston, VA; 2015.  15. SHAPE America. Shape of the Nation Status of Physical Education in the USA [Internet]. 2016 [cited 2020 Jul 5]. Available from: https://www.shapeamerica.org/advocacy/son/2016/upload/Shape-of-the-Nation-2016_web.pdf  16. SHAPE America. Opportunity to Learn: Guidelines for Elementary, Middle & High School Physical Education - A Side‐by‐Side Comparison [Internet]. 2010 [cited 2020 Jul 5]. Available from: https://www.shapeamerica.org/standards/guidelines/upload/Opportunity-to-Learn-Grid.pdf  17. Association for Physical Education. Health Position Paper afPE 2020 [Internet]. 2020 [cited 2020 Jul 5]. Available from: https://www.afpe.org.uk/physical-education/wp-content/uploads/Health-Position-Paper-2020-Web.pdf |
|  | 5 - Include social themes linked to the movement behaviors | 9 - Inclusion of relevant social issues that are linked to daily practices (n=19) | 1. Centers for Disease Control and Prevention. A CDC Review of School Laws and Policies Concerning Child and Adolescent Health. Journal of School Health. 2008 Feb;78(2):69–128.  2. OLIVEIRA ARC de, SARTORI SK, LAURINDO E. Recomendações para a Educação Física escolar. Sistema CONFEF/CREFs Conselhos Federal e Regionais de Educação Física. 2014;  3. Bogden JF. Fit, Healthy, and Ready To Learn: A School Health Policy Guide. Part I: Physical Activity, Health Eating, and Tobacco-Use Prevention. 2000 Mar;1–230.  4. The Australian Council for Health, Physical Education and Recreation (ACHPER). ACHPER NATIONAL POSITION STATEMENT: Support of the Australian Curriculum: Health and Physical Education [Internet]. 2017 [cited 2020 Jul 13]. Available from: https://www.achper.org.au/documents/item/393  5. Ecuador. Ministerio de salud pública Del Ecuador, Coordinación Nacional de Nutrición. Guia de Actividad Física dirigida al personal de salud II. 2011.  6. International Council of Sport Science and Physical EducationScience Education Policy. International Position Statement on Physical Education. 2010.  7. SHAPE America. Appropriate Instructional Practice Guidelines, K-12: A Side-by-Side Comparison SHAPE America – Society of Health and Physical Educators [Internet]. 2009 [cited 2020 Jul 5]. Available from: https://www.shapeamerica.org/upload/Appropriate-Instructional-Practice-Guidelines-K-12.pdf  8. The common Wealth. Model indicators on sport, physical education and physical activity and the Sustainable Development Goals [Internet]. 2019 [cited 2020 Jul 5]. Available from: https://thecommonwealth.org/sites/default/files/inline/Sport%20and%20SDG%20Indicators%20v3.1.pdf  9. UNESCO. Declaration of Berlin: International Conference of Ministers and Senior Officials Responsible for Physical Education and Sport [Internet]. 2013 [cited 2020 Jul 5]. Available from: https://unesdoc.unesco.org/ark:/48223/pf0000221114  10. UNESCO. International Charter of Physical Education, Physical Activity and Sport [Internet]. 2015 [cited 2020 Jul 5]. Available from: https://unesdoc.unesco.org/ark:/48223/pf0000235409  11. UNESCO. Diretrizes em educação física de qualidade (EFQ) para gestores de políticas [Internet]. 2015 [cited 2020 Jul 5]. Available from: https://unesdoc.unesco.org/ark:/48223/pf0000231963  12. Mandigo J, Francis PDN, Lodewyk EDK. Position Paper Physical Literacy for Educators. 2009;13.  13. The common Wealth. Measuring the contribution of sport, physical education and physical activity to the Sustainable Development Goals Toolkit and model indicators [Internet]. 2019 [cited 2020 Jul 5]. Available from: https://thecommonwealth.org/sites/default/files/inline/Sport-SDGs-Indicator-Framework.pdf  14. Lee SM. School health guidelines to promote healthy eating and physical activity. Morbidity and Mortality Weekly Report. 2011;60(RR-5):1–78.  15. SHAPE America. Instructional Framework for Fitness Education In Physical Education [Internet]. 2012 [cited 2020 Jul 5]. Available from: https://www.shapeamerica.org/standards/guidelines/upload/Instructional-Framework-for-Fitness-Education-in-Physical-Education.pdf  16. (17) (PDF) Global forum for physical education pedagogy 2010 (GoFPEP 2010) statement of consensus:: pedagogy of health and physical education in the xxith century [Internet]. ResearchGate. [cited 2020 Jun 17]. Available from: https://www.researchgate.net/publication/317462682_Global_forum_for_physical_education_pedagogy_2010_GoFPEP_2010_statement_of_consensus_pedagogy_of_health_and_physical_education_in_the_xxith_century  17. Pate RR, Davis MG, Robinson TN, Stone EJ, McKenzie TL, Young JC. Promoting physical activity in children and youth: A leadership role for schools - A scientific statement from the American Heart Association Council on Nutrition, Physical Activity, and Metabolism (Physical Activity Committee) in collaboration with the Councils on Cardiovascular Disease in the Young and Cardiovascular Nursing. Circulation. 2006;114(11):1214–24.  18. SHAPE America. Opportunity to Learn: Guidelines for Elementary, Middle & High School Physical Education - A Side‐by‐Side Comparison [Internet]. 2010 [cited 2020 Jul 5]. Available from: https://www.shapeamerica.org/standards/guidelines/upload/Opportunity-to-Learn-Grid.pdf  19. SHAPE America-Society of Health and Physical Educators. The essential components of physical education. Author Reston, VA; 2015.  20. SHAPE America. Shape of the Nation Status of Physical Education in the USA [Internet]. 2016 [cited 2020 Jul 5]. Available from: https://www.shapeamerica.org/advocacy/son/2016/upload/Shape-of-the-Nation-2016_web.pdf |
|  |  | 10 - Inclusion of movement behaviors content as a means of expanding students' knowledge about culture and body expression (n=6) | 1. OLIVEIRA ARC de, SARTORI SK, LAURINDO E. Recomendações para a Educação Física escolar. Sistema CONFEF/CREFs Conselhos Federal e Regionais de Educação Física. 2014;  2. Bogden JF. Fit, Healthy, and Ready To Learn: A School Health Policy Guide. Part I: Physical Activity, Health Eating, and Tobacco-Use Prevention. 2000 Mar;1–230.  3. Federation Internationale d´Éducation Physique. The World Manifest of Physical Education FIEP 2000 [Internet]. 2000 [cited 2020 Jul 5]. Available from: http://fiepeurope.eu/manifest.php  4. (17) (PDF) Global forum for physical education pedagogy 2010 (GoFPEP 2010) statement of consensus:: pedagogy of health and physical education in the xxith century [Internet]. ResearchGate. [cited 2020 Jun 17]. Available from: https://www.researchgate.net/publication/317462682_Global_forum_for_physical_education_pedagogy_2010_GoFPEP_2010_statement_of_consensus_pedagogy_of_health_and_physical_education_in_the_xxith_century  5. SHAPE America. Appropriate Instructional Practice Guidelines, K-12: A Side-by-Side Comparison SHAPE America – Society of Health and Physical Educators [Internet]. 2009 [cited 2020 Jul 5]. Available from: https://www.shapeamerica.org/upload/Appropriate-Instructional-Practice-Guidelines-K-12.pdf  6. SHAPE America. Opportunity to Learn: Guidelines for Elementary, Middle & High School Physical Education - A Side‐by‐Side Comparison [Internet]. 2010 [cited 2020 Jul 5]. Available from: https://www.shapeamerica.org/standards/guidelines/upload/Opportunity-to-Learn-Grid.pdf |
| The need to use innovative approaches and technologies and well-maintained equipment and materials | 6 - Use innovative approaches and technologies to enable interaction with students | 11 - Use of innovative technologies and approaches in class development and assessment (n=2) | 1. Organisation for Economic Co-operation and Development (OECD). Making Physical Education Dynamic and Inclusive for 2030: International Curriculum Analysis [Internet]. 2019 [cited 2020 Jul 5]. Available from: https://www.oecd.org/education/2030-project/contact/OECD_FUTURE_OF_EDUCATION_2030_MAKING_PHYSICAL_DYNAMIC_AND_INCLUSIVE_FOR_2030.pdf  2. OLIVEIRA ARC de, SARTORI SK, LAURINDO E. Recomendações para a Educação Física escolar. Sistema CONFEF/CREFs Conselhos Federal e Regionais de Educação Física. 2014;  3. SHAPE America. Appropriate Instructional Practice Guidelines, K-12: A Side-by-Side Comparison SHAPE America – Society of Health and Physical Educators [Internet]. 2009 [cited 2020 Jul 5]. Available from: https://www.shapeamerica.org/upload/Appropriate-Instructional-Practice-Guidelines-K-12.pdf  4. SHAPE America. Opportunity to Learn: Guidelines for Elementary, Middle & High School Physical Education - A Side‐by‐Side Comparison [Internet]. 2010 [cited 2020 Jul 5]. Available from: https://www.shapeamerica.org/standards/guidelines/upload/Opportunity-to-Learn-Grid.pdf  5. (17) (PDF) Global forum for physical education pedagogy 2010 (GoFPEP 2010) statement of consensus:: pedagogy of health and physical education in the xxith century [Internet]. ResearchGate. [cited 2020 Jun 17]. Available from: https://www.researchgate.net/publication/317462682_Global_forum_for_physical_education_pedagogy_2010_GoFPEP_2010_statement_of_consensus_pedagogy_of_health_and_physical_education_in_the_xxith_century |
|  |  | 12- Teachers must keep up to date and propose pedagogical strategies that favor interaction with students (n=1) | 1. OLIVEIRA ARC de, SARTORI SK, LAURINDO E. Recomendações para a Educação Física escolar. Sistema CONFEF/CREFs Conselhos Federal e Regionais de Educação Física. 2014;  2. SHAPE America. Appropriate Instructional Practice Guidelines, K-12: A Side-by-Side Comparison SHAPE America – Society of Health and Physical Educators [Internet]. 2009 [cited 2020 Jul 5]. Available from: https://www.shapeamerica.org/upload/Appropriate-Instructional-Practice-Guidelines-K-12.pdf |
|  | 7 - The teacher must use materials and equipment in good condition | 13 - The teacher must use materials and equipment in good condition (n=5) | 1. Directrices de Evaluación de Niñas, Niños y Adolescentes para la Actividad Física Pedagógica Recreativa y Deportiva Escolar en Paraguay. Pediatr (Asunción). 2012 Apr;39(1):47–56.  2. SHAPE America. Opportunity to Learn: Guidelines for Elementary, Middle & High School Physical Education - A Side‐by‐Side Comparison [Internet]. 2010 [cited 2020 Jul 5]. Available from: https://www.shapeamerica.org/standards/guidelines/upload/Opportunity-to-Learn-Grid.pdf  3. Federation Internationale d´Éducation Physique. The World Manifest of Physical Education FIEP 2000 [Internet]. 2000 [cited 2020 Jul 5]. Available from: http://fiepeurope.eu/manifest.php  4. SHAPE America. Appropriate Instructional Practice Guidelines, K-12: A Side-by-Side Comparison SHAPE America – Society of Health and Physical Educators [Internet]. 2009 [cited 2020 Jul 5]. Available from: https://www.shapeamerica.org/upload/Appropriate-Instructional-Practice-Guidelines-K-12.pdf  5. Mandigo J, Francis PDN, Lodewyk EDK. Position Paper Physical Literacy for Educators. 2009;13. |
| The need to organize the teaching–learning process systematically and present it to the school community | 8 - Organize the teaching-learning process systematically | 14 - Systematic organization of the teaching-learning process (objectives, contents, dimensions, activities, materials and assessments) (n=7) | 1. OLIVEIRA ARC de, SARTORI SK, LAURINDO E. Recomendações para a Educação Física escolar. Sistema CONFEF/CREFs Conselhos Federal e Regionais de Educação Física. 2014;  2. SHAPE America. Appropriate Instructional Practice Guidelines, K-12: A Side-by-Side Comparison SHAPE America – Society of Health and Physical Educators [Internet]. 2009 [cited 2020 Jul 5]. Available from: https://www.shapeamerica.org/upload/Appropriate-Instructional-Practice-Guidelines-K-12.pdf |
|  |  | 15 - Classes should follow basic recommendations such as warm-up, activities appropriate to student development, and instruction to help the student perform more efficiently (n=3) | 1. Directrices de Evaluación de Niñas, Niños y Adolescentes para la Actividad Física Pedagógica Recreativa y Deportiva Escolar en Paraguay. Pediatr (Asunción). 2012 Apr;39(1):47–56.  2. SHAPE America. Appropriate Instructional Practice Guidelines, K-12: A Side-by-Side Comparison SHAPE America – Society of Health and Physical Educators [Internet]. 2009 [cited 2020 Jul 5]. Available from: https://www.shapeamerica.org/upload/Appropriate-Instructional-Practice-Guidelines-K-12.pdf  3. European Physical Education Association. European Framework of Quality Physical Education [Internet]. 2018 [cited 2020 Jul 5]. Available from: http://www.eupea.com/wp-content/uploads/2018/02/European-Framework-of-Quality-PE.pdf |
|  | 9 - The teacher must present to the students, parents, and managers the goals, learning objectives, and evaluation process of the discipline | 16 - The teacher must present to the students, parents, and managers the goals, learning objectives and evaluation process of the discipline (n=3) | 1. SHAPE America. Appropriate Instructional Practice Guidelines, K-12: A Side-by-Side Comparison SHAPE America – Society of Health and Physical Educators [Internet]. 2009 [cited 2020 Jul 5]. Available from: https://www.shapeamerica.org/upload/Appropriate-Instructional-Practice-Guidelines-K-12.pdf  2. European Physical Education Association. European Framework of Quality Physical Education [Internet]. 2018 [cited 2020 Jul 5]. Available from: http://www.eupea.com/wp-content/uploads/2018/02/European-Framework-of-Quality-PE.pdf  3. Organisation for Economic Co-operation and Development (OECD). Making Physical Education Dynamic and Inclusive for 2030: International Curriculum Analysis [Internet]. 2019 [cited 2020 Jul 5]. Available from: https://www.oecd.org/education/2030-project/contact/OECD_FUTURE_OF_EDUCATION_2030_MAKING_PHYSICAL_DYNAMIC_AND_INCLUSIVE_FOR_2030.pdf  4. Lloyd RS, Faigenbaum AD, Stone MH, Oliver JL, Jeffreys I, Moody JA, et al. Position statement on youth resistance training: the 2014 International Consensus. British Journal of Sports Medicine. 2014;48(7):498–505. |
| **STUDENT ASSESSMENT** | | | |
| **Recommendations** | | | **References** |
| The need to understand human movement concepts and achieving an integral development | 1 - Understand movement concepts and develop motor skills | 1 - Understand movement concepts and develop motor skills (n=2) | 1. Centers for Disease Control and Prevention. A CDC Review of School Laws and Policies Concerning Child and Adolescent Health. Journal of School Health. 2008 Feb;78(2):69–128.  2. Bogden JF. Fit, Healthy, and Ready To Learn: A School Health Policy Guide. Part I: Physical Activity, Health Eating, and Tobacco-Use Prevention. 2000 Mar;1–230. |
|  | 2 - Progression in cognitive, affective, social, motor, and biological aspects and in participation in PA | 2 - Assess individual progress (n=3) | 1. Centers for Disease Control and Prevention. A CDC Review of School Laws and Policies Concerning Child and Adolescent Health. Journal of School Health. 2008 Feb;78(2):69–128.  2. Bogden JF. Fit, Healthy, and Ready To Learn: A School Health Policy Guide. Part I: Physical Activity, Health Eating, and Tobacco-Use Prevention. 2000 Mar;1–230.  3. SHAPE America. Opportunity to Learn: Guidelines for Elementary, Middle & High School Physical Education - A Side‐by‐Side Comparison [Internet]. 2010 [cited 2020 Jul 5]. Available from: https://www.shapeamerica.org/standards/guidelines/upload/Opportunity-to-Learn-Grid.pdf |
|  |  | 3 - Present responsible personal and social behavior that respects you and others in PA environments (n=1) | 1. Centers for Disease Control and Prevention. A CDC Review of School Laws and Policies Concerning Child and Adolescent Health. Journal of School Health. 2008 Feb;78(2):69–128. |
|  |  | 4 - Assess cognitive, affective, biological, and motor aspects (n=1) | 1. OLIVEIRA ARC de, SARTORI SK, LAURINDO E. Recomendações para a Educação Física escolar. Sistema CONFEF/CREFs Conselhos Federal e Regionais de Educação Física. 2014;  2. SHAPE America. Appropriate Instructional Practice Guidelines, K-12: A Side-by-Side Comparison SHAPE America – Society of Health and Physical Educators [Internet]. 2009 [cited 2020 Jul 5]. Available from: https://www.shapeamerica.org/upload/Appropriate-Instructional-Practice-Guidelines-K-12.pdf  3. SHAPE America. Opportunity to Learn: Guidelines for Elementary, Middle & High School Physical Education - A Side‐by‐Side Comparison [Internet]. 2010 [cited 2020 Jul 5]. Available from: https://www.shapeamerica.org/standards/guidelines/upload/Opportunity-to-Learn-Grid.pdf |
|  |  | 5 - Develop a positive self-concept, without criticisms from others (n=1) | 1. SHAPE America. Appropriate Instructional Practice Guidelines, K-12: A Side-by-Side Comparison SHAPE America – Society of Health and Physical Educators [Internet]. 2009 [cited 2020 Jul 5]. Available from: https://www.shapeamerica.org/upload/Appropriate-Instructional-Practice-Guidelines-K-12.pdf |
| The need to follow identical requirement levels of other disciplines, with evaluations consistent with policy contents and standards | 3 - Physical fitness should not be used to define academic performance | 6 - Physical fitness should not be used to define academic performance (n=5) | 1. Centers for Disease Control and Prevention. A CDC Review of School Laws and Policies Concerning Child and Adolescent Health. Journal of School Health. 2008 Feb;78(2):69–128.  2. American Heart Association. Increasing and Improving Physical Education and Physical Activity in Schools: Benefits for Children’s Health and Educational Outcomes [Internet]. 2015 [cited 2020 Jul 5]. Available from: https://www.heart.org/idc/groups/heart-public/@wcm/@adv/documents/downloadable/ucm_473782.pdf  3. Bogden JF. Fit, Healthy, and Ready To Learn: A School Health Policy Guide. Part I: Physical Activity, Health Eating, and Tobacco-Use Prevention. 2000 Mar;1–230.  4. SHAPE America. Appropriate Instructional Practice Guidelines, K-12: A Side-by-Side Comparison SHAPE America – Society of Health and Physical Educators [Internet]. 2009 [cited 2020 Jul 5]. Available from: https://www.shapeamerica.org/upload/Appropriate-Instructional-Practice-Guidelines-K-12.pdf  5. Lee SM. School health guidelines to promote healthy eating and physical activity. Morbidity and Mortality Weekly Report. 2011;60(RR-5):1–78. |
|  | 4 - Evaluation consistent with content and standards that allow monitoring of PE goals | 7 - Evaluation consistent with the content and allowing the monitoring of the established objectives (n=9) | 1. National Association for Sport and Physical Education. A Position Statement from the National Association for Sport and Physical Education: Comprehensive School Physical Activity Program. Strategies: A Journal for Physical and Sport Educators. 2008 Jul;21(6):29–33.  2. SHAPE America-Society of Health and Physical Educators. The essential components of physical education. Author Reston, VA; 2015.  3. SHAPE America. Shape of the Nation Status of Physical Education in the USA [Internet]. 2016 [cited 2020 Jul 5]. Available from: https://www.shapeamerica.org/advocacy/son/2016/upload/Shape-of-the-Nation-2016_web.pdf  4. Bogden JF. Fit, Healthy, and Ready To Learn: A School Health Policy Guide. Part I: Physical Activity, Health Eating, and Tobacco-Use Prevention. 2000 Mar;1–230.  5. OLIVEIRA ARC de, SARTORI SK, LAURINDO E. Recomendações para a Educação Física escolar. Sistema CONFEF/CREFs Conselhos Federal e Regionais de Educação Física. 2014;  6. Lee SM. School health guidelines to promote healthy eating and physical activity. Morbidity and Mortality Weekly Report. 2011;60(RR-5):1–78.  7. SHAPE America. Opportunity to Learn: Guidelines for Elementary, Middle & High School Physical Education - A Side‐by‐Side Comparison [Internet]. 2010 [cited 2020 Jul 5]. Available from: https://www.shapeamerica.org/standards/guidelines/upload/Opportunity-to-Learn-Grid.pdf  8. SHAPE America. Appropriate Instructional Practice Guidelines, K-12: A Side-by-Side Comparison SHAPE America – Society of Health and Physical Educators [Internet]. 2009 [cited 2020 Jul 5]. Available from: https://www.shapeamerica.org/upload/Appropriate-Instructional-Practice-Guidelines-K-12.pdf  9. UNESCO. Diretrizes em educação física de qualidade (EFQ) para gestores de políticas [Internet]. 2015 [cited 2020 Jul 5]. Available from: https://unesdoc.unesco.org/ark:/48223/pf0000231963  10. Department of Health (Ireland). Get Ireland Active! The National Physical Activity Plan for Ireland [Internet]. 2016 [cited 2020 Jul 5]. Available from: https://assets.gov.ie/12198/5f3dbab207f2464bba3b9b3f6d02bff6.pdf |
|  |  | 8 - Evaluation consistent with national, state, and municipal standards (n=6) | 1. (17) (PDF) Global forum for physical education pedagogy 2010 (GoFPEP 2010) statement of consensus:: pedagogy of health and physical education in the xxith century [Internet]. ResearchGate. [cited 2020 Jun 17]. Available from: https://www.researchgate.net/publication/317462682_Global_forum_for_physical_education_pedagogy_2010_GoFPEP_2010_statement_of_consensus_pedagogy_of_health_and_physical_education_in_the_xxith_century  2. Lee SM. School health guidelines to promote healthy eating and physical activity. Morbidity and Mortality Weekly Report. 2011;60(RR-5):1–78.  3. SHAPE America. Opportunity to Learn: Guidelines for Elementary, Middle & High School Physical Education - A Side‐by‐Side Comparison [Internet]. 2010 [cited 2020 Jul 5]. Available from: https://www.shapeamerica.org/standards/guidelines/upload/Opportunity-to-Learn-Grid.pdf  4. SHAPE America-Society of Health and Physical Educators. The essential components of physical education. Author Reston, VA; 2015.  5. SHAPE America. Shape of the Nation Status of Physical Education in the USA [Internet]. 2016 [cited 2020 Jul 5]. Available from: https://www.shapeamerica.org/advocacy/son/2016/upload/Shape-of-the-Nation-2016_web.pdf  6. European Physical Education Association. European Framework of Quality Physical Education [Internet]. 2018 [cited 2020 Jul 5]. Available from: http://www.eupea.com/wp-content/uploads/2018/02/European-Framework-of-Quality-PE.pdf  7. The common Wealth. Sport for Development and Peace and the 2030 Agenda for Sustainable Development [Internet]. 2015 [cited 2020 Jul 5]. Available from: https://thecommonwealth.org/sites/default/files/inline/CW_SDP_2030%2BAgenda.pdf  8. National Physical Activity Plan Alliance. National Physical Activity Plan Alliance. U.S. National Physical Activity Plan. 2016 [Internet]. 2016 [cited 2020 Jul 5]. Available from: https://www.physicalactivityplan.org/docs/2016NPAP_Finalforwebsite.pdf |
|  |  | 9 - Monitor and evaluate schools for PE goals (n=1) | 1. Australian 24-Hour Movement Guidelines for Children (5-12 years) and Young People (13-17 years): An Integration of Physical Activity, Sedentary Behaviour, and Sleep. :148. |
|  | 5 - Follow the equivalent rigor as other disciplines, with the participation of the PE teacher | 10 - Follow the equivalent rigor as other disciplines, with the participation of the PE teacher (n=2) | 1. Bogden JF. Fit, Healthy, and Ready To Learn: A School Health Policy Guide. Part I: Physical Activity, Health Eating, and Tobacco-Use Prevention. 2000 Mar;1–230.  2. OLIVEIRA ARC de, SARTORI SK, LAURINDO E. Recomendações para a Educação Física escolar. Sistema CONFEF/CREFs Conselhos Federal e Regionais de Educação Física. 2014; |
| The need to diversify assessment methods to develop the active and healthy lifestyles of students and present their progress to the guardians | 6 - Diversify assessment methods, including collaborative techniques for developing an active and healthy lifestyle | 11 - Use of different assessment techniques to help students learn (n=2) | 1. SHAPE America. Appropriate Instructional Practice Guidelines, K-12: A Side-by-Side Comparison SHAPE America – Society of Health and Physical Educators [Internet]. 2009 [cited 2020 Jul 5]. Available from: https://www.shapeamerica.org/upload/Appropriate-Instructional-Practice-Guidelines-K-12.pdf  2. SHAPE America. Opportunity to Learn: Guidelines for Elementary, Middle & High School Physical Education - A Side‐by‐Side Comparison [Internet]. 2010 [cited 2020 Jul 5]. Available from: https://www.shapeamerica.org/standards/guidelines/upload/Opportunity-to-Learn-Grid.pdf  3. European Physical Education Association. European Framework of Quality Physical Education [Internet]. 2018 [cited 2020 Jul 5]. Available from: http://www.eupea.com/wp-content/uploads/2018/02/European-Framework-of-Quality-PE.pdf |
|  |  | 12 - Inclusion of formative and summative assessment in collaboration with other teachers and students (n=1) | 1. SHAPE America. Appropriate Instructional Practice Guidelines, K-12: A Side-by-Side Comparison SHAPE America – Society of Health and Physical Educators [Internet]. 2009 [cited 2020 Jul 5]. Available from: https://www.shapeamerica.org/upload/Appropriate-Instructional-Practice-Guidelines-K-12.pdf  2. UNESCO. Diretrizes em educação física de qualidade (EFQ) para gestores de políticas [Internet]. 2015 [cited 2020 Jul 5]. Available from: https://unesdoc.unesco.org/ark:/48223/pf0000231963  3. SHAPE America. Opportunity to Learn: Guidelines for Elementary, Middle & High School Physical Education - A Side‐by‐Side Comparison [Internet]. 2010 [cited 2020 Jul 5]. Available from: https://www.shapeamerica.org/standards/guidelines/upload/Opportunity-to-Learn-Grid.pdf |
|  |  | 13 - Assessment should aim to develop a healthy lifestyle throughout life (n=1) | 1. SHAPE America. Opportunity to Learn: Guidelines for Elementary, Middle & High School Physical Education - A Side‐by‐Side Comparison [Internet]. 2010 [cited 2020 Jul 5]. Available from: https://www.shapeamerica.org/standards/guidelines/upload/Opportunity-to-Learn-Grid.pdf |
|  | 7 - Report to the guardians the individual progress of the student | 14 - Report to the guardians the individual progress of the student (n=4) | 1. OLIVEIRA ARC de, SARTORI SK, LAURINDO E. Recomendações para a Educação Física escolar. Sistema CONFEF/CREFs Conselhos Federal e Regionais de Educação Física. 2014;  2. SHAPE America. Appropriate Instructional Practice Guidelines, K-12: A Side-by-Side Comparison SHAPE America – Society of Health and Physical Educators [Internet]. 2009 [cited 2020 Jul 5]. Available from: https://www.shapeamerica.org/upload/Appropriate-Instructional-Practice-Guidelines-K-12.pdf  3. UNESCO. Diretrizes em educação física de qualidade (EFQ) para gestores de políticas [Internet]. 2015 [cited 2020 Jul 5]. Available from: https://unesdoc.unesco.org/ark:/48223/pf0000231963  4. SHAPE America-Society of Health and Physical Educators. The essential components of physical education. Author Reston, VA; 2015.  5. SHAPE America. Shape of the Nation Status of Physical Education in the USA [Internet]. 2016 [cited 2020 Jul 5]. Available from: https://www.shapeamerica.org/advocacy/son/2016/upload/Shape-of-the-Nation-2016_web.pdf  6. SHAPE America. Opportunity to Learn: Guidelines for Elementary, Middle & High School Physical Education - A Side‐by‐Side Comparison [Internet]. 2010 [cited 2020 Jul 5]. Available from: https://www.shapeamerica.org/standards/guidelines/upload/Opportunity-to-Learn-Grid.pdf  7. American Heart Association. Increasing and Improving Physical Education and Physical Activity in Schools: Benefits for Children’s Health and Educational Outcomes [Internet]. 2015 [cited 2020 Jul 5]. Available from: https://www.heart.org/idc/groups/heart-public/@wcm/@adv/documents/downloadable/ucm_473782.pdf |

| **STRATEGIES THAT INTERACT WITH PHYSICAL EDUCATION** | | | |
| --- | --- | --- | --- |
| **Recommendations** | | | **References** |
| PE teachers should act as strategy leaders to promote PA in school | 1 - PE teachers should act as strategy leaders to promote PA in school | 1- PE teachers should act as strategy leaders to promote PA in school (N=10) | 1. National Association for Sport and Physical Education. A Position Statement from the National Association for Sport and Physical Education: Comprehensive School Physical Activity Program. Strategies: A Journal for Physical and Sport Educators. 2008 Jul;21(6):29–33.  2. Bogden JF. Fit, Healthy, and Ready To Learn: A School Health Policy Guide. Part I: Physical Activity, Health Eating, and Tobacco-Use Prevention. 2000 Mar;1–230.  3. Incarbone O, Ferrante D, Bazan N, Gonzalez G, Barengo N, Kanfino J. Manual Director de actividad física y salud de la república Argentina. Plan Nacional Argentina Saludable Dirección de Promoción de la Salud y Control de Enfermedades No Transmisibles Ministerio de Salud de la Nación. 2013;8(9).  4. OLIVEIRA ARC de, SARTORI SK, LAURINDO E. Recomendações para a Educação Física escolar. Sistema CONFEF/CREFs Conselhos Federal e Regionais de Educação Física. 2014;  5. SHAPE America. Appropriate Instructional Practice Guidelines, K-12: A Side-by-Side Comparison SHAPE America – Society of Health and Physical Educators [Internet]. 2009 [cited 2020 Jul 5]. Available from: https://www.shapeamerica.org/upload/Appropriate-Instructional-Practice-Guidelines-K-12.pdf  6. SHAPE America. Instructional Framework for Fitness Education In Physical Education [Internet]. 2012 [cited 2020 Jul 5]. Available from: https://www.shapeamerica.org/standards/guidelines/upload/Instructional-Framework-for-Fitness-Education-in-Physical-Education.pdf  7. SHAPE America. Opportunity to Learn: Guidelines for Elementary, Middle & High School Physical Education - A Side‐by‐Side Comparison [Internet]. 2010 [cited 2020 Jul 5]. Available from: https://www.shapeamerica.org/standards/guidelines/upload/Opportunity-to-Learn-Grid.pdf  8. Increasing physical activity. A report on recommendations of the Task Force on Community Preventive Services. MMWR Recommendations and reports : Morbidity and mortality weekly report Recommendations and reports. 2001/11/09 ed. 2001 Oct 26;50(Rr-18):1–14. |
| Schools must provide active environments and encourage family participation | 2 - Schools must provide daily active environments | 2 - Schools must provide daily active environments (N=6) | 1. National Association for Sport and Physical Education. A Position Statement from the National Association for Sport and Physical Education: Comprehensive School Physical Activity Program. Strategies: A Journal for Physical and Sport Educators. 2008 Jul;21(6):29–33.  2. Bogden JF. Fit, Healthy, and Ready To Learn: A School Health Policy Guide. Part I: Physical Activity, Health Eating, and Tobacco-Use Prevention. 2000 Mar;1–230.  3. Council on Sports Medicine and Fitness and Council on School Health. Active healthy living: prevention of childhood obesity through increased physical activity. Pediatrics. 2006/05/03 ed. 2006 May;117(5):1834–42.  4. Pate RR, Davis MG, Robinson TN, Stone EJ, McKenzie TL, Young JC. Promoting physical activity in children and youth: A leadership role for schools - A scientific statement from the American Heart Association Council on Nutrition, Physical Activity, and Metabolism (Physical Activity Committee) in collaboration with the Councils on Cardiovascular Disease in the Young and Cardiovascular Nursing. Circulation. 2006;114(11):1214–24.  5. Pate RR, O’Neill JR. Summary of the American Heart Association scientific statement: promoting physical activity in children and youth: a leadership role for schools. The Journal of cardiovascular nursing. 2007/12/26 ed. 2008 Jan;23(1):44–9.  6. Graf C, Beneke R, Bloch W, Bucksch J, Dordel S, Eiser S, et al. Recommendations for promoting physical activity for children and adolescents in Germany. A consensus statement. Obesity Facts. 2014;7(3):178–90.  7. Association for Physical Education. Health Position Paper afPE 2020 [Internet]. 2020 [cited 2020 Jul 5]. Available from: https://www.afpe.org.uk/physical-education/wp-content/uploads/Health-Position-Paper-2020-Web.pdf |
|  | 3 - Schools should encourage the participation of families in PA | 3 - Schools should encourage the participation of families in PA (N=7) | 1. National Association for Sport and Physical Education. A Position Statement from the National Association for Sport and Physical Education: Comprehensive School Physical Activity Program. Strategies: A Journal for Physical and Sport Educators. 2008 Jul;21(6):29–33.  2. Bogden JF. Fit, Healthy, and Ready To Learn: A School Health Policy Guide. Part I: Physical Activity, Health Eating, and Tobacco-Use Prevention. 2000 Mar;1–230.  3. OLIVEIRA ARC de, SARTORI SK, LAURINDO E. Recomendações para a Educação Física escolar. Sistema CONFEF/CREFs Conselhos Federal e Regionais de Educação Física. 2014;  4. ParticipACTION Advisory Groups. Active Canada 20/20: A physical activity strategy and change agenda for Canada. 2012.  5. Ecuador. Ministerio de salud pública Del Ecuador, Coordinación Nacional de Nutrición. Guia de Actividad Física dirigida al personal de salud II. 2011.  6. SHAPE America. Appropriate Instructional Practice Guidelines, K-12: A Side-by-Side Comparison SHAPE America – Society of Health and Physical Educators [Internet]. 2009 [cited 2020 Jul 5]. Available from: https://www.shapeamerica.org/upload/Appropriate-Instructional-Practice-Guidelines-K-12.pdf  7. SHAPE America-Society of Health and Physical Educators. The essential components of physical education. Author Reston, VA; 2015.  8. SHAPE America. Shape of the Nation Status of Physical Education in the USA [Internet]. 2016 [cited 2020 Jul 5]. Available from: https://www.shapeamerica.org/advocacy/son/2016/upload/Shape-of-the-Nation-2016_web.pdf  9. (17) (PDF) Global forum for physical education pedagogy 2010 (GoFPEP 2010) statement of consensus:: pedagogy of health and physical education in the xxith century [Internet]. ResearchGate. [cited 2020 Jun 17]. Available from: https://www.researchgate.net/publication/317462682_Global_forum_for_physical_education_pedagogy_2010_GoFPEP_2010_statement_of_consensus_pedagogy_of_health_and_physical_education_in_the_xxith_century  10. Ministerio del Deporte, Gobierno de Chile. Política Nacional de Actividad Física y Deporte 2016-2025 [Internet]. 2016 [cited 2020 Jul 2]. Available from: http://www.mindep.cl/wp-content/uploads/2015/05/POLITICA-ULTIMA-VERSI%C3%93N-021116.pdf  11. Centers for Disease Control and Prevention. Parent Engagement: Strategies for Involving Parents in School Health. 2012.  12. Centers for Disease Control and Prevention. Promoting Parent Engagement: Improving Student Health and Academic Achievement. 2012.  13. Pate RR, Davis MG, Robinson TN, Stone EJ, McKenzie TL, Young JC. Promoting physical activity in children and youth: A leadership role for schools - A scientific statement from the American Heart Association Council on Nutrition, Physical Activity, and Metabolism (Physical Activity Committee) in collaboration with the Councils on Cardiovascular Disease in the Young and Cardiovascular Nursing. Circulation. 2006;114(11):1214–24.  14. Lee SM. School health guidelines to promote healthy eating and physical activity. Morbidity and Mortality Weekly Report. 2011;60(RR-5):1–78.  15. Centers for Disease Control and Prevention. Parents for Healthy Schools: A Guide for Getting Parents Involved from K–12. 2019;26.  16. Graf C, Beneke R, Bloch W, Bucksch J, Dordel S, Eiser S, et al. Recommendations for promoting physical activity for children and adolescents in Germany. A consensus statement. Obesity Facts. 2014;7(3):178–90. |
|  | 4 - Parents should monitor curriculum evaluation and participate in actions that support PE | 4 - Parents should monitor curriculum evaluation and participate in actions that support PE (n=4) | 1. Centers for Disease Control and Prevention. Parents for Healthy Schools: A Guide for Getting Parents Involved from K–12. 2019;26. |
| Action plans supporting an active and healthy lifestyle should be promoted at school, encouraging integration among different subjects | 5 - Health policies must be created in schools with a defined action plan supporting an active and healthy lifestyle | 5 - Health policies must be created in schools with a defined action plan supporting an active and healthy lifestyle (n=10) | 1. National Association for Sport and Physical Education. A Position Statement from the National Association for Sport and Physical Education: Comprehensive School Physical Activity Program. Strategies: A Journal for Physical and Sport Educators. 2008 Jul;21(6):29–33.  2. (17) (PDF) Global forum for physical education pedagogy 2010 (GoFPEP 2010) statement of consensus:: pedagogy of health and physical education in the xxith century [Internet]. ResearchGate. [cited 2020 Jun 17]. Available from: https://www.researchgate.net/publication/317462682_Global_forum_for_physical_education_pedagogy_2010_GoFPEP_2010_statement_of_consensus_pedagogy_of_health_and_physical_education_in_the_xxith_century  3. Hayman LL, Williams CL, Daniels SR, Steinberger J, Paridon S, Dennison BA, et al. Cardiovascular health promotion in the schools: a statement for health and education professionals and child health advocates from the Committee on Atherosclerosis, Hypertension, and Obesity in Youth (AHOY) of the Council on Cardiovascular Disease in the Young, American Heart Association. Circulation. 2004/10/13 ed. 2004 Oct 12;110(15):2266–75.  4. Bogden JF. Fit, Healthy, and Ready To Learn: A School Health Policy Guide. Part I: Physical Activity, Health Eating, and Tobacco-Use Prevention. 2000 Mar;1–230.  5. OLIVEIRA ARC de, SARTORI SK, LAURINDO E. Recomendações para a Educação Física escolar. Sistema CONFEF/CREFs Conselhos Federal e Regionais de Educação Física. 2014;  6. ParticipACTION Advisory Groups. Active Canada 20/20: A physical activity strategy and change agenda for Canada. 2012.  7. Ecuador. Ministerio de salud pública Del Ecuador, Coordinación Nacional de Nutrición. Guia de Actividad Física dirigida al personal de salud II. 2011.  8. SHAPE America. Appropriate Instructional Practice Guidelines, K-12: A Side-by-Side Comparison SHAPE America – Society of Health and Physical Educators [Internet]. 2009 [cited 2020 Jul 5]. Available from: https://www.shapeamerica.org/upload/Appropriate-Instructional-Practice-Guidelines-K-12.pdf  9. SHAPE America-Society of Health and Physical Educators. The essential components of physical education. Author Reston, VA; 2015.  10. SHAPE America. Shape of the Nation Status of Physical Education in the USA [Internet]. 2016 [cited 2020 Jul 5]. Available from: https://www.shapeamerica.org/advocacy/son/2016/upload/Shape-of-the-Nation-2016_web.pdf  11. Ministerio del Deporte, Gobierno de Chile. Política Nacional de Actividad Física y Deporte 2016-2025 [Internet]. 2016 [cited 2020 Jul 2]. Available from: http://www.mindep.cl/wp-content/uploads/2015/05/POLITICA-ULTIMA-VERSI%C3%93N-021116.pdf  12. Centers for Disease Control and Prevention. Parent Engagement: Strategies for Involving Parents in School Health. 2012.  13. Centers for Disease Control and Prevention. Promoting Parent Engagement: Improving Student Health and Academic Achievement. 2012.  14. Ministry of Social Affairs and Health. Recommendations for the promotion of physical activity in Finland. 2010.  15. Pate RR, Davis MG, Robinson TN, Stone EJ, McKenzie TL, Young JC. Promoting physical activity in children and youth: A leadership role for schools - A scientific statement from the American Heart Association Council on Nutrition, Physical Activity, and Metabolism (Physical Activity Committee) in collaboration with the Councils on Cardiovascular Disease in the Young and Cardiovascular Nursing. Circulation. 2006;114(11):1214–24.  16. Lee SM. School health guidelines to promote healthy eating and physical activity. Morbidity and Mortality Weekly Report. 2011;60(RR-5):1–78.  17. Graf C, Beneke R, Bloch W, Bucksch J, Dordel S, Eiser S, et al. Recommendations for promoting physical activity for children and adolescents in Germany. A consensus statement. Obesity Facts. 2014;7(3):178–90. |
|  | 6 - Integrate PE content with other subjects and consider the knowledge and skills required by the current generation of students | 6 - Link PE content with other disciplines (n=2) | 1. OLIVEIRA ARC de, SARTORI SK, LAURINDO E. Recomendações para a Educação Física escolar. Sistema CONFEF/CREFs Conselhos Federal e Regionais de Educação Física. 2014;  2. Bogden JF. Fit, Healthy, and Ready To Learn: A School Health Policy Guide. Part I: Physical Activity, Health Eating, and Tobacco-Use Prevention. 2000 Mar;1–230.  3. Ecuador. Ministerio de salud pública Del Ecuador, Coordinación Nacional de Nutrición. Guia de Actividad Física dirigida al personal de salud II. 2011.  4. SHAPE America. Appropriate Instructional Practice Guidelines, K-12: A Side-by-Side Comparison SHAPE America – Society of Health and Physical Educators [Internet]. 2009 [cited 2020 Jul 5]. Available from: https://www.shapeamerica.org/upload/Appropriate-Instructional-Practice-Guidelines-K-12.pdf  5. SHAPE America. Opportunity to Learn: Guidelines for Elementary, Middle & High School Physical Education - A Side‐by‐Side Comparison [Internet]. 2010 [cited 2020 Jul 5]. Available from: https://www.shapeamerica.org/standards/guidelines/upload/Opportunity-to-Learn-Grid.pdf  6. Organisation for Economic Co-operation and Development (OECD). Making Physical Education Dynamic and Inclusive for 2030: International Curriculum Analysis [Internet]. 2019 [cited 2020 Jul 5]. Available from: https://www.oecd.org/education/2030-project/contact/OECD_FUTURE_OF_EDUCATION_2030_MAKING_PHYSICAL_DYNAMIC_AND_INCLUSIVE_FOR_2030.pdf |
|  |  | 7 - Integrate knowledge and skills required by the younger generation across all school subjects and disciplines (n=1) | 1. (17) (PDF) Global forum for physical education pedagogy 2010 (GoFPEP 2010) statement of consensus:: pedagogy of health and physical education in the xxith century [Internet]. ResearchGate. [cited 2020 Jun 17]. Available from: https://www.researchgate.net/publication/317462682_Global_forum_for_physical_education_pedagogy_2010_GoFPEP_2010_statement_of_consensus_pedagogy_of_health_and_physical_education_in_the_xxith_century |
